# Ameliorative effect of oregano (*Origanum vulgare*) versus silymarin in experimentally induced hepatic encephalopathy

**DOI:** 10.1038/s41598-022-20412-3

**Published:** 2022-10-25

**Authors:** Eman A. R. Abdelghffar, Heba A. S. El-Nashar, Shaimaa Fayez, Wael A. Obaid, Omayma A. Eldahshan

**Affiliations:** 1 Department of Zoology, Faculty of Science, Ain Shams University, Cairo, Egypt; 2 Department of Pharmacognosy, Faculty of Pharmacy, Ain-Shams University, Cairo, Egypt; 3 Centre of Drug Discovery Research and Development, Ain Shams University, Cairo, Egypt; 4 Department of Biology, College of Science, Taibah University, Al-Madīnah Al-Munawarah, Saudi Arabia

**Keywords:** Biochemistry, Plant sciences, Diseases, Gastroenterology

## Abstract

Hepatic encephalopathy (HE) is a deterioration of brain function in patients suffering from chronic liver disease, cirrhosis as a result of elevated blood ammonia and the production of pseudo-neurotransmitters. Herein, we investigated the chemical composition of hexane extract from *Origanum vulgare* (*O. vulgare*) leaves as well as its possible protective effects against thioacetamide (TAA)-induced HE in rats. GC–MS analysis of the extract revealed tentative identification of twenty-five compounds (82.93%), predominated by cholesten-3-one (27.30%), followed by γ-tocopherol (13.52%), α-tocopherol (5.01%)*, β*-amyrin (5.24%) and *α*-amyrin (4.89%). Albino rats were distributed into seven groups (*n* = 7). G_1_ served as negative control; G_2_ and G_3_ served as controls treated with *O. vulgare* (100 and 200 mg/kg/*p.o* b.w, respectively); G_4_ served as TAA-positive control group (100 mg/kg/day/*i.p.*, three alternative days per week for six weeks); G5, G6, and G7 served as TAA -induced HE rat model that received *O. vulgare* 100, *O. vulgare* 200, and silymarin (100 mg/kg of SILY, as standard drug), respectively. TAA showed depressive and anxiety-like behaviors in forced swimming test (FST) and reduction of cognitive score in elevated plus-maze test (EPMT) as well as impairment of locomotor and exploratory activities in open-field test (OFT). TAA caused a significant decline in body weight gain; however, the relative liver weight and brain water content were statistically increased. TAA-intoxicated rats showed significant increase of serum biomarker enzymes, proinflammatory cytokines, blood ammonia levels, brain serotonin, acetyl cholinesterase and cellular lipid peroxidation with significant decrease of brain dopamine, norepinephrine, antioxidant status. The hepatoprotective/neuro-protective activities of *O. vulgare* was found to be comparable with that of SILY in HE rats model. Where, treatment of TAA-intoxicated rats with *O. vulgare* attenuated anxiety, depressive-related behaviors, and reduced the biochemical changes in HE-induced by TAA. Therefore, *O. vulgare* could be an excellent hepato-/neuroprotective against hepatic injury and HE via improving the oxidative/inflammatory status through its antioxidant and neuro-modulatory properties and its effect is equal to that of SILY.

## Introduction

Hepatic encephalopathy (HE) is a neuropsychiatric syndrome, which is a major complication closely related to acute or chronic liver failure^1–3^. About 60% to 80% of liver cirrhotic patients (caused by viral hepatitis, excess alcoholism, drug intoxication, toxin exposures, and long-term drug abuses) showed minimal overt HE symptoms with serious consequences in their daily life^1,4^.Various mechanisms are involved in the pathogenesis of HE, including changes in neurotransmission due to metabolic changes in liver failure, excessive oxidative/nitrosative stress, mitochondrial permeability transition, systemic inflammatory response, and immune dysfunction^4^. HE is characterized by a wide spectrum of behavioral manifestations including anxiety, depression symptoms, cognitive impairment, psychomotor deficiency, attention deficits, and learning/ memory impairment which might lead to coma and death in severe cases^1,2,5^. The most prominent neurological problem is the formation of cerebral edema leading to an increased intracranial hypertension and predominant brain herniation with extremely high mortality rates^4^. García-Ayllón et al. (2008) reported that the impairment in the cholinergic system in the brain which is induced by liver disease might negatively impact the learning and memory functions^6^.

The mechanism of HE is still unclear, however, hyperammonemia and the downstream consequences of ammonia uptake by astrocytes has been suggested to play the main role in HE pathogenesis and brain edema^1,4^. Oxidative stress and inflammation have likewise been involved in the pathogenesis of acute and chronic liver damage playing a significant role in the molecular pathogenesis of HE^1,4,7^.

Thioacetamide (TAA) is widely used as a selective hepatotoxin, which is used experimentally to induce acute/chronic liver disease and HE^8^. It is a thiono-sulphur-containing compound with several industrial uses, such as a motor fuel stabilizer, and in leather processing, laboratory, textile, and paper industries. When given in single dose, it causes acute liver injury however multiple doses might lead to hepatic cirrhosis and liver tumors^1,4^. TAA is rapidly metabolized in the liver by hepatic microsomal cytochrome p-450 (CYP2E1) to TAA- sulfoxide derivative and further to an unstable highly toxic metabolite (thioacetamide-S-dioxide) that initiates rapid reduction of intracellular GSH, lipid peroxidation, extensive oxidative stress, hyperammonemia, inflammation, and hepatic damage by covalently binding to liver macromolecules^9,10^. Moreover, malnutrition tends to be more common in patients with advanced liver disease and HE. Anxiety, depression, and cognitive/motor deficits are characterized by activation of the inflammatory responses and consequent production of pro-inflammatory cytokines in HE^11,12^. Activation of immune cells by pro-inflammatory cytokines leads to the over production of reactive oxygen species (ROS), which lead to an increase in the levels of lipid peroxides as malondialdehyde^13^. Moreover, excessive pro-inflammatory cytokines production in the initial stage of HE may lead to the aggravation of brain edema, which may participate in the development of HE^7^.

Oregano or wild marjoram (*Origanum vulgare*) has been known as one of the most used aromatic herbs worldwide of the mint family (Lamiaceae), with abundant existence in East Europe, Middle East, Middle Asia, and North America^14^. Its use has been extended as a dry form in the food industry and cosmetics^15^.

Different classes of natural compounds have been isolated from Oregano like essential oils, flavonoids, phenolic acids, triterpenoids, and sterols^16^. Traditionally, oregano has been utilized as carminative, stomachic, emmenagogue, and expectorant, antispasmodic, and for cough and menstrual disorders^17^.Several therapeutic potentials of Oregano such as antimicrobial, antioxidant, antispasmodic, diuretic, stomachic, immunomodulatory, and antimutagenic have published^18^. Interestingly, it exerted a promising hepatoprotective activity against CCl_4_-induced hepatotoxicity in rats by alleviating transaminase, globulin levels, hepatic antioxidant enzymes and lipid peroxidation. CCl_4_ is like TAA, as they are industrial materials and hepatotoxic agents that induced cellular damage may result from either covalent bond formation between its reactive intermediates and cellular macromolecules or from enhanced lipid peroxidation and oxidative stress triggered by free radical intermediates^16^. Oniga et al.^16^ reported that the hepatoprotective of *O. vulgare* ethanolic extract may be caused by the presence of 10 phenolic acids (the most abundant including gentisic, chlorogenic, *p*-coumaric and rosmarinic acids) and 11 flavonoids (such as hyperoside, isoquercitrin, rutin, quercitrin, quercetin and luteolin) which are responsible for the antioxidant effect.

Silymarin (*Silybum marianum*) has been used for centuries as an alternative complementary medicine for the treatment of many liver diseases such as drug-induced hepatic injury, cirrhosis, necrosis^19^, and has also been reported to display anti-inflammatory and neuroprotective effects against many neurodegenerative diseases including Alzheimer’s disease, cerebral ischemia, and Parkinson’s disease^20^. Its hepato- and neuro-protective mechanisms are believed to be due to its antioxidant and tissue regenerative properties by scavenging the free radicals and enhancement of antioxidant defense mechanisms to inhibit oxidative stress^19,21^. Therefore, great attention has been paid for the discovery of new antioxidant and anti-inflammatory agents for the prevention and treatment of acute and chronic liver damages.

This study was designed to evaluate effects of *Origanum vulgare* (*O. vulgare*) on certain animal behaviors and its antioxidant status on liver and brain against TAA-induced HE in rats.

## Material and methods

### Chemicals

Thioacetamide (TAA) was purchased from Sigma-Aldrich Chemical Co. (St Louis, MO, USA). Silymarin was used as the reference drug and purchased from SEDICO pharmaceuticals company (October City, Egypt).

### Plant material and extraction

The whole plant of *O. vulgare* (Lamiaceae/Labiatae) was purchased from a local herbal market in Cairo, Egypt in February 2020. The collection of plant material was established in compliance with the national guidelines. The plant was authenticated by Dr. Usama K. Abdel Hameed, Department of Botany, Faculty of Science, Ain Shams University, Cairo, Egypt. A voucher specimen (PHG-P-OV-345) was deposited at the department of Pharmacognosy, Faculty of Pharmacy, Ain Shams University, Cairo, Egypt. The air-dried plant (2 kg) was grinded into fine particle and extracted with hexane (10 L × 3 times). The pooled extracts were evaporated under reduced pressure at 45 °C till complete dryness to obtain 60 g of a sticky dark green material.

### Gas Chromatography/Mass Spectrometry (GC/MS) analysis

The GC/MS investigation of the extract was performed using a Shimadzu GC-MS-QP 2010 (Koyoto, Japan) equipment with a TRACE GC Ultra Gas Chromatographs (THERMO Scientific Corp., USA), conjugated with a thermo-mass detector. The GC–MS was equipped with a TG-5MS capillary column (30 m × 0.25 mm i.d., 0.25 μm film thickness) (Restek, USA). The capillary column was directly coupled to a quadrupole mass spectrometer (SSQ 7000; Thermo-Finnigan). Analysis of a diluted sample (1% v/v; injected volume = 1 µL) was carried out using helium as carrier gas at a constant flow rate of 1.0 mL/min and a split ratio of 1:15. The oven temperature was adjusted at 80 °C for 2 min (isothermal), then raised 5.0 °C/min to reach 300 °C (programmed) and held for 5 min (isothermal). The injector and detector temperature were held at 280 °C. The mass spectra were obtained by adjusting the following parameters as follow: interface temperature = 280 °C, ion source temperature = 200 °C, electron ionization (EI) mode = 70 eV, using a scan spectral range at *m/z* 35–500. The relative proportions of the hexane extract constituents were expressed as percentages obtained by peak area normalization.

### Tentative identification of key metabolites of the *n*-hexane extract

The components of *n*-hexane extract were tentatively characterized by matching their GC/MS spectra, fragmentation patterns, and retention indices (Kovats indices) to those published in the literature^22–27^*.* The retention indices were calculated relative to a homologous series of *n*-alkanes (C_8_–C_28_) injected under the same conditions. Peak area percent of each compound relative to the area percent of the entire FID chromatogram (100%) was calculated.

### Procurements of animals and groupings

Male Wistar albino rats (aging 7–8 weeks old and weighing 190 ± 10 g) obtained from a breeding stock animal house of the College of Pharmacy, University of Taibah, Saudi Arabia. They were housed in polypropylene cages under standard laboratory conditions (temperature 24 ± 3 °C, 12/12 h dark/light cycle). The animals were acclimatized with a two-weeks for this condition on standard pelleted food from Makarim Al-Wisam Factory-Makkah-Om Al Joud-KSA (Chemical analysis: 14% Protein, 12% Fiber, 2.5% Fat, 28% Starch, Vit A: 7000 IU/Kg, Vit D: 300 IU/Kg, 1.5% Calcium, 0.4% Phosphorous, 0.06% Sodium, 20 mg/kg Zinc, 0.1% Magnesium, 0.6% Potassium, and 7% Ash) and free access of water was provided ad libitum throughout the experiments. The *in-vivo* studies were conducted in accordance with the Guidelines for the Care and Use of Laboratory Animals of the National Institute of Health (NIH Publications No. 8023, revised 1985). This experimental protocol was carried out following ARRIVE guidelines and approved by Institutional Research Ethics Committee in the College of Pharmacy, Taibah University , Saudi Arabia (COPTU-REC-43-20221002).

### Acute toxicity study

This study was carried out as per Organization for Economic Co-operation and Development 423 guidelines (OECD, 2001) using 20 rats (10 males and 10 females). The animals were divided into control group (5 animals/gender) and the treated group (5 animals/gender). In the treated group, the rats were administered *O. vulgare* extract (dissolved in dist. water containing 1% Dimethyl sulfoxide; DMSO) in the limit test dose of 2000 mg/kg by oral gavage and observed continuously for behavioral, neurological, and autonomic profiles over 2 h, and after a period of 24 h, 72 h and thereafter up to 14 days for any lethality, moribund state, or death. The limit test was repeated in another group of rats (*n* = 5) for approximate LD_50_ determination. Two doses (100 and 200 mg/kg; 1/20th and 1/10th, respectively) were found to be safe and selected for further evaluation for hepato- and neuro-protective activities.

### Induction of cirrhosis and hepatic encephalopathy

After acclimatization, HE in the experimental groups was induced by i.p. injections of TAA (100 mg/kg/day) three alternative days per week for six weeks according to previous studies^28^. The vehicle of TAA was physiological saline (0.9% NaCl; 5 mL/kg/day). To prevent hypoglycemia, weight loss, dehydration, electrolyte imbalance and renal failure, animals received orally 10 mg/kg/day of fluid therapy (5% dextrose containing 0.45% saline (NaCl) and 20 mEq/L of potassium chloride (KCl) to all the rats) according to previous study^28^.

### Experimental design

**Figure 1:**
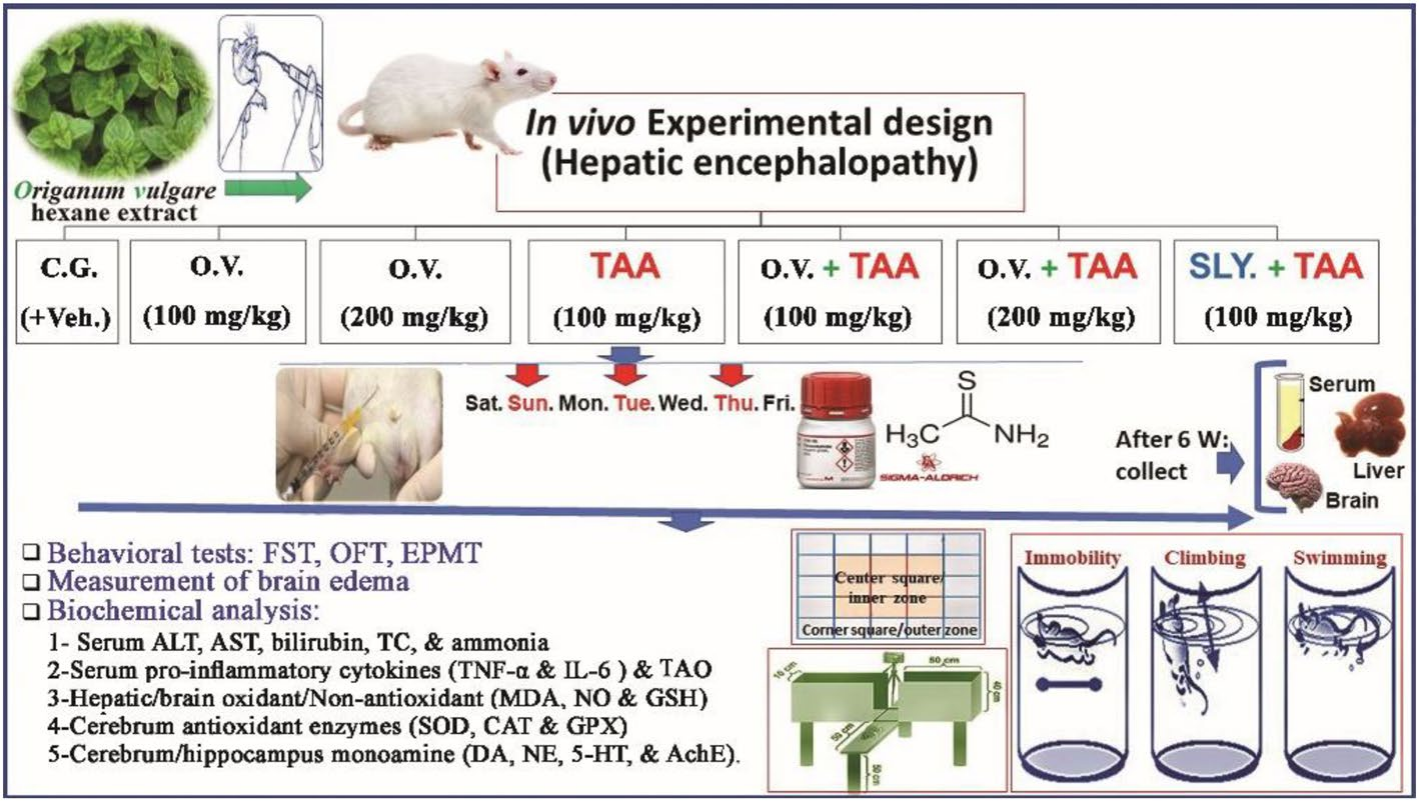
The in vivo experimental design. C.G.: Control group; O.v.:* Origanum vulgare,* SILY: Silymarin, TAA: Thioacetamide, and Veh.: Vehicles.

Forty- nine Wistar albino rats (*Rattus norvegicus*) were randomly distributed into seven groups, each group consisted of seven animals (Fig. 1). Rats of group 1 received the vehicles for 6 weeks and served as control group, i.e., vehicle I—distilled water containing 1% DMSO solution via oral gavage and vehicle II—*i.p.* injection of normal physiological saline (0.9% NaCl). Rats of groups 2 and 3 treated orally with 100 and 200 mg/kg b.w of *O. vulgare* extract alone. These doses also were selected based on previous studies^29,30^, respectively, for 6 weeks. *O. vulgare* extract was dissolved in 1% DMSO solution and it was administered by oral gavage. Rats of group 4 injected with TAA and served as HE-control group. Rats of groups 5 and 6 were orally supplemented with *O. vulgare* extract (100 and 200 mg/kg, respectively) starting one-hour prior an administration of TAA for 6 weeks. Rats of group 7 were orally gavage with silymarin (SILY; 100 mg/kg) based on studies of Shaker et al.^31^ for 6 weeks starting one-hour prior an administration of TAA and served as reference drug group. The behavioral assessments were recorded after 2 h from the last dosing of treatment. The body weight gain or loss during this experiment was measured.

### Measurements

#### Behavioral tests

##### Forced swimming test (FST)

This test is the most widely used model for assessing anti-depressant activity. For this purpose, each rat was dropped into an inescapable glass cylinder tank (60 cm in height and 40 cm in diameter) containing water to a height of 40 cm and are forced to swim for five-minutes. Two-minutes to adapt to the environment and three-minutes to record immobility and active movements (swimming and struggling/climbing) times. Stopping and floating with all limbs without struggling (motionless) on the water surface, making only very slight movements necessary to keep its head above water, showing immobility. Swimming was recorded when the body was moved around the cylinder by fore-paw movements, more than needed to keep the head above the water, showing mobility. Struggling is the movement that took place when the rat struggles to get out of the container by its fore paws breaking the surface of water, usually against the water container wall.

##### Open-field test (OFT)

It was used to estimate anxiety, locomotor, and exploratory activities. The apparatus consisted of a square box (100 × l00 cm, 47 cm height) that was divided by 4 × 4 lines into 25 equal quadrants (20 cm × 20 cm). A single rat was placed in a corner square of the arena and the activity of each was monitored using a video camera for three-minutes and analyzed later. Subsequently, the latency (time spend to enter in the center square indicating anxiety), ambulation/locomotion (the number of squares or lines crossings; indicating locomotory activity), freezing time (total motionless time), grooming frequency (number of body clearing with paws, licking of the body with mouth and washing of the face, body, and genitals) and rearings frequency (number of times the animal stood on its hind limbs; indicating exploratory activity) were counted for three-minutes.

##### Elevated plus-maze test (EPMT)

This test was used to examine the exploratory behavior of rats and anxiolytic/anxiogenic-like drug effects. The elevated plus-maze consisted of two open arms (50 cm length × 10 cm width × 1 cm height), and two closed ones (50 cm length × 10 cm width × 40 cm height) connected by a central platform (10 × 10 cm^2^). The apparatus was elevated 50 cm above the floor. In the beginning of each test, rats were placed individually in the center platform facing one of the open arms. Their behaviors were monitored by the video camera during the three-minutes test period. The time spent in the open and closed arms was measured using stopwatch for the calculation of the percentage of time spent by rats using the following equation.

Percentage time spent in the open or closed arm (%) = [(time spent in the open or closed arm/ total time (180 min)) × 100].

One entry was defined as the entrance of the rat using all its four paws into one arm. The arenas were cleaned after each assay using 70% alcohol to eliminate olfactory bias and the area was allowed to dry before introducing the animal.

#### Measurement of brain edema

Brain water content was determined by the wet/dry weight method. Approximately 10 mg tissue of the cerebral cortex was weighed before and after 48 h incubation in a 120 °C oven^32^. Water content of the brain samples was expressed according to the following equation: Water (%) = [(wet weight – dry weight) / wet weight] × 100.

#### Biochemical analysis

After the behavior tests, rats were anaesthetized prior to blood collection by diethyl ether. Blood was collected from each animal and allowed to coagulate then centrifuged (3000 rpm, 15 min, 4 °C). The obtained serum was divided into aliquots and stored at − 80 °C) until further analysis. Immediately after blood sampling, liver and brain of each animal were rapidly removed, washed in ice-cold saline, dry and weighed. Liver samples were homogenized in 5 mL ice-cooled buffer (0.5 g of Na_2_HPO_4_ and 0.7 g of NaH_2_PO_4_ per 500 mL deionized water, pH 7.4) per gram tissue, while brain samples were removed from the skull and dissected to obtain the cerebrum (cerebral hemisphere) and hippocampus. The cerebrum was then divided into three parts; the 1st one was homogenized in 5 mL ice-cooled phosphate buffer (50 mM pH 7.4, 0.1% tritonX and 0.5 mM EDTA) per gram tissue for biochemical analysis whereas the 2nd part of cerebrum as well as hippocampus (in separate aliquots) were homogenized in an ice-cold solution of acidified *n*-butanol for monoamines neurotransmitter determination. The 3rd part of cerebrum was used for the measurement of brain edema. The homogenates of livers, cerebrum and hippocampus were spun (4000 rpm, 10 min, 4 °C) using a cooling centrifuge to remove cell debris. The aliquots were kept at − 80 °C till the day of analysis. Serum alanine transaminase (ALT; CAT.NO. EP07-500), aspartate transaminase (AST; EP15-500), total cholesterol (CAT.NO. EP24-660), total proteins (CAT.NO. EP56-660), and bilirubin (CAT.NO. EP20-420) were measured using a commercially available assay kit (United Diagnostic Industry, UDI, Off Makkah Road Second Industrial City, Dammam, K.S.A) according to manufacturer’s instructions. Ammonia (NH3; Cat. No. MBS3809074), pro-inflammatory cytokines (TNF-α; CAT.NO. MBS3015754 and IL-6; CAT.NO. MBS824560), and oxidant/non-antioxidant parameters [reduced glutathione (GSH; CAT.NO. MBS8807501), malondialdehyde (MDA; CAT.NO. MBS8807536), and enzymatic parameters [superoxide dismutase (SOD; CAT.NO. MBS036924), glutathione peroxidase (GPX; CAT.NO. MBS744364), catalase (CAT; CAT.NO. MBS9712526)] and total antioxidant capacity (TAO; CAT.NO. MBS2540515)] were measured using a commercially specific rats ELISA kit (MyBioSource, San Diego, California, USA) according to the manufacturers’ recommendations. Also, serotonin (ST; CAT.NO. MBS166089), dopamine (DA; CAT.NO. MBS701755), norepinephrine (NOR; CAT.NO. MBS1600150) and acetylcholinesterase (AChE; CAT.NO. MBS725468) were measured using a commercially specific rats ELISA kit (MyBioSource, San Diego, California, USA). In case hepatic and cerebrum nitric oxide (NO) level, the nitrite concentration in tissue homogenates was measured using the Griess assay^33,34^ to determine the tissue NO level. The results were calculated as mol nitrite/mg protein in the samples. In brief, 100 µl of supernatant was applied to each well of a micotiter plate, 100 µl vanadium (III) chloride (8 mg/mL) was added to each well (to reduce nitrate to nitrite), and then the Griess reagents, 50 µl sulfanilamide (2 percent) and 50 µl N-(1-Naphthyl) ethyl-enediamine di-hydrochloride, were added (0.1 percent). The absorbance was measured at 540 nm using an ELISA reader after 30 min of incubation at room temperature.

### Statistics analysis

The results are presented as the means ± SE. Data were performed using one-way analysis of variance (ANOVA) followed by the Turkey’s post hoc test. Differences with *p* < 0.05 were considered statistically significant. Data were analyzed using a GraphPad Prism software (Graphpad Software, Inc., San Diego, CA, USA).

## Results

### GC–MS analysis of chemical composition of *n*-hexane extract of *O.vulgare*

**Figure 2:**
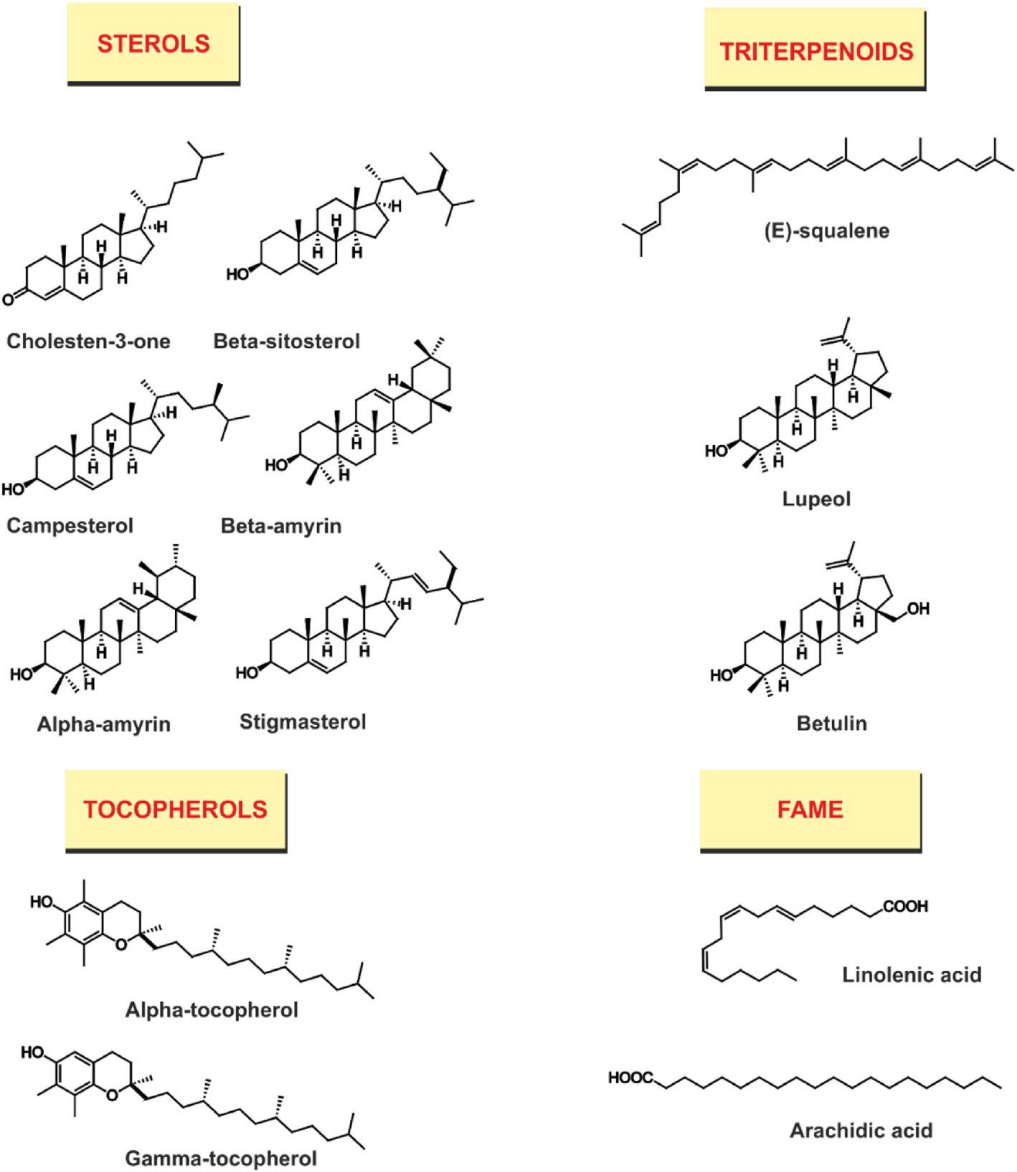
The structures of the main classes of metabolites identified in the *n*-hexane extract of *O. vulgare*.

The GC–MS analysis of the *n*-hexane extract of *O. vulgare* leaves led to the tentative identification of twenty-five compounds constituting about 82.93% of the total extract as illustrated in Table 1 and Fig. 2. Sterols are the predominant class of metabolites representing 37.87%, among them cholesten-3-one (27.30%), *β*-sitosterol (1.3%), campesterol (1.47%), *β*-amyrin (5.24%), *α*-amyrin (4.89%), stigmasterol (1.62%), its acetate derivative (3.31%), its diene form stigmasta-3,5-diene (2.37%), and its ketone representative stigmasta-3,5-dien-7-one (0.5%). Tocopherols are the second major class of compounds with a total of 18.53% including γ-tocopherol (13.52%) and its isomer α-tocopherol (5.01%). Triterpenoids constituted ca. 15.09% of the extract of Organum. Prominent members of this class are *E*-squalene (0.48%), lupeol (3.15%), and betulin (1.81%). Fatty acid methyl esters FAMEs represent 5.18% and include the methyl esters of hexadecanoic acid and linolenic acid. Other free fatty acids include arachidic acid and its methyl derivative. Oxygenated monoterpenes represented 4.48% of the total *n*-hexane extract and include monoterpene alcohols like (*E*)-sabinene hydrate (2.18%), 1-terpinen-4-ol (0.71%), *α*-terpineol (0.55%) and diterpenes like neophytadiene (0.36%).

**Table 1:** Chemical composition (%) of *n*-hexane extract of *O. vulgare leaves* grown in Egypt.

No	Compound^a^	Retention time (t_R_)	Molecular formula	Retention index		Peak area (%)	Method of identification
				Calculated	Reported		
1	(*E*)-Sabinene hydrate	12.680	C_10_H_18_O	1100	1101	2.18	RI, MS
2	1-Terpinen-4-ol	15.195	C_10_H_18_O	1180	1177	0.71	RI, MS
3	*α*-Terpineol	15.615	C_10_H_18_O	1193	1192	0.55	RI, MS
4	4-Terpinenyl acetate	17.470	C_12_H_20_O_2_	1300	1300	1.04	RI, MS
5	Neophytadiene	32.110	C_20_H_38_	1838	1837	0.36	RI, MS
6	Hexadecanoic acid methyl ester	33.965	C_17_H_34_O_2_	1927	1927	0.84	RI, MS
7	Linolenic acid methyl ester	37.475	C_19_H_32_O_2_	2109	2108	0.36	RI, MS
8	Unidentified	40.430	–	–	–	0.83	-
9	Tricosane	41.118	C_23_H_48_	2309	2300	0.56	RI, MS
10	Methyl arachidate	42.030	C_21_H_42_O_2_	2339	2333	2.42	RI, MS
11	Arachidic acid	42.445	C_21_H_42_O_2_	2380	2381	0.38	RI, MS
12	Unidentified	42.697	–	–	–	1.62	–
13	Unidentified	46.094	–	–	–	0.81	–
14	Unidentified	46.703	–	–	–	0.73	–
15	Stigmasta-3,5-diene	47.535	C_29_H_48_	2705	2715	2.37	RI, MS
16	Unidentified	48.137	–	–	–	1.76	–
17	Stigmasta-3,5-dien-7-one	48.550	C_29_H_46_O	2770	2765	0.50	RI, MS
18	(*E*)-Squalene	49.470	C_30_H_50_	2829	2832	0.48	RI, MS
19	Unidentified	50.351	–	–	–	2.51	–
20	Methyl hexacosanoate	51.130	C_27_H_54_O2	2936	2940	1.56	RI, MS
21	Unidentified	51.377	–	–	–	1.48	–
22	Unidentified	51.698	–	–	–	1.43	–
23	Unidentified	52.345	–	–	–	0.41	–
24	Unidentified	52.456	–	–	–	3.96	–
25	Unidentified	52.560	–	–	–	0.74	–
26	*γ*-Tocopherol	53.052	C_29_H_50_O_2_	3059	3065	13.52	RI, MS
27	*α*-Tocopherol	54.090	C_29_H_50_O_2_	3126	3130	5.01	RI, MS
28	Stigmasterol	54.455	C_29_H_48_O	3150	3165	1.62	RI, MS
29	*β*-Sitosterol	55.420	C_29_H_50_O	3212	3202	1.30	RI, MS
30	Unidentified	55.620	–	–	–	0.79	–
31	4-Cholesten-3-one	56.055	C_27_H_44_O	3252	3245	27.30	RI, MS
32	Campesterol	56.850	C_28_H_48_O	3304	3305	1.47	RI, MS
33	Stigmasterol acetate	57.170	C_31_H_50_O_2_	3324	3320	3.31	RI, MS
34	*β*-Amyrin	57.170	C_30_H_50_O	3335	3337	5.24	RI, MS
35	*α*-Amyrin	57.940	C_30_H_50_O	3374	3376	4.89	RI, MS
36	Lupeol	58.805	C_30_H_50_O	3429	3440	3.15	RI, MS
37	Betulin	60.050	C_30_H_50_O_2_	3509	3512	1.81	RI, MS
	Total identified	82.93%					
	Steroids	37.87%					
	Tocopherols	18.53%					
	Triterpenoids	15.09%					
	Fatty acid methyl esters (FAMEs)	5.18%					
	Oxygenated Monoterpenes	4.48%					
	Aliphatic hydrocarbons	0.56%					
	Hydrocarbon Triterpenes	0.48%					
	Fatty acids	0.38%					
	Diterpenes	0.36%					

^a^Compounds are arranged according to their elution. RI: Kovats retention index on DB-5 column. RI, identification of metabolites was based on the comparison of reported Kovats retention indices with the measured ones. MS, identification based on mass spectral data and fragmentation profile.

### The modulatory effects of *O.vulgare* on behavioral tests of the HE rat model

**Figure 3:**
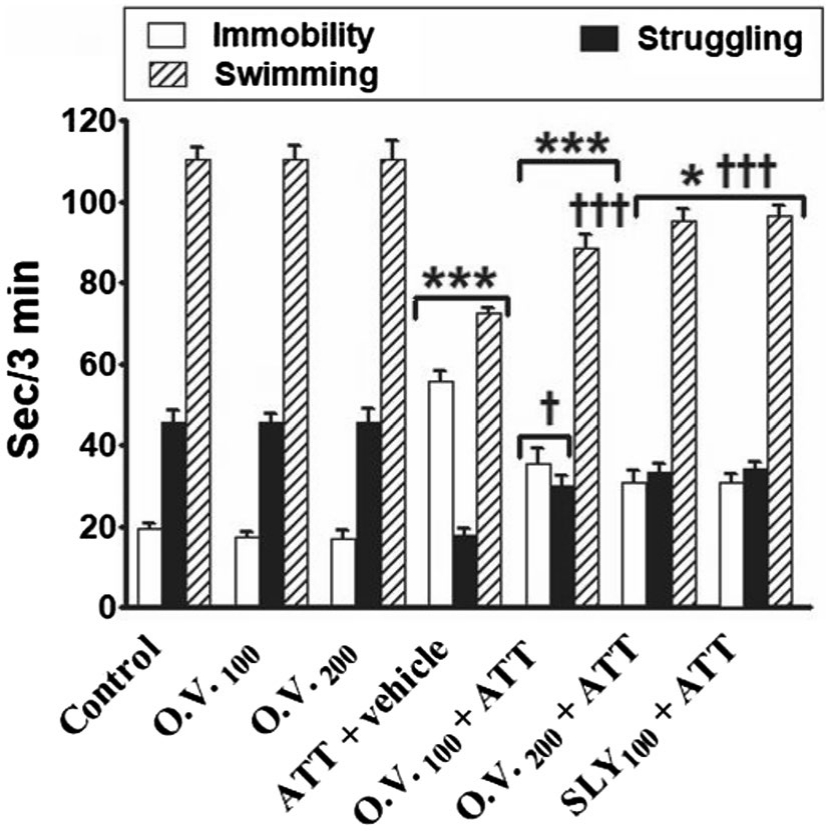
The modulatory effects of *O. vulgare* on immobility, struggling, and swimming behaviors in FST in the HE rat model. SEM represented by vertical bars. O.V*.*: *Origanum vulgare*, SLY: silymarin. *p < 0.05; **p < 0.01; ***p < 0.001 (vs. the negative control group). †*p* < 0.05; ††*p* < 0.01; †††*p* < 0.001 (vs. the HE positive control group, which received vehicle).

In FST (Fig. 3), the immobility time was significantly increased (*p* < 0.05–0.001) in TAA alone, *O. vulgare* 100 + TAA, *O. vulgare* 200 + TAA and SILY + TAA-treated groups (186%, 82%, 58%, and 58%, respectively) compared with the control group. While the swimming and struggling times were significantly decreased (*p* < 0.05–0.001) in TAA alone (− 34% and − 60%, respectively), *O. vulgare* 100 + TAA (− 19% and − 34%, respectively), *O. vulgare* 200 + TAA (− 13% and − 26%, respectively) and SILY + TAA-treated groups (− 12% and − 25%, respectively) compared with the control group. Oral administration of both doses *O. vulgare* + TAA or SILY + TAA significantly increased (*p* < 0.05–0.001) the swimming and struggling times and decreased (*p* < 0.001) the immobility time compared with the TAA alone -treated group.

**Figure 4:**
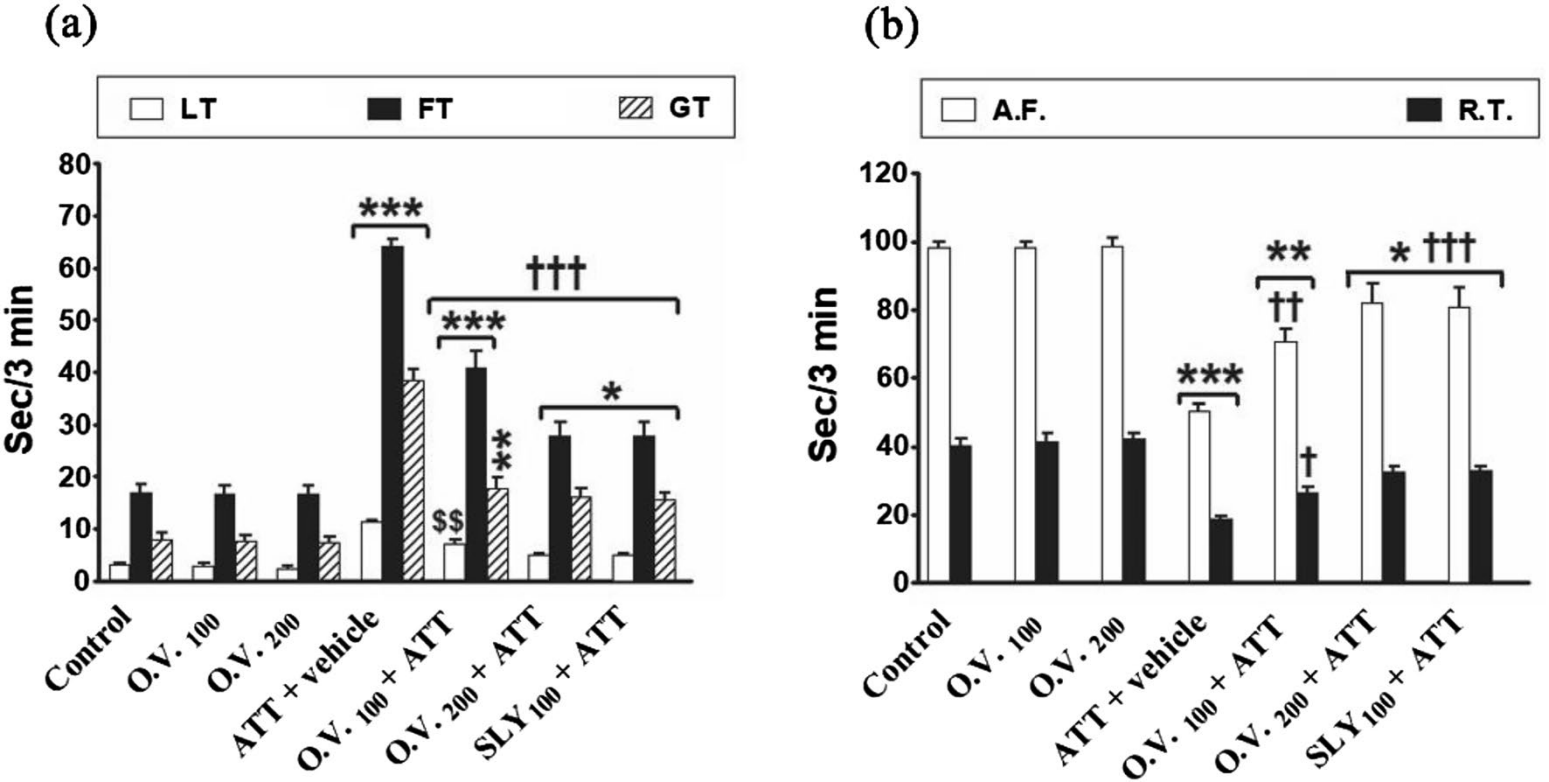
The modulatory effects of *O. vulgare* on latency time, freezing time, and grooming frequency (**a**) and ambulation and rearing frequencies (**b**) in OFT in the HE-induced rat model. SEM represented by vertical bars. AF: ambulation frequency, FT: freezing time, GF: grooming frequency, LT: latency time, O.V*.*: *Origanum vulgare*, RF: rearing frequency, SLY: silymarin. *p < 0.05; **p < 0.01; ***p < 0.001 (vs. the negative control group). †*p* < 0.05; ††*p* < 0.01; †††*p* < 0.001 (vs. the HE positive control group, which received vehicle). $$ *p* < 0.01 (vs. the HE group, which received silymarin).

In OFT as shown in Fig. 4, the latency time to enter in the center square, freezing time, and grooming frequency were considerably increased (*p* < 0.05–0.001) in TAA alone (276%, 277%, and 390%, respectively), *O. vulgare* 100 + TAA (138%, 140%, and 125%, respectively), *O. vulgare* 200 + TAA (62%, 63%, and 103%, respectively), and SILY + TAA (61%, 64%, and 98%, respectively) groups, while ambulation and rearing frequencies were significantly decreased (*p* < 0.05–0.001) in TAA alone (− 48% and − 53%, respectively), *O. vulgare* 100 + TAA (− 28% and − 34%, respectively), *O. vulgare* 200 + TAA (− 16% and − 19%, respectively) and SILY + TAA (− 17% and − 18%, respectively) compared to the control group. Conversely, pretreatment with *O. vulgare* (100 and 200 mg/kg) or SILY produced a significant decline (*p* < 0.001) in latency time, freezing time, and grooming frequency and a significant increase (*p* < 0.05–0.001) in ambulation and rearing frequencies compared with the TAA alone -treated group.

**Figure 5:**
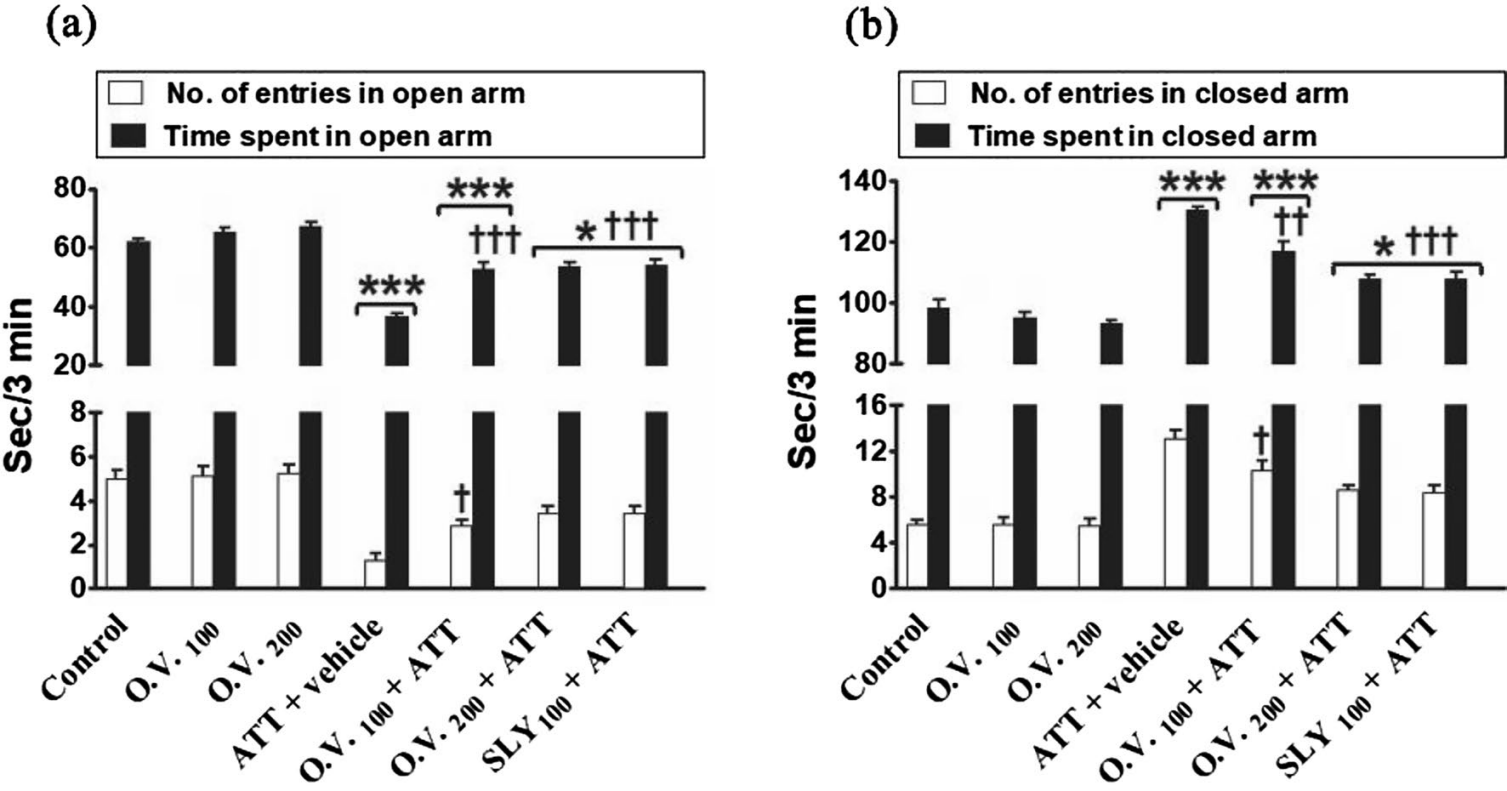
The modulatory effects of *O. vulgare* on the number of entries and the time spent in the open arm (**a**) and closed arm (**b**) in EPMT in the HE-induced rat model. SEM represented by vertical bars. O.V*.*: *Origanum vulgare*, SLY: silymarin. *p < 0.05; ***p < 0.001 (vs. the negative control group). †*p* < 0.05; †††*p* < 0.001 (vs. the HE positive control group, which received vehicle).

In EPMT as shown in Fig. 5, the number of entries and the time spent in the open arm were significantly decreased (p < 0.05–0.001) in TAA alone (− 74 and − 41%, respectively), *O. vulgare* 100 + TAA (− 43 and − 15%, respectively), *O. vulgare* 200 + TAA (− 31 and − 13%, respectively) and SILY + TAA-treated groups (− 31% and − 12%, respectively), while the number of entries and the time spent in the closed arm were significantly increased (*p* < 0.05–0.001) in TAA alone (136% and 33%, respectively), *O. vulgare* 100 + TAA (85% and 19%, respectively), *O. vulgare* 200 + TAA (53% and 10%, respectively) and SILY + TAA-treated groups (51% and 10%, respectively) compared with the control group. On the contrary, pretreatment with *O. vulgare* (100 and 200 mg/kg) or SILY produced a significant increase (*p* < 0.05–0.001) in the number of entries and the time spent in the open arm while pretreatment with *O. vulgare* (100 and 200 mg/kg) or SILY produced a significant decline (*p* < 0.05–0.001) in the number of entries and the time spent in the closed arm compared with the TAA alone-treated group.

### The modulatory effects of *O. vulgare* on the body weight, liver relative weight, and brain water content in the HE-induced rat model

As recorded in Table 2, the body weight gain was significantly decreased (*p* < 0.01–0.001) in TAA alone, *O. vulgare* 100 + TAA, *O. vulgare* 200 + TAA and SILY + TAA-treated groups (7121, 8194, 8619, and 8654%, respectively) in compared with the control group. While liver relative weight was significantly increased (*p* < 0.05–0.001) in TAA alone, *O. vulgare* 100 + TAA, *O. vulgare* 200 + TAA and SILY + TAA-treated groups (180%, 146%, 116%, and 117%, respectively). The brain water content was significantly increased (*p* < 0.05–0.001) in TAA alone, *O. vulgare* 100 + TAA, *O. vulgare* 200 + TAA and SILY + TAA-treated groups (24%, 14%, 12%, and 11%, respectively) compared to the control group. Pretreatment with *O. vulgare* (100 and 200 mg/kg b.w) or SILY produced a significant elevation in the body weight gain and a significant reduction in the liver relative weight as well as the brain water content (*p* < 0.05–0.001) compared with the TAA alone -treated group.

**Table 2:** The modulatory effects of *O. vulgare* on body weight, liver relative weight, and brain water content in the HE-induced rat model.

Parameters	Groups													
	Control		*O. vulgare* 100		*O. vulgare* 200		ATT + vehicle		*O. vulgare* 100 + ATT		*O. vulgare* 200 + ATT		SILY 100 + ATT	
Body weight gain (g)	100.40 ± 3.84		105.2 ± 1.35		106.7 ± 2.18		72.21 ± 2.83***		82.94 ± 1.07 ***†		87.19 ± 2.64 **††		87.54 ± 2.09 **††	
Liver relative weight (g/100 g b.w)	1.79 ± 0.06		1.74 ± 0.08		1.63 ± 0.09		2.80 ± 0.11 ***		2.46 ± 0.06 ***		2.16 ± 0.08 *†††		2.17 ± 0.09 *†††	
Brain water content (%)	62.83 ± 1.4		62.68 ± 2.2		62.62 ± 1.0		78.18 ± 0.5 ***		71.67 ± 1.02 **†		70.37 ± 2.04 *††		69.73 ± 1.08 *††	

Values are means ± SEM. *O. vulgare*: *Origanum vulgare*, SLY: silymarin. *p < 0.05; **p < 0.01; ***p < 0.001 (vs. the negative control group); †*p* < 0.01; ††*p* < 0.01; †††*p* < 0.001 (vs. the HE positive control group, which received vehicle).

### The modulatory effects of *O.vulgare* on serum cellular toxicity markers in the HE-induced rat model

As depicted in Table 3, serum levels of ALT, AST, total bilirubin, total cholesterol, and ammonia were significantly increased (*p* < 0.05–0.001) in TAA alone (269%, 102%, 180%, 108%, and 239%, respectively), *O. vulgare* 100 + TAA (121%, 55%, 23%, 45%, and 148%, respectively), *O. vulgare* 200 + TAA (45%, 21%, 18%, 13%, and 37% respectively), and SILY + TAA-treated groups (46%, 19%, 16%, 13%, and 42% respectively), while serum total protein was significantly decreased (*p* < 0.05–0.001) in TAA alone, *O. vulgare* 100 + TAA, *O. vulgare* 200 + TAA and SILY + TAA-treated groups (− 34%, − 18%, − 14%, and − 13%, respectively) compared with the control group. In contrast, administration of either doses of *O. vulgare* or SILY significantly alleviated serum biochemical alternations (*p* < 0.05–0.001) compared with the TAA alone -treated group.

**Table 3:** The modulatory effects of *O. vulgare* on serum cellular toxicity markers, and ammonia in the HE rat model.

Groups							
Parameters	Control	*O. vulgare* 100	*O. vulgare* 200	ATT + vehicle	*O. vulgare* 100 + ATT	*O. vulgare* 200 + ATT	SILY 100 + ATT
AST(IU/L)	101.60 ± 2.15	100.30 ± 3.36	100.60 ± 3.42	205.4 ± 3.84 ***	157.40 ± 3.92 ***†††$$$	123.00 ± 6.75 *†††	121.30 ± 5.16 *†††
ALT (IU/L)	31.43 ± 2.85	31.43 ± 2.32	31.14 ± 2.20	116.00 ± 3.37 ***	69.57 ± 2.53 ***†††$$$	45.86 ± 4.07 *†††	46.00 ± 2.70 *†††
Total bilirubin (mg/dL)	0.67 ± 0.02	0.66 ± 0.01	0.64 ± 0.02	1.87 ± 0.02 ***	0.82 ± 0.04 **†††	0.79 ± 0.03 *†††	0.78 ± 0.03 *†††
Total protein (g/dL)	7.18 ± 0.16	7.32 ± 0.13	7.38 ± 0.16	4.72 ± 0.29 ***	5.85 ± 0.26 **†	6.14 ± 0.25 *†††	6.07 ± 0.21 *††
Total cholesterol (mg/dL)	97.33 ± 1.84	96.20 ± 1.54	95.76 ± 1.55	202.10 ± 1.98 ***	141.00 ± 4.08 ***†††$$$	110.10 ± 2.75 *†††	109.80 ± 2.28 *†††
Ammonia (μmol/L)	36.29 ± 3.13	35.86 ± 2.37	39.14 ± 2.55	123.30 ± 3.23 ***	90.00 ± 2.18 ***†††$$$	50.00 ± 3.06 *†††	51.57 ± 4.13 *†††

Values are means ± SEM. ALT: alanine aminotransferase, AST: aspartate aminotransferase, *O. vulgare*: *Origanum vulgare*, SLY: silymarin. *p < 0.05; **p < 0.01; ***p < 0.001 (vs. the negative control group); †*p* < 0.01; ††*p* < 0.01; †††*p* < 0.001 (vs. the HE positive control group, which received vehicle). $$$ *p* < 0.001 (vs. the HE group, which received silymarin).

### The modulatory effects of *O.vulgare* on serum pro-inflammatory cytokines and total antioxidants capacity in the HE-induced rat model

**Figure 6:**
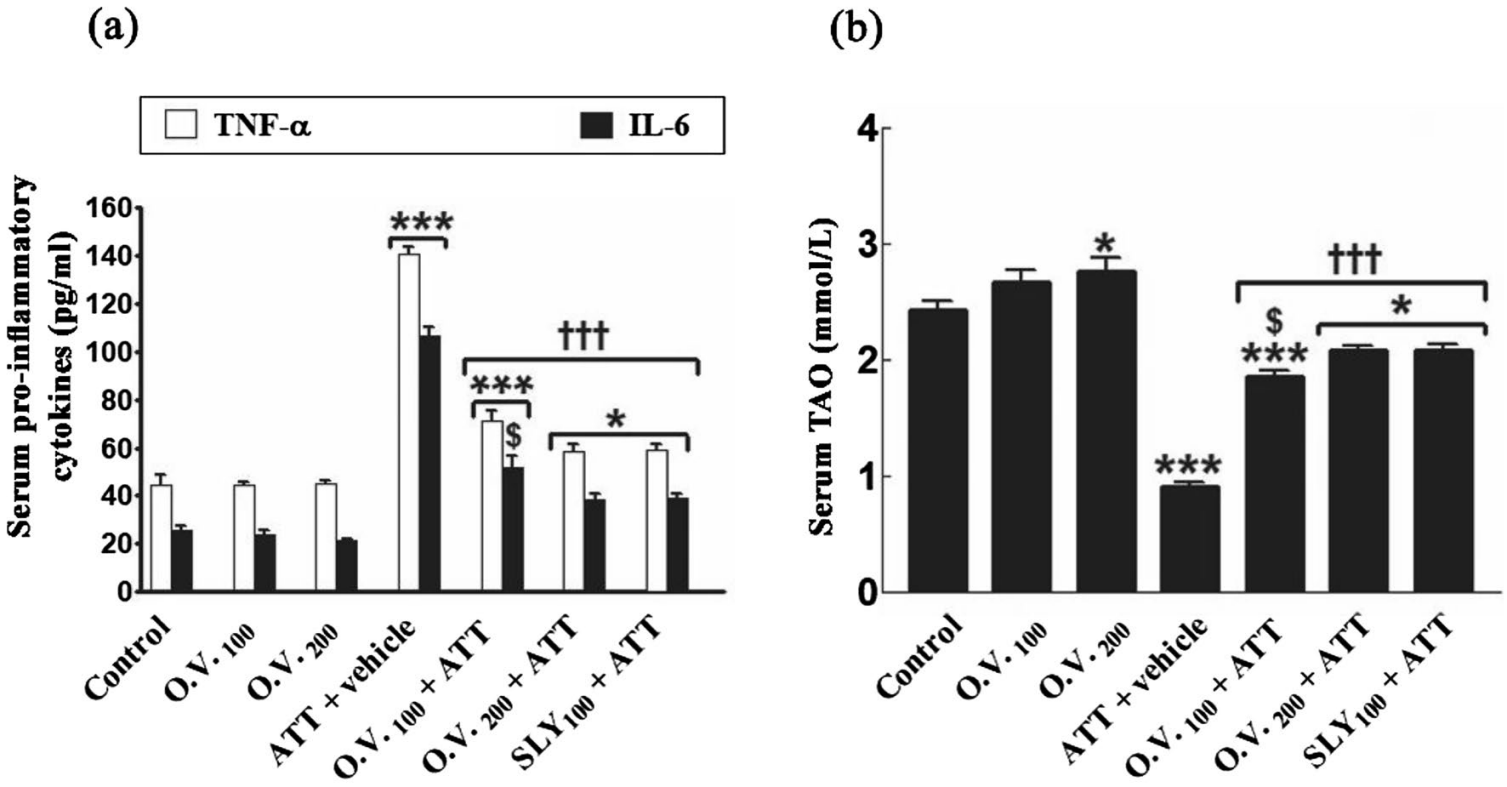
The modulatory effects of *O. vulgare* on serum pro-inflammatory cytokines (**a**) and total antioxidant capacity (**b**) in the HE-induced rat model. SEM represented by vertical bars. IL-6: interleukin-6, O.V*.*: *Origanum vulgare*, SLY: silymarin, TNF-α: tumor necrosis factor-alpha, TAO: total antioxidant. *p < 0.05; ***p < 0.001 (vs. the negative control group). †††*p* < 0.001 (vs. the HE positive control group, which received vehicle). $ *p* < 0.05 (vs. the HE group, which received silymarin).

As depicted in Fig. 6, serum proinflammatory cytokines (TNF-α and IL-6) levels and TAO capacity were significantly increased and decreased (0.05–0.001), respectively in TAA alone (TNF-α: 215%, IL-6: 320%, and TAO: − 63%, respectively), *O. vulgare* 100 + TAA (TNF-α: 59%, IL-6: 103%, and TAO: − 23%, respectively), *O. vulgare* 200 + TAA (TNF-α: 31%, IL-6: 51%, and TAO: − 14%, respectively), and SILY + TAA-treated groups (3 TNF-α: 2%, IL-6: 53%, and TAO: − 14%, respectively) compared with the control group. Otherwise, administration of *O. vulgare* or SILY significantly alleviated serum pro-inflammatory markers changes and modulate TAO capacity (*p* < 0.001) compared with the TAA alone -treated group.

### The modulatory effects of *O.vulgare* on oxidant/non-antioxidant markers in the HE-induced rat model

**Figure 7:**
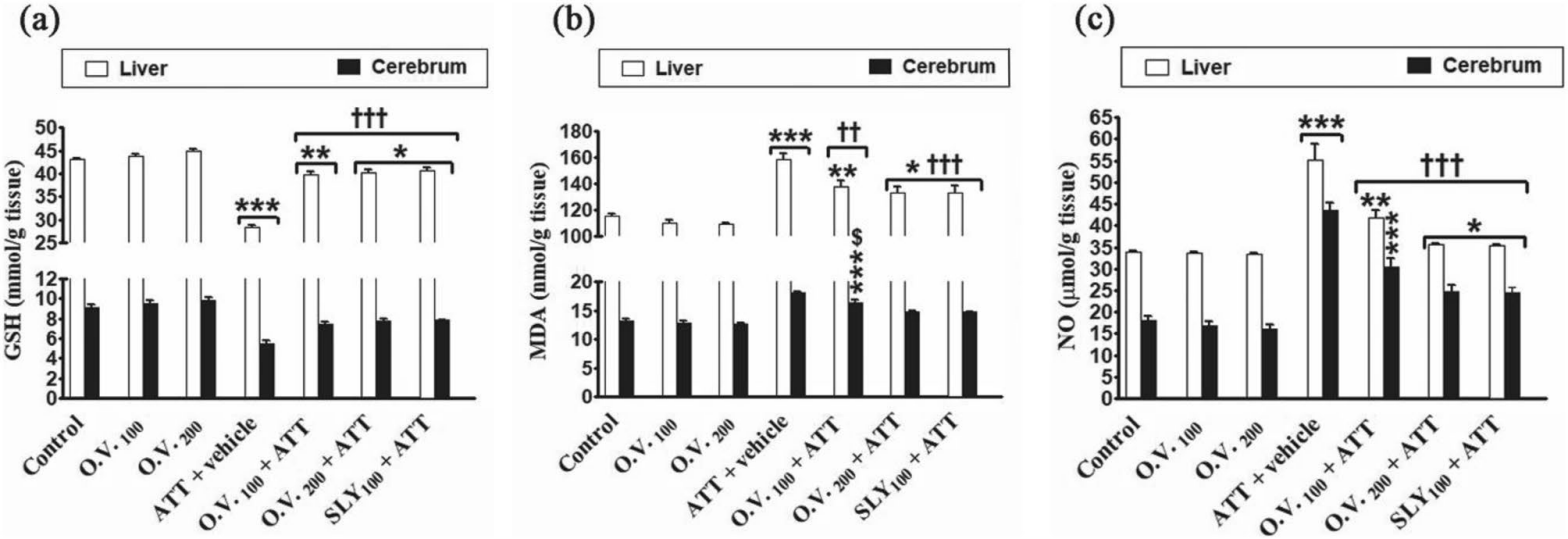
The modulatory effects of *O. vulgare* on hepatic and cerebrum GSH (**a**), MDA (**b**) and NO (**c**) in the HE-induced rat model. SEM represented by vertical bars. GSH: Reduced glutathione, MDA: Malondialdehyde, NO: Nitric oxide, O.V*.*: *Origanum vulgare*, SLY: silymarin. *p < 0.05; **p < 0.01; ***p < 0.001 (vs. the negative control group).††*p* < 0.01; †††*p* < 0.001 (vs. the HE positive control group, which received vehicle).

The results presented in Fig. 7 revealed that MDA and NO in liver and cerebrum tissues were significantly increased (*p* < 0.05–0.001) in TAA alone (38% and 37% for MDA and 19% and 140% for NO, respectively), *O. vulgare* 100 + TAA (19% and 23% for MDA and 6% and 67% for NO, respectively), *O. vulgare* 200 + TAA (15% and 12% for MDA and 5% and 37%, for NO respectively) and SILY + TAA-treated groups (16% and 11% for MDA and 4% and 35%, for NO, respectively), while GSH in liver and cerebrum tissues was significantly decreased (*p* < 0.05–0.001) in TAA alone (− 34% and − 40%, respectively), *O. vulgare* 100 + TAA (− 8% and − 18%, respectively), *O. vulgare* 200 + TAA (− 6% and − 15%, respectively) and SILY + TAA-treated groups (− 5% and − 14%, respectively) compared with the control group. Moreover, pre-treatment with *O. vulgare* (100 and 200 mg/kg) or SILY significantly decreased (*p* < 0.01–0.001) MDA and NO in liver and cerebrum tissues and significantly increased (*p* < 0.001) GSH in liver and cerebrum tissues as compared to TAA alone-treated group.

### The modulatory effects of *O.vulgare* on cerebrum antioxidant enzymes in the HE-induced rat model

**Figure 8:**
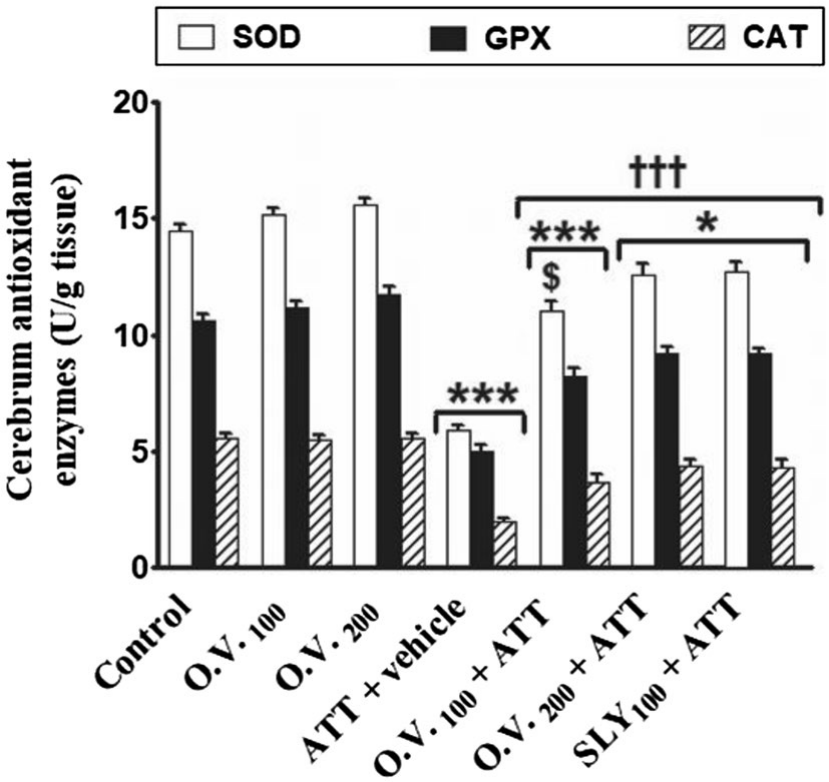
The modulatory effects of *O. vulgare* on *cerebrum antioxidant enzymes* in the HE-induced rat model. SEM represented by vertical bars. CAT: catalase, GPX: glutathione peroxidase, O.V*.*: *Origanum vulgare*, SLY: silymarin, SOD: superoxide dismutase. *p < 0.05; ***p < 0.001 (vs. the negative control group). †††*p* < 0.001 (vs. the HE positive control group, which received vehicle). $ *p* < 0.05 (vs. the HE group, which received silymarin).

The cerebrum antioxidant enzymes SOD (superoxide dismutase), GPX (glutathione peroxidase), and CAT (catalase) activities were significantly decreased (*p* < 0.05–0.001) in TAA alone (− 59%, − 53%, and − 64%, respectively), *O. vulgare* 100 + TAA (− 24%, − 23% and − 34%, respectively), *O. vulgare* 200 + TAA (− 13%, − 13%, and − 22%, respectively) and SILY + TAA-treated groups (− 12%, − 13%, and − 23%, respectively) compared with the control group (Fig. 8). On the other hand, pre-treatment with *O. vulgare* (100 and 200 mg/kg) or SILY significantly increased (*p* < 0.001) SOD, GPX and CAT in cerebrum tissue as compared to TAA alone -treated group.

### The modulatory effects of *O.vulgare* on monoaminergic neurotransmitters level in the HE-induced rat model

**Figure 9:**
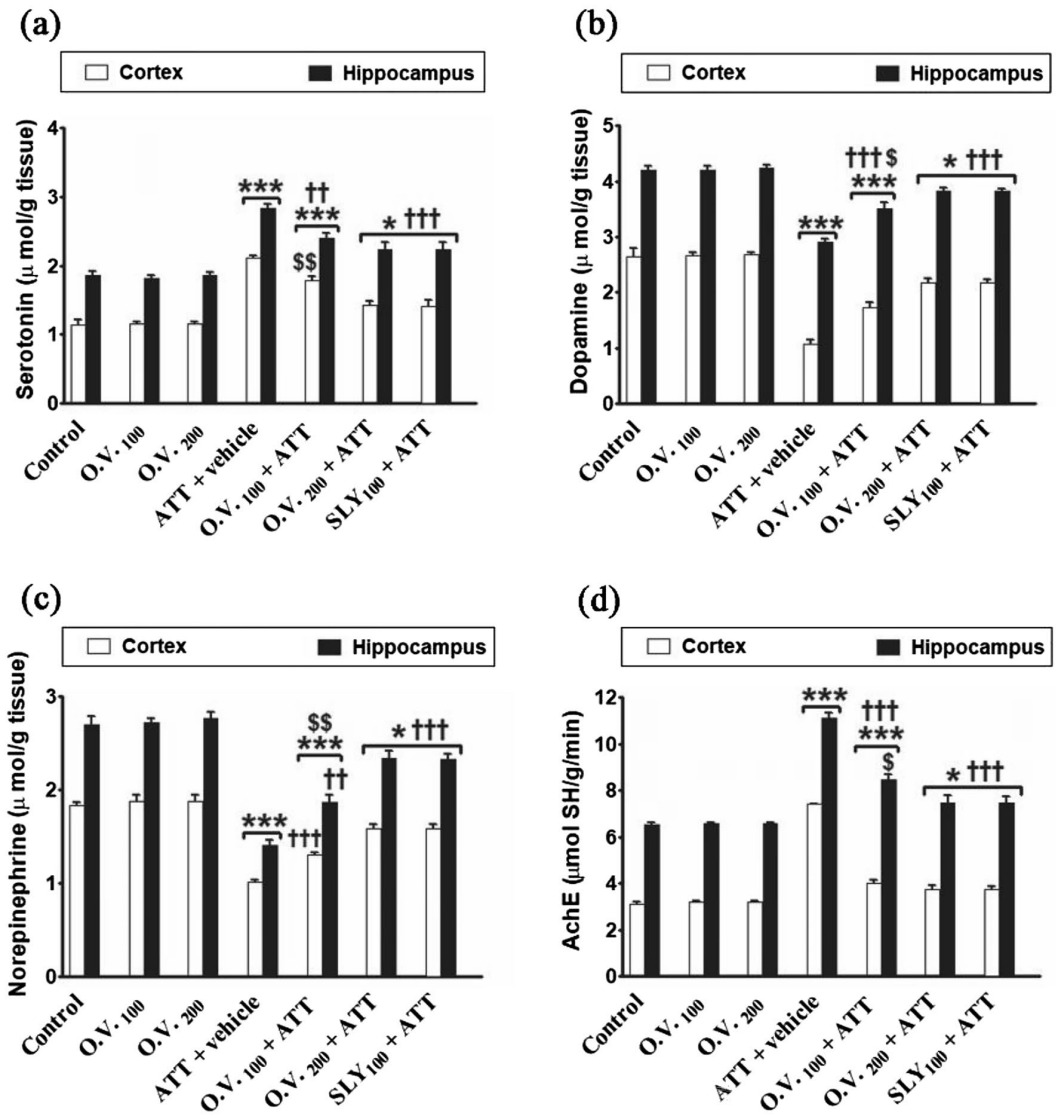
The modulatory effects of *O. vulgare* on cerebrum and hippocampus serotonin (**a**), dopamine (**b**), norepinephrine (**c**) and AchE (**d**) levels in the HE-induced rat model. SEM represented by vertical bars. AchE: Acetylcholinesterase, O.V*.*: *Origanum vulgare*, SLY: silymarin. *p < 0.05; ***p < 0.001 (vs. the negative control group). †*p* < 0.05; ††*p* < 0.01; †††*p* < 0.001 (vs. the HE positive control group, which received vehicle). $ *p* < 0.05; $$ *p* < 0.01 (vs. the HE group, which received silymarin).

Data in Fig. 9 revealed that dopamine (DA), and norepinephrine (NE) levels in cerebrum and hippocampus were significantly decreased (*p* < 0.05–0.001) in TAA alone (− 60% & − 30% for DA, and − 44% & − 48% for NE, respectively), *O. vulgare* 100 + TAA (− 34% & − 17% for DA, and − 29% & − 31% for NE, respectively), *O. vulgare* 200 + TAA (− 17% & − 9% for DA, and − 13% & − 13% for NE, respectively) and SILY + TAA-treated groups (− 18% & − 9% for DA, and − 13% & − 14% for NE, respectively), while serotonin (SE), and AchE level in cerebrum and hippocampus were significantly increased (*p* < 0.05–0.001) in TAA alone (86% & 42% for SE and 137% & 70% for AchE, respectively), *O. vulgare* 100 + TAA (56% & 29% for SE and 28% & 29% for AchE, respectively), *O. vulgare* 200 + TAA (25% & 20% for SE and 20% & 14% for AchE, respectively) and SILY + TAA-treated groups (24% & 20% for SE and 19% & 14% for AchE, respectively) compared with the control group. Further, pre-treatment with *O. vulgare* (100 and 200 mg/kg) or SILY significantly attenuated (*p* < 0.001–0.001) the changes in serotonin, dopamine, norepinephrine, and AchE levels in cerebrum and hippocampus compared to TAA alone-treated group.

### Safety and/or adverse effects caused by *O.vulgare* extract consumption in healthy normal animals

In comparison to healthy control rats, all the above parameters measured in this study were not significantly changed (*p* > 0.05) in healthy animals given either 100 or 200 mg/kg b.w. of *O. vulgare* (Table 2 and Figs. 2, 3, 4, 5, 6, 7, 8). On the other hand, only the high dose of *O. vulgare* significantly increased (*p* < 0.05) serum TAO capacity (14%) compared with the control group (Fig. 5b). Furthermore, all groups treated with *O. vulgare* had nil mortality. As a result, there were no negative effects associated with the doses of *O. vulgare* utilized in this investigation and the good health condition of the healthy rats that received *O. vulgare* continued, as indicated by the results of the behavioral and biochemical analyses.

## Discussion

Oregano spice is one of the most important culinary herbs worldwide. Several studies have reported on its broad-spectrum biological properties. Its antioxidant effect made it of use as meat preservative^35^. Oregano-derived essential oil displayed significant antibacterial properties especially against the highly pathogenic MRSA (methicillin-resistant *Staphylococcus aureus*)^36^. These properties made Oregano of particular value not only in food industry but also as a medicinal agent. Studies showed that the ethyl acetate and the ethanol extracts of oregano were rich in flavanones, flavones, and organic acids^37^, which were responsible for its documented antioxidant activity. Oregano extract as ointment was used in healing of wounds after surgery^38^, in atherosclerosis, and weight reduction^39^. Studies showed that the extract could inhibit the reuptake and the degradation of the monoamine neurotransmitters which therefore enhance the mood and reduce depression^40^. Here in our study, we aimed to investigate the potential anti-depressant and anti-anxiolytic activities of the non-polar *n*-hexane extract of *O. vulgare* and to analyze its effects on rats with HE.

TAA-induced HE showed severe liver damage associated with neurological alterations such as depressive-like behavior by increasing immobility time and reducing struggling and swimming time in FST^41^. TAA has likewise induced anxiety-like behaviors resulted in decreasing the time spent and number of entries in an open arm while increasing the time spent and number of entries in closed arm in EPM^42^. It showed significant impairment in locomotor activity and exploratory of novel environment by decreasing ambulation, grooming, and rearing frequencies concomitant with increasing the latency and freezing times in OFT^33^.

These behavioral changes were in accordance with previous studies which hinted at the induction of anxiety-like behaviors and decrease in locomotor activity and exploration in rats treated with TAA^41,43,44^, which could possibly be related to the increase in ammonia levels in the blood of HE rats model^44–46^. The increase in ammonia levels might lead to anxious behavior. Andrade and his co-workers reported that the increased passive behavior responses (immobility time) and decreased active behaviors (swimming or struggling time) in FST were indicative to depressive disorders^47^.

TAA-induced neurotoxicity affected the neurotransmitters levels. In this study, we observed that brain serotonin concentration was increased^41,48,49^, accompanied by a decrease in brain dopamine^44,50,51^ and noradrenaline^52,53^ in TAA-treated rats. These findings were in accordance with those reported earlier attributing the decrease in cognitive and locomotor activities of TAA-treated rats to the increased serotoninergic and decreased dopaminergic activities in the brains of TAA-treated rats^49,53^. Therefore, our findings supported the “false neurotransmitter hypothesis”. AchE level increased in rats administrated TAA which was similar to data reported by García-Ayllón and his co-authors^6^ on liver cirrhotic patients having an increased AchE activity in the brain^6^. Similarly, AboZaid and his co-authors showed that TAA administration caused a significant increase in brain AchE in rats^8^.

Serotonin (5-hydroxytryptamine: 5-HT) plays a critical role in the pathophysiology of mood disorders such as anxiety, depression, appetite, as well as cognitive functions like learning and memory. Serotonin, dopamine, and noradrenaline have been implicated as factors contributing to the pathogenesis of HE^50^. The changes observed in these neurotransmitters may be attributed to disturbances in the synthesis and degradation of their aromatic acids. Normally, the metabolic sequence for the formation serotonin is tryptophan-** > **5-hydroxytryptophan-** > **5-hydroxytryptamine (5-HT, serotonin)-** > **5-hydroxyindoleacetic acid as the main metabolite of serotonin.

Hyperammonemia has meanwhile several neurotoxic effects. It can alter the transfer of amino acids across neurons and can impair amino acid metabolism in the brain^41^ leading to an increased brain uptake of aromatic acids such as tryptophan, phenylalanine, and tyrosine^54^. The increased influx of tryptophan into the brain in HE could lead to an increased production of serotonin^50^. In support of this, results of previous studies demonstrated that brain serotonin and 5-hydroxyindoleacetic acid (its metabolite) concentrations were increased in animal models of HE^44,49,55^. These findings suggested that brain serotonin turnover is increased in HE^56^, which could contribute to the development of coma in the patient with liver failure. Moreover, the increased intracellular oxidation of serotonin by monoamine oxidase to 5-hydroxyindoleacetic acid could result in a potential serotoninergic synaptic deficit in brain that could be related to the early neuropsychiatric disturbances characteristic of HE like defects in cognitive, emotional, behavioral, psychomotor, and locomotive functions^57,58^. Michalak et al.^48^ stated that loss of serotonin transporter ([^3^H]-citalopram) binding sites was accompanied by significant increase in the brain extracellular fluid concentration of L-tryptophan, serotonin, and its metabolite. On the other hand, Munoz-Castaneda et al.^59^ found a significant elevation of serotonin level as a response to protect cells against oxidative damage in the brain tissue.

Dopamine and noradrenaline are important excitatory neurotransmitters, and the loss of these compounds could depress the neural activity characteristic of HE^12,60,61^. Furthermore, it was found that the brain noradrenaline concentrations were depleted in acute hepatic coma^62^. Normally, the metabolic sequence for the formation of catecholamines is phenylalanine → tyrosine (with the aid of tyrosine hydroxylase) → dopa → dopamine (with the aid of dopamine-β-hydroxylase) → norepinephrine, respectively. In liver failure associated with HE, the monoamine precursors rise several-folds in the brain^63,64^ and undergo further transformations, not only on the key physiological routes leading to the catecholamines but also on the false neurotransmitter pathways.

Fischer and Baldessarini^64^ have proposed that HE could result from the inhibition of catecholamine synthesis due to the high concentrations of phenylalanine which may inhibit the synthesis of dopa from tyrosine by competing with tyrosine (normal substrate) for the enzyme tyrosine hydroxylase and the resultant excess tyrosine is preferentially decarboxylated to form tyramine. Tyramine then competes with dopamine (normal substrate) for the enzyme dopamine-β-hydroxylase, resulting in its conversion to octopamine. Thus, the formation of both dopamine and norepinephrine in the brain is decreased and the formation of octopamine is increased.

The false neurotransmitter hypothesis proposes that octopamine is taken up and released by neurons which normally store noradrenalin and dopamine. This eventually leads to the depletion of normal neurotransmitters and the substitution of false neurotransmitters, which are incapable of appropriate synaptic activity^63,65,66^. Therefore, decreased brain dopamine may be secondary to increased dopamine turnover. On the other hand, we observed that TAA induced hyperbilirubinemia which is toxic to the central nervous system and may lead to a sequential neurological symptom known as “bilirubin encephalopathy”^67^. This hyperbilirubinemia may be attributed to decreased conjugation/secretion from the liver or blockage of bile ducts and/or impairment of bilirubin metabolism/excretion^10,68^.

TAA injection resulted in a decline in body weight gain, could be due to increased protein catabolism or loss of the animal appetite (Anorexia), while liver relative weight was increased after TAA administration^49,69,70^. A previous study showed that serotonin concentration and the increased transport of tryptophan into the brain has been associated with appetite loss in liver cirrhosis^49^. Beside these behavioral alterations, we observed that increased ammonia blood levels might contribute to an increase in the systemic pro-inflammatory cytokines^7,8,71,72^ and a decrease in the serum/brain antioxidant defense system as well as an elevation in the lipid peroxidation and oxidative stress in both the liver and the brain^4,7,28,52,73^, leading to brain edema associated with HE ^34,52,53,74–76^. The elevation of serum TNF-α that occurs during inflammation stimulates glial cells to induce an inflammatory-oxidative cascade leading to cognitive deficit^77^. Moreover, TNF-α also increases the diffusion of ammonia into astrocytes^77^ through circumventricular organs that lack a blood brain barrier to secrete cytokines^7^. According to Chu et al.^71^, the plasma levels of TNF-*α* in rats with TAA-induced fulminant hepatic failure should be significantly associated with more blunted motor activity.

Normally, ammonia is converted into urea and glutamine in the liver, and into glutamine in skeletal muscles and the brain. Hyperammonemia is caused by the reduced hepatic synthesis of urea and glutamate by which the normal liver removes ammonia from the portal blood^44,78^. In liver failure, hyperammonemia affects the mitochondrial function resulting in reduction of ATP synthesis and enhances free radical generation^73,75,79^. Blood ammonia crosses the blood brain barrier and enters in brain astrocytes. They are the only cells in the brain that can metabolize ammonia by glutamine synthetase to glutamine (osmolyte). Elevation of the intracellular levels of glutamine within the astrocytes may contribute to moving of water inside the astrocytes causing astrocyte swelling and cytotoxic brain edema^7,74,76^. Moreover, one of the mechanisms of free radical generation in tissues is mediated via autoxidation of catecholamines that have been implicated in the loss of dopaminergic neurons^80^*.*

Hyperammonemia also reduced the intracellular levels of GSH leading to an oxidative stress^81^. Ammonia likewise inhibits cystine uptake into cells^82^. As the cellular uptake of cystine is critical for GSH synthesis, the reduction in GSH levels would place astrocytes at risk for oxidative damage. In addition, ammonia toxicity increases NO production through stimulating NO synthase, that contributes to increased ROS/RNS production^4^. NO is a free radical and can react with other radicals e.g., superoxide to generate peroxynitrite, which cause oxidative changes to macromolecules. Elevated NO level occurs in neuroinflammatory states and can result in neurodegeneration. Consequently, this leads to oxidative stress which eventually results in increased levels of lipid peroxidation products (MDA) and decreased levels of antioxidants in TAA-treated rats^44,78,83^, resulting in loss of functional integrity of cell membrane and leakage of liver enzymes (ALT, AST) from cells which is an indicator of cellular liver damage^9,10,33,68^. Moreover, we observed that the significant hypercholesterolemia and hypoproteinemia may be attributed to impaired lipid and protein metabolism arising from acute liver injury induced by TAA^9,10^.

After understanding the pathological aspects of HE, we observed that *O. vulgare* hexane extract improved the motor disturbances in OFT and ameliorated the progression of depression/anxiogenic effects in FST and motor/cognitive deficits in OFT/ EPM of neurotoxic TAA. The extract displayed remarkable protective effects against the cerebral and hepatic oxidative and inflammatory pathways (triggered by TAA injection) that play a key role in HE pathogenesis. Ciulla et al.^84^ reported that the efficacy of antidepressant drugs appears in their ability to reduce immobility time and increase activity in FST. So, it might be concluded that the anxiolytics and antidepressant effects of *O. vulgare* were mediated by augmentation of noradrenergic and dopaminergic activity associated with reduction of serotoninergic activity in HE rat model. Additionally, *O. vulgare* hexane extract effectively mitigated brain edema and hyperammonemia in HE model and significantly alleviated serum toxicity markers, oxidative/nitrative stress, lipid peroxidation, and inflammatory biomarkers concomitant with an elevation in their antioxidant levels which were more obvious at the highest doses of the extract. These prominent effects of the *n*-hexane extract could be attributed to its major metabolites including sterols (37%), tocopherols (18%), triterpenoids (15%), fatty acid methyl esters FAME (5%), and oxygenated monoterpenes (4%). Sterols, tocopherols, and triterpenes have been reported to reduce the ethanol-induced hepatic oxidative stress^85^. The former class of metabolites include cholesten-3-one, β-sitosterol, campesterol, β-amyrin, α-amyrin, and stigmasterol, which are characterized by the presence of unsaturated pi-electrons and hydroxy function, which act as the main trapping systems for reactive oxygen species. Similarly, FAME among them the saturated palmitic and arachidic acids as well as the polyunsaturated linolenic acid contribute to the total antioxidant effect of the *n*-hexane extract through the carbonyl pi-electrons and the alkene system. Tocopherols like α and γ forms are isomers of vitamin E and they represent the second major class of metabolites in Organum *n*-hexane extract. They are strong fat-soluble antioxidants due to the conjugation in their phenyl ring. Their potency even exceeds that of β- carotene and ascorbic acid in alleviating hepatic oxidative stress^85^. E-squalene is a linear triterpene with six unsaturated bonds which makes the compound of high value as a free radical scavenger. In a similar way, lupeol and betulin are pentacyclic triterpenes with one double bond and a hydroxy function. These bioactive constituents act as scavengers for reactive oxygen species, stabilizing the cell membrane therefore preventing their oxidative damage and further lipid peroxidation. These results were in agreement with previous studies showing that the concentration of the total phenolics were higher in the hexane extract compared to water, dichloromethane, and methanol extract^86^. To the best of our knowledge, this is the first study to investigate the efficacy of *O. vulgare* n-hexane extract against TAA-induced HE and its associated biochemical alterations.

Another interesting finding of the present study was that no mortality or harmful occurred in the healthy groups received *O. vulgare* only. During the test period, biochemical examinations revealed no abnormalities in any of these groups. Also, there were no notable treatment-related changes in behavior activity. Oregano also increased serum total antioxidant activity in a similar way to silymarin. These effects were attributed to the presence of antioxidant polyphenols and flavonoids^87–89^, which confirmed the findings of the current investigation. Thus, the use of Oregano as dietary supplementation in food/spice industry appears to be safe based on the lack of toxicity and improve the antioxidant status during the present study especially at high dose (200 mg/kg b.w).

## Conclusion

Several mechanisms are involved in the pathogenesis of HE, including hyperammonemia, oxidative stress, inflammation, brain edema, and variations in neurotransmitters level. These mechanisms trigger neuro-biochemical and behavioral alterations. *O. vulgare* might be a useful agent to prevent dramatic deterioration of liver function, inhibit the sharp rise in blood and brain ammonia in case of acute and chronic liver injury, relieve symptoms of inflammation, enhance antioxidant status, and reduce the severity of neurological/behavioral symptoms. Also, the hepatoprotective/neuro-protective activities of *O. vulgare* was found to be equivalent to that of SILY in ATT-induced HE rat model, suggesting that consuming enough doses of Oregano may be useful in mitigating HE progression.

## Supplementary Information

Supplementary Information.

## Data Availability

The datasets used and/or analysed during the current study available from the corresponding author on reasonable request.

## Abbreviations

5-HT: 5-Hydroxytryptamine
AchE: Acetylcholinesterase
ALT: Alanine aminotransferase
AST: Aspartate aminotransferase
DA: Dopamine
ELISA: Enzyme-linked immunosorbent assay
EPMT: Elevated plus-maze test
FAMEs: Fatty acid methyl esters
FST: Forced swimming test
GC/MS: Gas Chromatography/Mass Spectrometry
GSH: Glutathione
HE: Hepatic encephalopathy
IL-6: Interleukin-6
MDA: Malondialdehyde
NE: Norepinephrine
NO: Nitric oxide
OFT: Open-field test
RI: Kovats retention index
ROS: Reactive oxygen species
SILY: Silymarin
SOD: Superoxide dismutase
TAA: Thioacetamide
TNF-α: Tumor necrosis factor-alpha
