# Supplementary material for: Ameliorative effect of oregano (Origanum vulgare) versus silymarin in experimentally induced hepatic encephalopathy
Source: Sci Rep. 2022 Oct 25;12:17854. doi: 10.1038/s41598-022-20412-3 (PMC9596437; doi:10.1038/s41598-022-20412-3)

Ain Shams University
Faculty of Pharmacy
GC-MS

Sample Information

Analyzed by	: Admin
Analyzed	: 6/5/2017 11:34:35 AM
Sample Type	: Unknown
Level #	: 1
Sample Name	: MAJ
Sample ID	: 001
IS Amount	: [1]=1
Sample Amount	: 1
Dilution Factor	: 1
Vial #	: 1
Injection Volume	: 1.00
Data File	: C:\GCMSsolution\Data\7102015\MAJ_001_652017_1.qgd
Org Data File	: C:\GCMSsolution\Data\7102015\MAJ_001_652017_1.qgd
Method File	: C:\GCMSsolution\Data\Project1\Hexane - IA.qgm
Org Method File	: C:\GCMSsolution\Data\Project1\Hexane - IA.qgm
Report File	:
Tuning File	: C:\GCMSsolution\System\Tune1_default.qgt
Modified by	: Admin
Modified	: 6/5/2017 12:37:36 PM

Chromatogram MAJ C:\GCMSsolution\Data\7102015\MAJ_001_652017_1.qgd

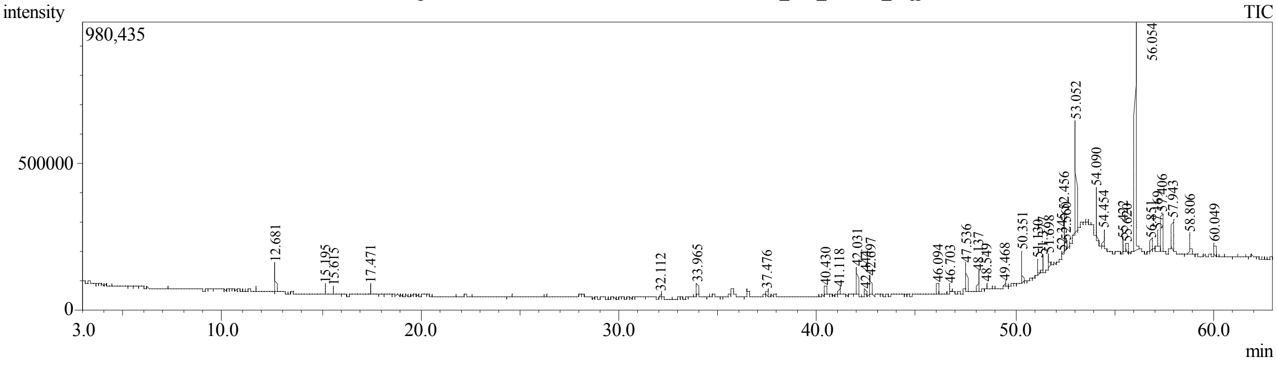

Peak Report TIC

Peak#	R.Time	Area	Area%	Name	Base m/z
1	12.681	240737	2.18	Bicyclo[3.1.0]hexan-2-ol, 2-methyl-5-(1-methylethyl)-, (1.alpha.,2.alpha.,5.alpha.)-	93.05
2	15.195	78896	0.71	Bicyclo[3.1.0]hexan-2-ol, 2-methyl-5-(1-methylethyl)-, (1.alpha.,2.beta.,5.alpha.)-	93.10
3	15.615	60396	0.55	.alpha.-Terpineol	93.05
4	17.471	115090	1.04	Cyclohexene, 1-methyl-4-(1-methylethylidene)-	93.05
5	32.112	39484	0.36	3,7,11,15-Tetramethyl-2-hexadecen-1-ol	43.05
6	33.965	92772	0.84	Pentadecanoic acid, 14-methyl-, methyl ester	74.05
7	37.476	39316	0.36	9,12,15-Octadecatrienoic acid, methyl ester, (Z,Z,Z)-	79.05
8	40.430	91936	0.83	Sulfurous acid, cyclohexylmethyl hexyl ester	97.10
9	41.118	62215	0.56	2,4,4-Trimethyl-1-pentyl methylphosphonofluoridate	57.10
10	42.031	267335	2.42	Sulfurous acid, cyclohexylmethyl heptyl ester	97.10
11	42.444	42489	0.38		159.15
12	42.697	179193	1.62	6-Undecen-3-one, 5-butyl-2,2-dimethyl-, (E)-	57.10
13	46.094	89415	0.81	Sulfurous acid, cyclohexylmethyl heptyl ester	97.10
14	46.703	80188	0.73	Nonane, 2-bromo-5-ethyl-	57.10
15	47.536	262193	2.37	Sulfurous acid, cyclohexylmethyl hexyl ester	97.10
16	48.137	194034	1.76	3-Heptene, 2,2,4,6,6-pentamethyl-	57.10
17	48.549	55382	0.50	Tetratetracontane	57.10
18	49.468	53200	0.48	2,6,10,14,18,22-Tetracosahexaene, 2,6,10,15,19,23-hexamethyl-, (all-E)-	69.05
19	50.351	277992	2.51	Tetratetracontane	57.10
20	51.130	172448	1.56	Sulfurous acid, cyclohexylmethyl pentadecyl ester	97.10
21	51.377	163601	1.48	Heptacosane	57.10
22	51.698	158005	1.43	Nonane, 2-bromo-5-ethyl-	57.10
23	52.345	45741	0.41	Carbonic acid, isobutyl octadecyl ester	57.10
24	52.456	437586	3.96	Sulfurous acid, cyclohexylmethyl octadecyl ester	97.10
25	52.560	81359	0.74	Hexatriacontane	57.10
26	53.052	1495133	13.52	Tetratriacontane	57.10
27	54.090	553595	5.01	Tetratetracontane	57.10
28	54.454	178991	1.62	Tetratriacontane	57.10
29	55.422	144161	1.30	Tetratetracontane	57.10
30	55.620	86794	0.79	Tetratetracontane	57.10
31	56.054	3018173	27.30	Tetratetracontane	57.10
32	56.851	162686	1.47	Nonane, 2-bromo-5-ethyl-	97.10
33	57.169	366323	3.31	.beta.-Sitosterol	43.05
34	57.406	579304	5.24	Tetratetracontane	57.10

Peak#	R.Time	Area	Area%	Name	Base m/z
35	57.943	540599	4.89	Sulfurous acid, cyclohexylmethyl octadecyl ester	97.10
36	58.806	348619	3.15	3-Heptene, 2,2,4,6,6-pentamethyl-	57.10
37	60.049	200609	1.81	Tetratriacontane	57.10
		11055990	100.00		

Library

<< Target >>
Line#:1 R.Time:12.680(Scan#:1937) Retention Index:1100 MassPeaks:304
RawMode:Averaged 12.675-12.685(1936-1938) BasePeak:93.05(12595)
BG Mode:Calc. from Peak Group 1 - Event 1 Scan

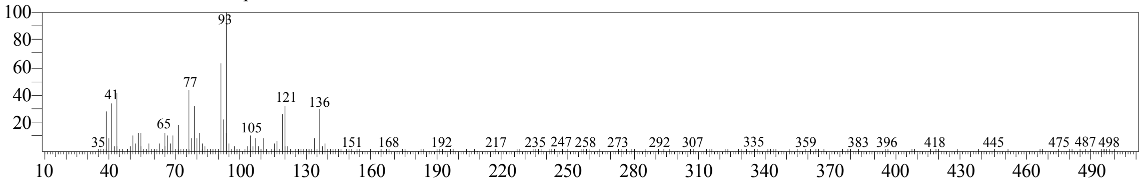

Hit#:1 Entry:10004 Library:NIST11s.lib
SI:89 Formula:C10H18O CAS:17699-16-0 MolWeight:154 RetIndex:1041
CompName:Bicyclo[3.1.0]hexan-2-ol, 2-methyl-5-(1-methylethyl)-, (1.alpha.,2.alpha.,5.alpha.)- $$ trans-Sabinene hydrate (trans for Me vs IP) $$ trans-Sabi

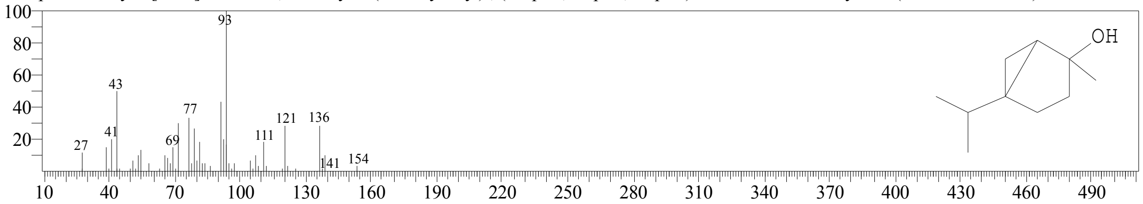

Hit#:2 Entry:9822 Library:NIST11.lib
SI:87 Formula:C10H16 CAS:99805-90-0 MolWeight:136 RetIndex:1023
CompName:Cyclohexene, 4-methyl-3-(1-methylethylidene)- $$ 4-Methyl-3-(1-methylethylidene)-1-cyclohexene # $$

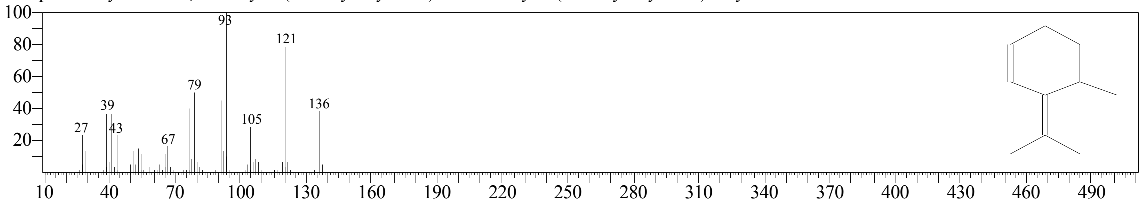

Hit#:3 Entry:9824 Library:NIST11.lib
SI:87 Formula:C10H16 CAS:586-62-9 MolWeight:136 RetIndex:1052
CompName:Cyclohexene, 1-methyl-4-(1-methylethylidene)- $$ p-Mentha-1,4(8)-diene $$ Terpinolene $$ Terpinolen $$ UN 2541 $$ .alpha.- Terpinolen $$

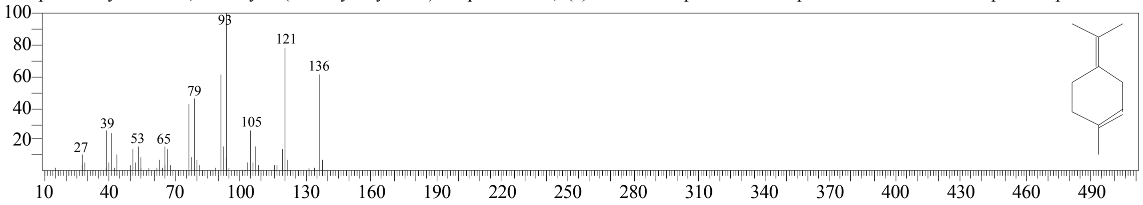

Hit#:4 Entry:6676 Library:NIST11s.lib
SI:87 Formula:C10H16 CAS:554-61-0 MolWeight:136 RetIndex:948
CompName:2-Carene $$ Bicyclo[4.1.0]hept-2-ene, 3,7,7-trimethyl- $$ .delta.-2-Carene $$ (.+/-.)-2-Carene $$ 3,7,7-Trimethylbicyclo[4.1.0]hept-2-ene $$

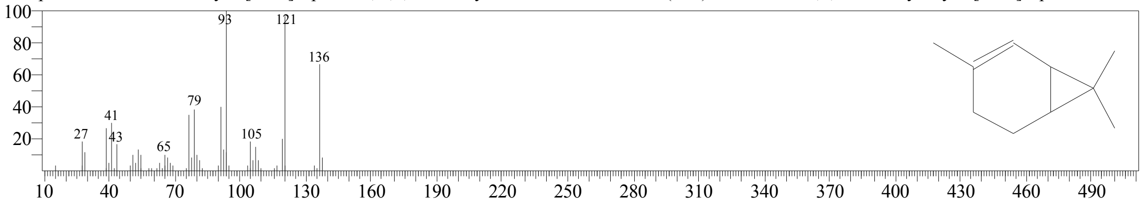

Hit#:5 Entry:6308 Library:NIST05s.LIB
SI:86 Formula:C10H16 CAS:99-85-4 MolWeight:136 RetIndex:998
CompName:1,4-Cyclohexadiene, 1-methyl-4-(1-methylethyl)- $$ .gamma.-Terpinen $$ .gamma.-Terpinene $$ p-Mentha-1,4-diene $$ Crithmene $$ Moslene

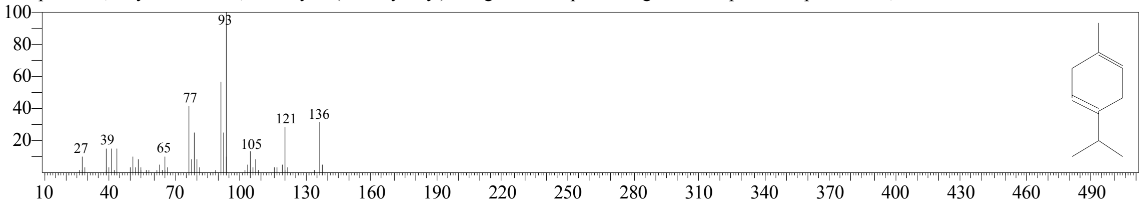

<< Target >>
Line#:2 R.Time:15.195(Scan#:2440) Retention Index:1180 MassPeaks:260
RawMode:Averaged 15.190-15.200(2439-2441) BasePeak:93.10(3177)
BG Mode:Calc. from Peak Group 1 - Event 1 Scan

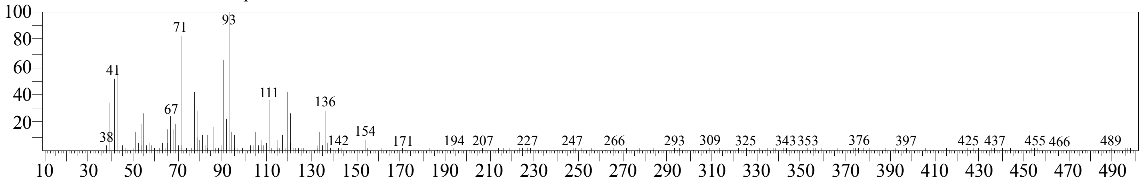

Hit#:1 Entry:16897 Library:NIST05.LIB
SI:84 Formula:C10H18O CAS:15537-55-0 MolWeight:154 RetIndex:1041
CompName:Bicyclo[3.1.0]hexan-2-ol, 2-methyl-5-(1-methylethyl)-, (1.alpha.,2.beta.,5.alpha.)- $$ 5-Isopropyl-2-methylbicyclo[3.1.0]hexan-2-ol # $$

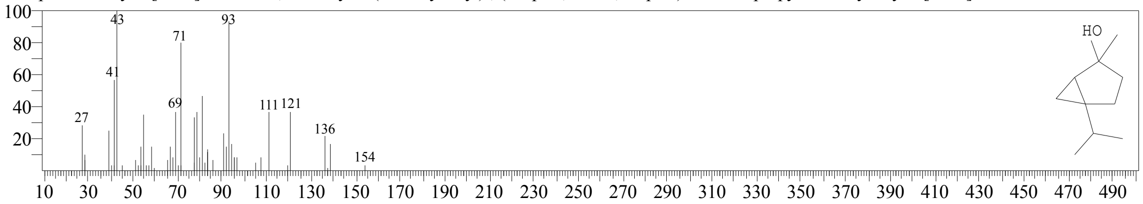

Hit#:2 Entry:10004 Library:NIST11s.lib
SI:82 Formula:C10H18O CAS:17699-16-0 MolWeight:154 RetIndex:1041
CompName:Bicyclo[3.1.0]hexan-2-ol, 2-methyl-5-(1-methylethyl)-, (1.alpha.,2.alpha.,5.alpha.)- $$ trans-Sabinene hydrate (trans for Me vs IP) $$ trans-Sabi

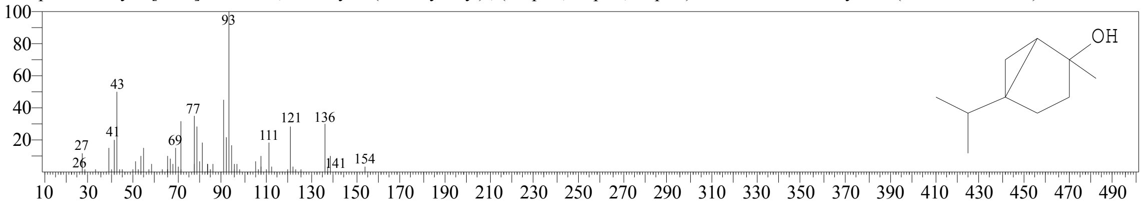

Hit#:3 Entry:9980 Library:NIST11s.lib
SI:79 Formula:C10H18O CAS:562-74-3 MolWeight:154 RetIndex:1137
CompName:Terpinen-4-ol $$ 3-Cyclohexen-1-ol, 4-methyl-1-(1-methylethyl)- $$ p-Menth-1-en-4-ol $$ 1-Terpinen-4-ol $$ 4-Carvomenthenol $$ 4-Terpinec

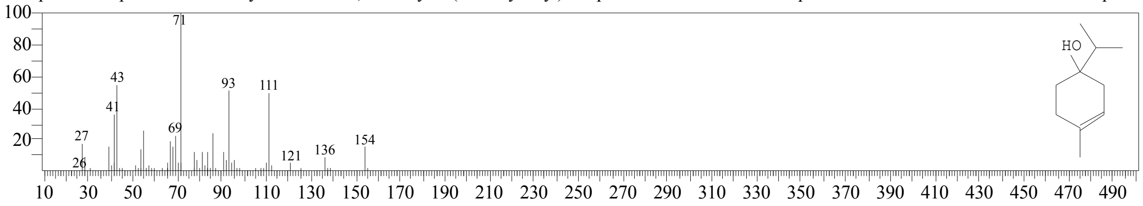

Hit#:4 Entry:9395 Library:NIST05s.LIB
SI:79 Formula:C10H18O CAS:106-25-2 MolWeight:154 RetIndex:1228
CompName:2,6-Octadien-1-ol, 3,7-dimethyl-, (Z)- $$ cis-Geraniol $$ cis-3,7-Dimethyl-2,6-octadien-1-ol $$ Nerol $$ Neryl alcohol $$ 2-cis-3,7-Dimethyl-2

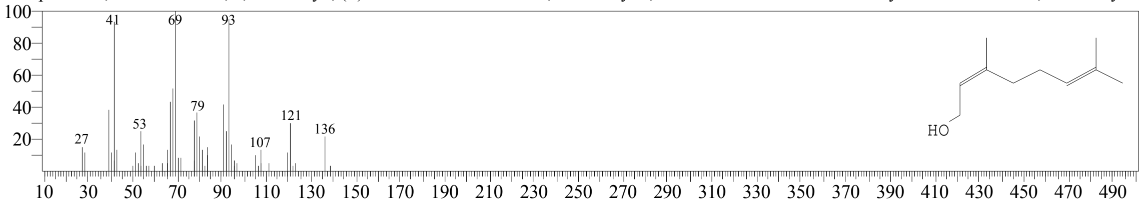

Hit#:5 Entry:17561 Library:NIST11.lib
SI:79 Formula:C10H18O CAS:79605-62-2 MolWeight:154 RetIndex:1249
CompName:1-Cyclohexyl-2-buten-1-ol (c,t) $$ (2E)-1-Cyclohexyl-2-buten-1-ol # $$

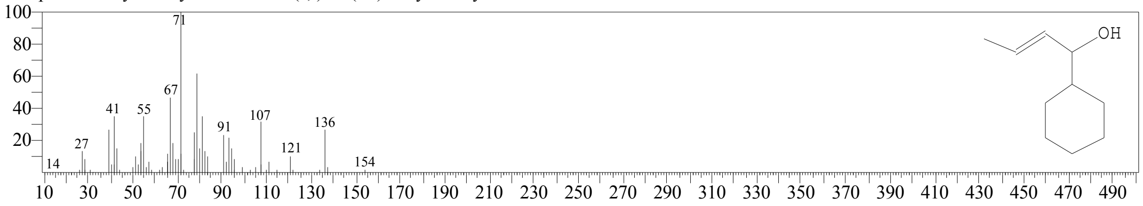

<< Target >>
Line#:3   R.Time:15.615(Scan#:2524) Retention Index:1193 MassPeaks:308
RawMode:Averaged 15.610-15.620(2523-2525) BasePeak:93.05(1999)
BG Mode:Calc. from Peak Group 1 - Event 1 Scan

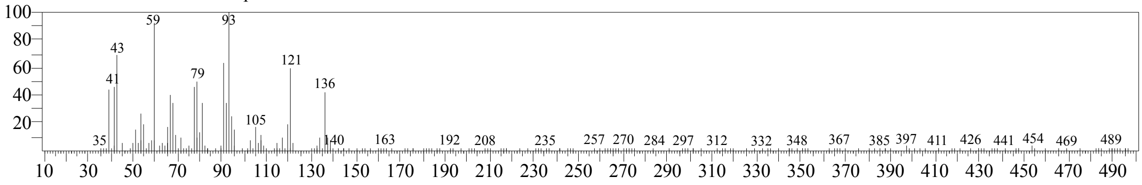

Hit#:1 Entry:17520 Library:NIST11.lib
SI:91 Formula:C10H18O CAS:98-55-5 MolWeight:154 RetIndex:1143
CompName:.alpha.-Terpineol $$ 3-Cyclohexene-1-methanol, .alpha.,.alpha.4-trimethyl- $$ p-Menth-1-en-8-ol $$ Terpineol schlechthin $$ Terpineol, .alpha.

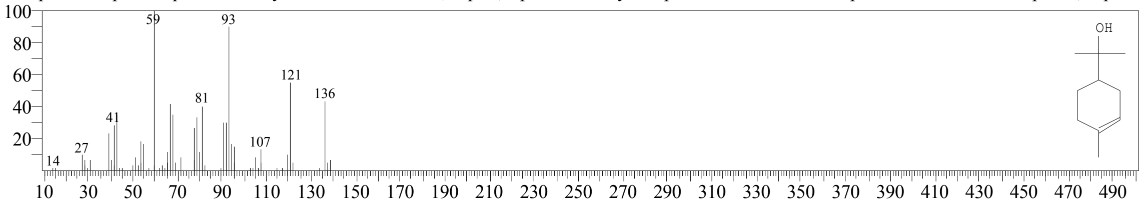

Hit#:2 Entry:9608 Library:NIST05.LIB
SI:84 Formula:C10H16 CAS:29714-87-2 MolWeight:136 RetIndex:993
CompName:Ocimene $$ Octane, 2,6-dimethyl-, hexadehydro deriv. $$ Octatriene, dimethyl- $$ Dimethyloctatriene $$ (4E,6E)-2,6-Dimethyl-2,4,6-octatrien

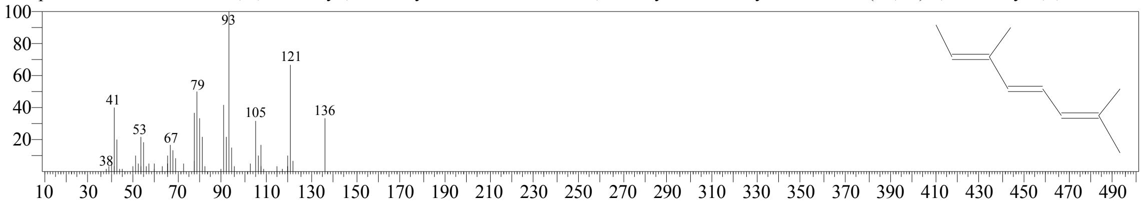

Hit#:3 Entry:9849 Library:NIST11.lib
SI:83 Formula:C10H16 CAS:24524-57-0 MolWeight:136 RetIndex:956
CompName:Bicyclo[3.1.0]hexane, 6-isopropylidene-1-methyl- $$ Bicyclo[3.1.0]hexane, 1-methyl-6-(1-methylethylidene)- $$ 1-Methyl-6-(1-methylethylide

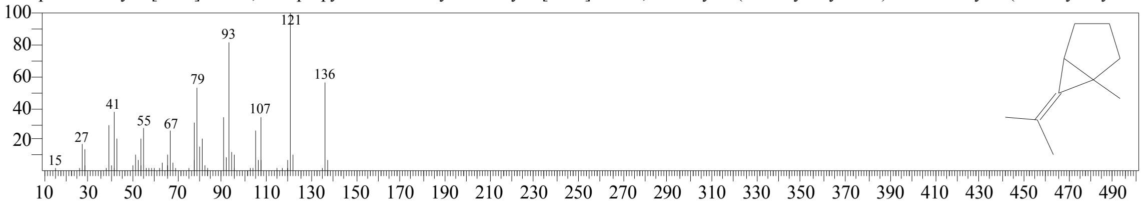

Hit#:4 Entry:9573 Library:NIST05.LIB
SI:82 Formula:C10H16 CAS:53282-47-6 MolWeight:136 RetIndex:1002
CompName:Bicyclo[4.1.0]heptane, 7-(1-methylethylidene)- $$ 7-(1-Methylethylidene)bicyclo[4.1.0]heptane # $$

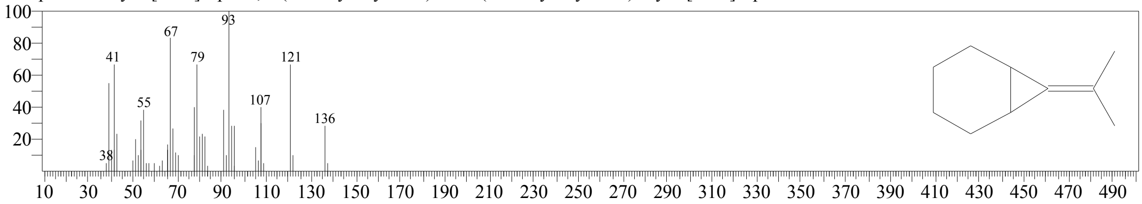

Hit#:5 Entry:6676 Library:NIST11s.lib
SI:82 Formula:C10H16 CAS:554-61-0 MolWeight:136 RetIndex:948
CompName:2-Carene $$ Bicyclo[4.1.0]hept-2-ene, 3,7,7-trimethyl- $$ .delta.-2-Carene $$ (.+/-.)-2-Carene $$ 3,7,7-Trimethylbicyclo[4.1.0]hept-2-ene $$

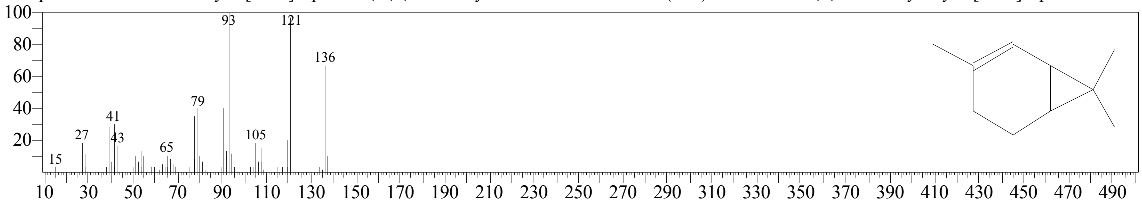

<< Target >>
Line#:4 R.Time:17.470(Scan#:2895) Retention Index:1257 MassPeaks:306
RawMode:Averaged 17.465-17.475(2894-2896) BasePeak:93.05(4878)
BG Mode:Calc. from Peak Group 1 - Event 1 Scan

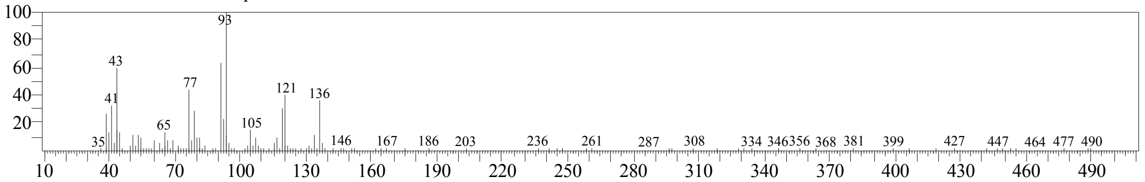

Hit#:1 Entry:9824 Library:NIST11.lib
SI:87 Formula:C10H16 CAS:586-62-9 MolWeight:136 RetIndex:1052
CompName:Cyclohexene, 1-methyl-4-(1-methylethylidene)- $$ p-Mentha-1,4(8)-diene $$ Terpinolene $$ Terpinolen $$ UN 2541 $$ .alpha.- Terpinolen $$

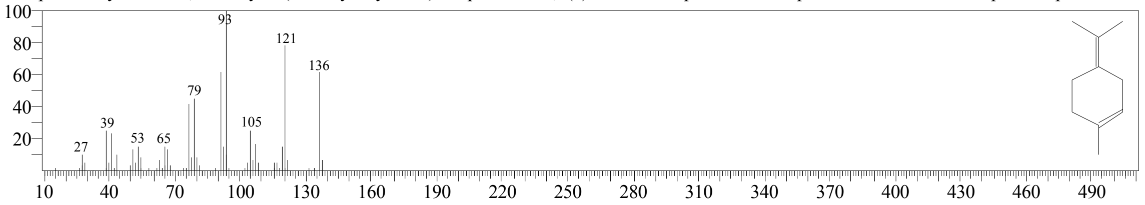

Hit#:2 Entry:41487 Library:NIST11.lib
SI:86 Formula:C12H20O2 CAS:4821-04-9 MolWeight:196 RetIndex:1327
CompName:4-Terpinenyl acetate $$ 3-Cyclohexen-1-ol, 4-methyl-1-(1-methylethyl)-, acetate $$ p-Menth-1-en-4-ol, acetate $$ Terpinene 4-acetate $$ 1-Terp

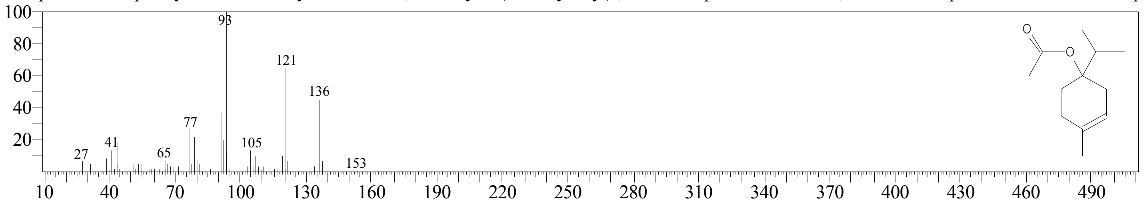

Hit#:3 Entry:6308 Library:NIST05s.LIB
SI:86 Formula:C10H16 CAS:99-85-4 MolWeight:136 RetIndex:998
CompName:1,4-Cyclohexadiene, 1-methyl-4-(1-methylethyl)- $$ .gamma.-Terpinen $$ .gamma.-Terpinene $$ p-Mentha-1,4-diene $$ Crithmene $$ Moslene

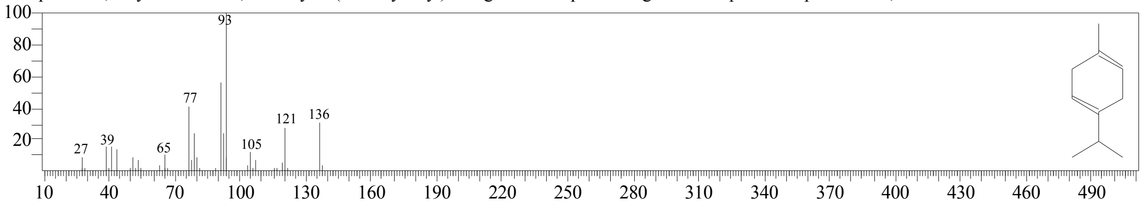

Hit#:4 Entry:6676 Library:NIST11s.lib
SI:86 Formula:C10H16 CAS:554-61-0 MolWeight:136 RetIndex:948
CompName:2-Carene $$ Bicyclo[4.1.0]hept-2-ene, 3,7,7-trimethyl- $$ .delta.-2-Carene $$ (.+/-.)-2-Carene $$ 3,7,7-Trimethylbicyclo[4.1.0]hept-2-ene $$

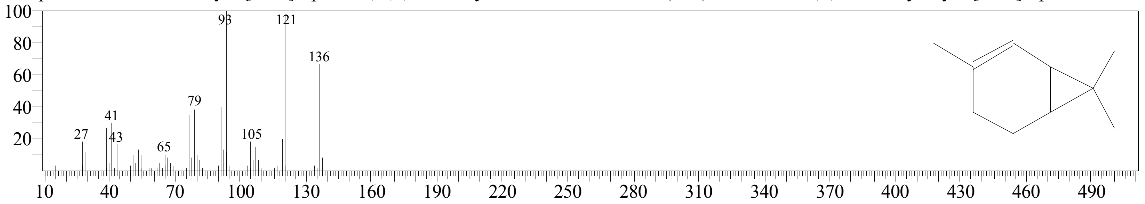

Hit#:5 Entry:9816 Library:NIST11.lib
SI:86 Formula:C10H16 CAS:0-00-0 MolWeight:136 RetIndex:948
CompName:(+)-2-Carene

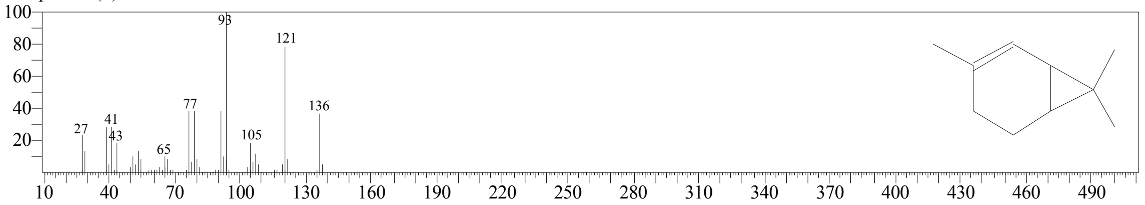

<< Target >>
Line#:5 R.Time:32.110(Scan#:5823) Retention Index:1838 MassPeaks:263
RawMode:Averaged 32.105-32.115(5822-5824) BasePeak:43.05(1442)
BG Mode:Calc. from Peak Group 1 - Event 1 Scan

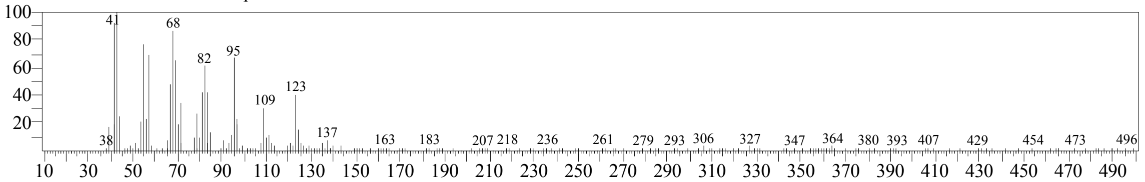

Hit#:1 Entry:115518 Library:NIST11.lib
SI:90 Formula:C20H40O CAS:102608-53-7 MolWeight:296 RetIndex:2045
CompName:3,7,11,15-Tetramethyl-2-hexadecen-1-ol $$ 2-Hexadecen-1-ol, 3,7,11,15-tetramethyl $$

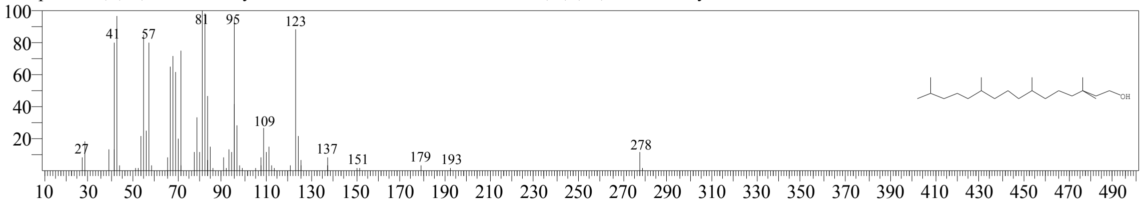

Hit#:2 Entry:147507 Library:NIST11.lib
SI:88 Formula:C22H42O2 CAS:0-00-0 MolWeight:338 RetIndex:2168
CompName:Phytol, acetate

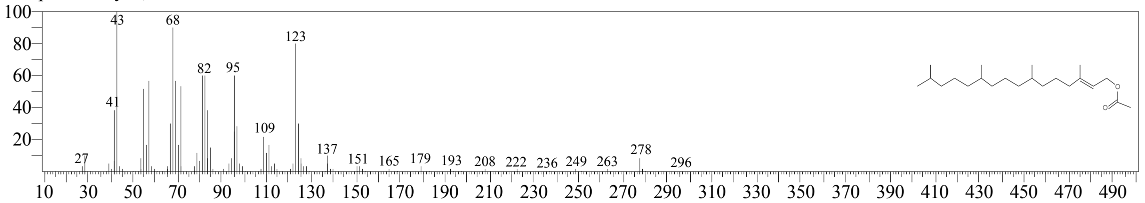

Hit#:3 Entry:20938 Library:NIST05s.LIB
SI:87 Formula:C18H34 CAS:629-89-0 MolWeight:250 RetIndex:1808
CompName:1-Octadecyne

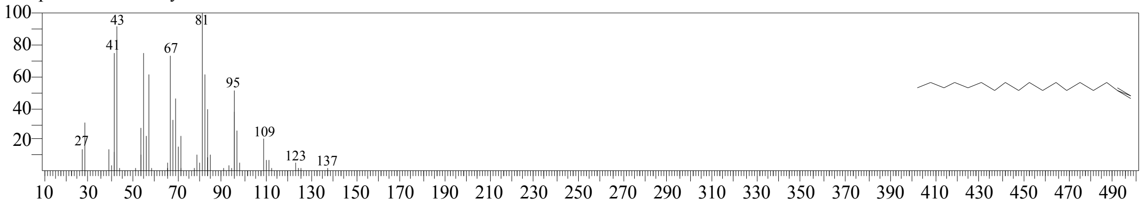

Hit#:4 Entry:22022 Library:NIST11s.lib
SI:87 Formula:C16H32O CAS:7320-37-8 MolWeight:240 RetIndex:1702
CompName:Oxirane, tetradecyl- $$ Hexadecane, 1,2-epoxy- $$ Hexadecylene oxide $$ 1,2-Epoxyhexadecane $$ 1,2-Hexadecane oxide $$ 1,2-Hexadecene

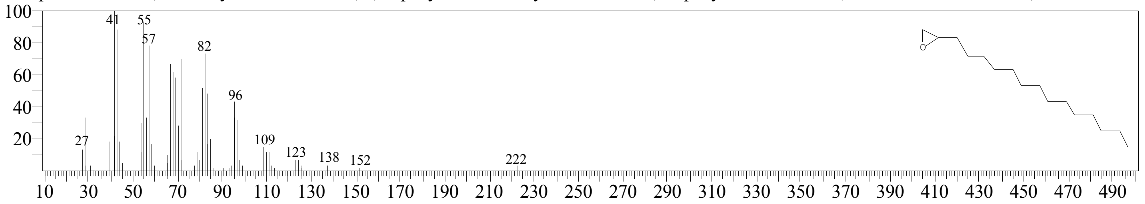

Hit#:5 Entry:19155 Library:NIST11s.lib
SI:86 Formula:C14H28O CAS:124-25-4 MolWeight:212 RetIndex:1601
CompName:Tetradecanal $$ Myristaldehyde $$ Myristylaldehyde $$ Tetradecylaldehyde $$ n-Tetradecanal $$ Aldehyde C-14 $$ Aldehyde C-14, myristic $

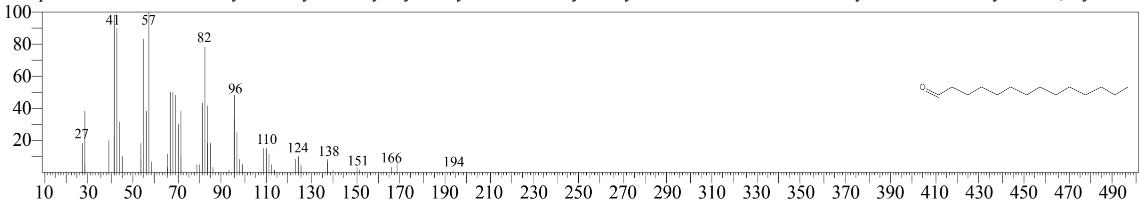

<< Target >>
Line#:6 R.Time:33.965(Scan#:6194) Retention Index:1927 MassPeaks:252
RawMode:Averaged 33.960-33.970(6193-6195) BasePeak:74.05(7523)
BG Mode:Calc. from Peak Group 1 - Event 1 Scan

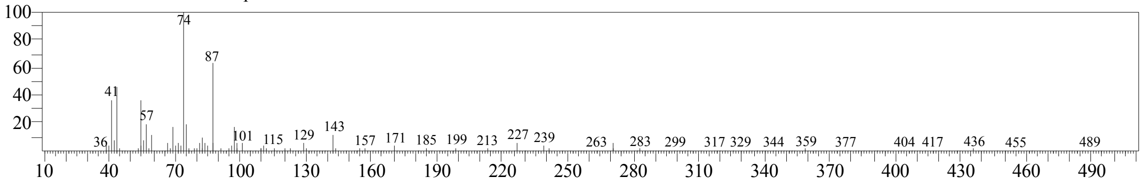

Hit#:1 Entry:22223 Library:NIST05s.LIB
SI:94 Formula:C17H34O2 CAS:5129-60-2 MolWeight:270 RetIndex:1814
CompName:Pentadecanoic acid, 14-methyl-, methyl ester $$ Methyl 14-methylpentadecanoate # $$

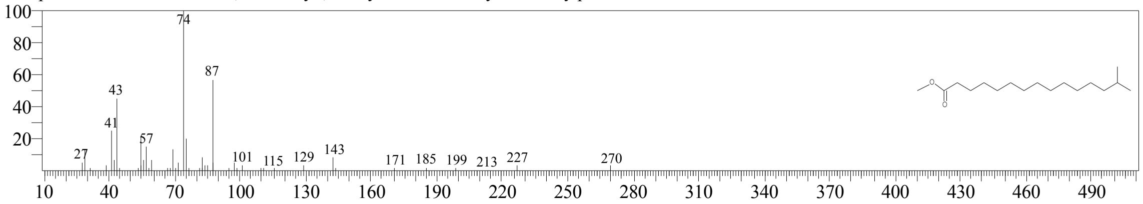

Hit#:2 Entry:24296 Library:NIST11s.lib
SI:93 Formula:C17H34O2 CAS:112-39-0 MolWeight:270 RetIndex:1878
CompName:Hexadecanoic acid, methyl ester $$ Palmitic acid, methyl ester $$ n-Hexadecanoic acid methyl ester $$ Metholene 2216 $$ Methyl hexadecano

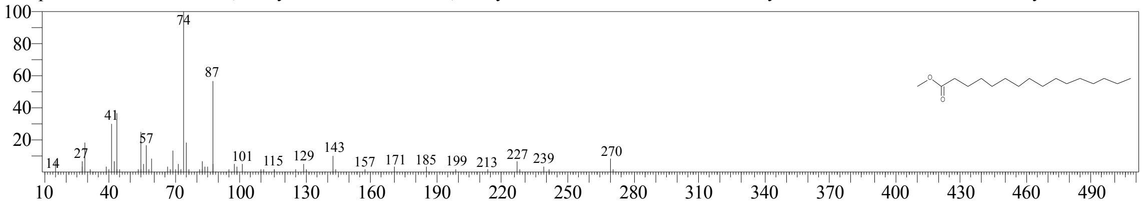

Hit#:3 Entry:22987 Library:NIST05s.LIB
SI:93 Formula:C18H36O2 CAS:6929-04-0 MolWeight:284 RetIndex:1914
CompName:Hexadecanoic acid, 15-methyl-, methyl ester $$ Methyl isoheptadecanoate $$ Methyl 15-methylhexadecanoate $$

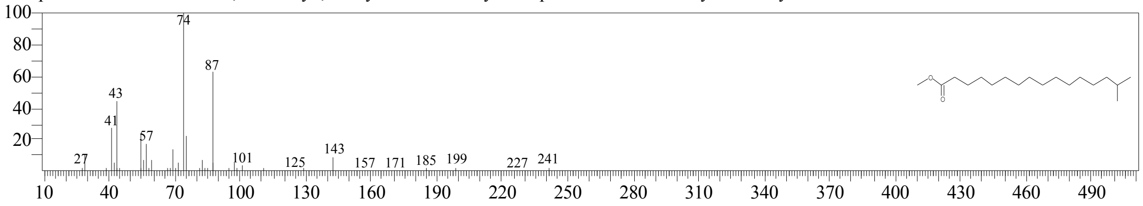

Hit#:4 Entry:73852 Library:NIST11.lib
SI:92 Formula:C15H30O2 CAS:5129-58-8 MolWeight:242 RetIndex:1615
CompName:Tridecanoic acid, 12-methyl-, methyl ester $$ Methyl isomyristate $$ Methyl 12-methyltridecanoate $$

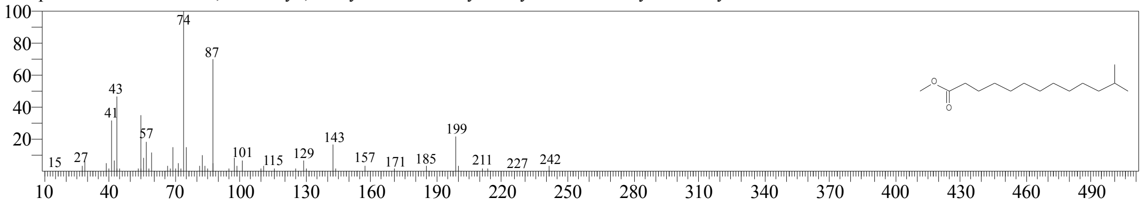

Hit#:5 Entry:20940 Library:NIST11s.lib
SI:92 Formula:C14H28O2 CAS:1731-88-0 MolWeight:228 RetIndex:1580
CompName:Tridecanoic acid, methyl ester $$ Methyl tridecanoate $$ n-Tridecanoic acid methyl ester $$ Methyl ester of tridecanoic acid $$ Methyl n-tridec

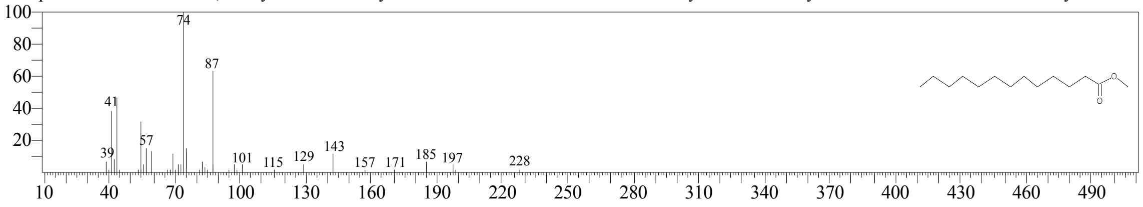

<< Target >>
Line#:7 R.Time:37.475(Scan#:6896) Retention Index:2109 MassPeaks:282
RawMode:Averaged 37.470-37.480(6895-6897) BasePeak:79.05(1893)
BG Mode:Calc. from Peak Group 1 - Event 1 Scan

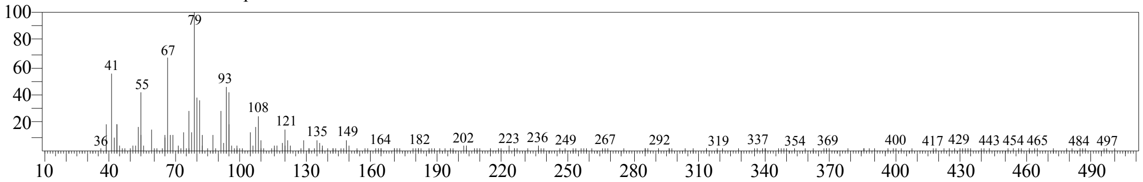

Hit#:1 Entry:25700 Library:NIST11s.lib
SI:92 Formula:C19H32O2 CAS:301-00-8 MolWeight:292 RetIndex:2101
CompName:9,12,15-Octadecatrienoic acid, methyl ester, (Z,Z,Z)- $$ Linolenic acid, methyl ester $$ Methyl all-cis-9,12,15-octadecatrienoate $$ Methyl lino

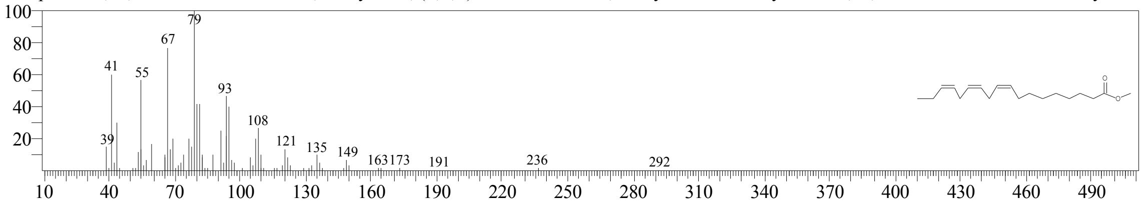

Hit#:2 Entry:27218 Library:NIST11s.lib
SI:91 Formula:C21H36O2 CAS:55682-88-7 MolWeight:320 RetIndex:2300
CompName:11,14,17-Eicosatrienoic acid, methyl ester $$ Methyl 11,14,17-icosatrienoate $$ Methyl 11,14,17-eicosatrienoate $$

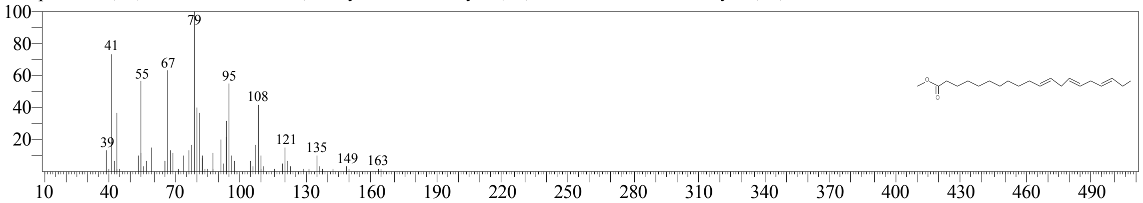

Hit#:3 Entry:79688 Library:NIST05.LIB
SI:90 Formula:C17H28O2 CAS:56554-30-4 MolWeight:264 RetIndex:1902
CompName:7,10,13-Hexadecatrienoic acid, methyl ester $$ Methyl (7E,10E,13E)-7,10,13-hexadecatrienoate # $$

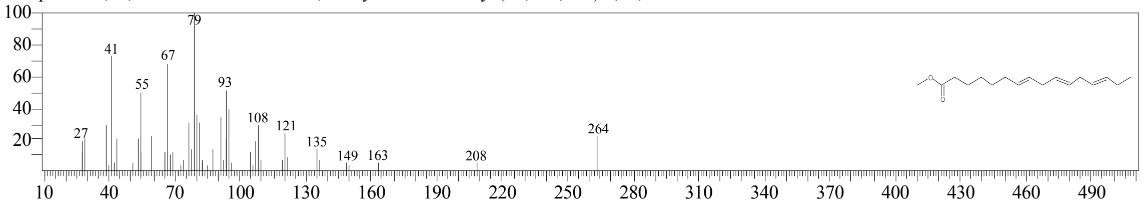

Hit#:4 Entry:101390 Library:NIST11.lib
SI:88 Formula:C18H30O2 CAS:463-40-1 MolWeight:278 RetIndex:2191
CompName:9,12,15-Octadecatrienoic acid, (Z,Z,Z)- $$ Linolenic acid $$ .alpha.-Linolenic acid $$ All-cis-9,12,15-Octadecatrienoic acid $$ cis,cis,cis-9,12,

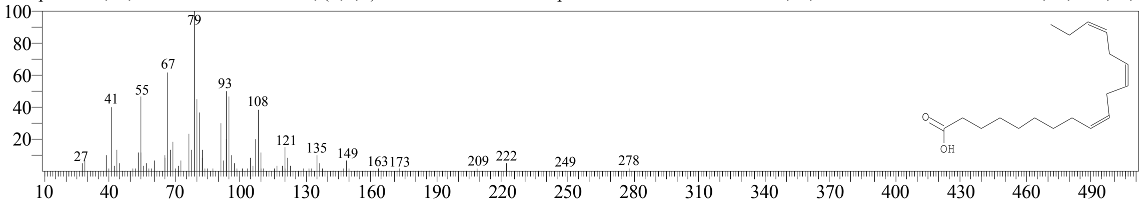

Hit#:5 Entry:90503 Library:NIST11.lib
SI:87 Formula:C18H32O CAS:506-44-5 MolWeight:264 RetIndex:2077
CompName:9,12,15-Octadecatrien-1-ol, (Z,Z,Z)- $$ (9E,12E,15E)-9,12,15-Octadecatrien-1-ol # $$ (Z,Z,Z)-9,12,15-Octadecatrien-1-ol $$

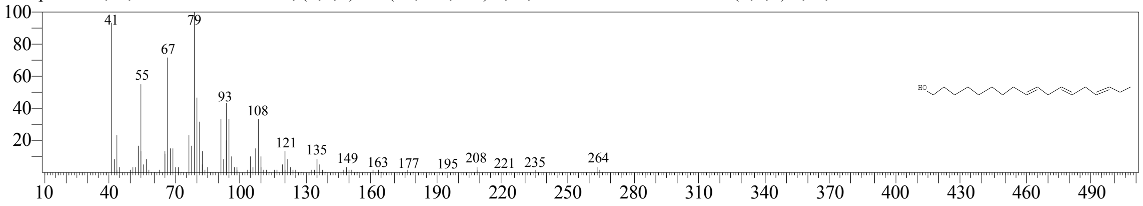

<< Target >>
Line#:8   R.Time:40.430(Scan#:7487) Retention Index:2271 MassPeaks:244
RawMode:Averaged 40.425-40.435(7486-7488) BasePeak:97.10(9420)
BG Mode:Calc. from Peak Group 1 - Event 1 Scan

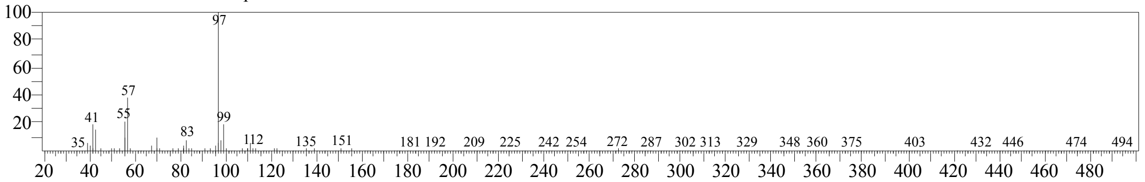

Hit#:1 Entry:78203 Library:NIST05.LIB
SI:88 Formula:C13H26O3S CAS:0-00-0 MolWeight:262 RetIndex:2000
CompName:Sulfurous acid, cyclohexylmethyl hexyl ester

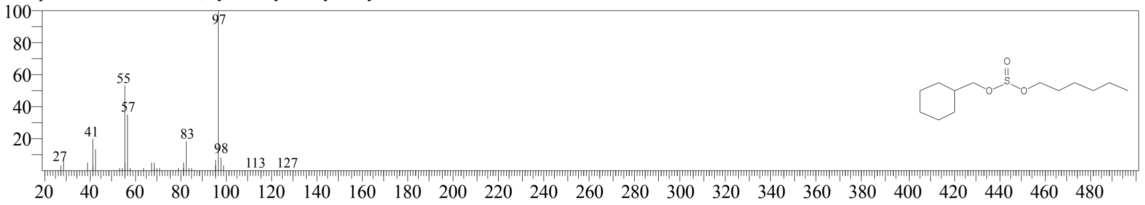

Hit#:2 Entry:86823 Library:NIST05.LIB
SI:88 Formula:C14H28O3S CAS:0-00-0 MolWeight:276 RetIndex:2100
CompName:Sulfurous acid, cyclohexylmethyl heptyl ester

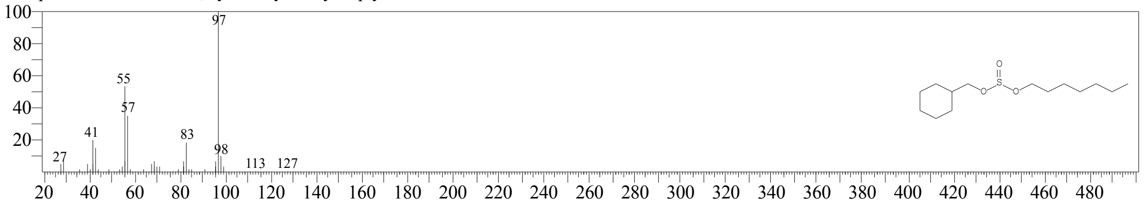

Hit#:3 Entry:118580 Library:NIST05.LIB
SI:86 Formula:C18H36O3S CAS:0-00-0 MolWeight:332 RetIndex:2497
CompName:Sulfurous acid, cyclohexylmethyl undecyl ester

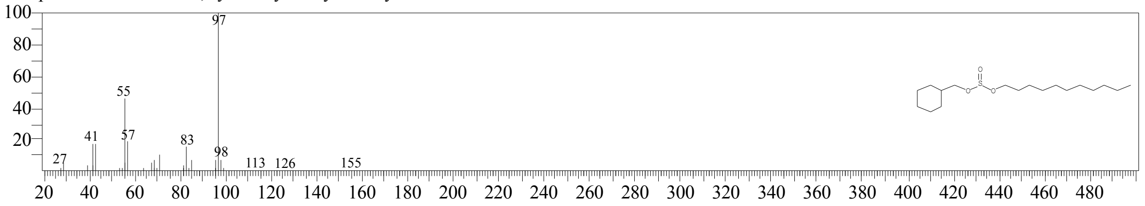

Hit#:4 Entry:125328 Library:NIST05.LIB
SI:86 Formula:C19H38O3S CAS:0-00-0 MolWeight:346 RetIndex:2597
CompName:Sulfurous acid, cyclohexylmethyl dodecyl ester

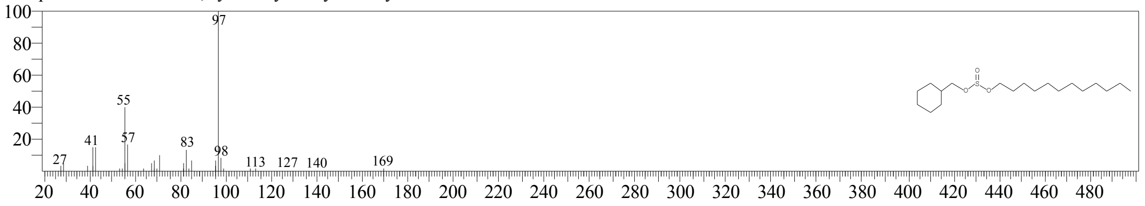

Hit#:5 Entry:150324 Library:NIST05.LIB
SI:86 Formula:C25H50O3S CAS:0-00-0 MolWeight:430 RetIndex:3193
CompName:Sulfurous acid, cyclohexylmethyl octadecyl ester

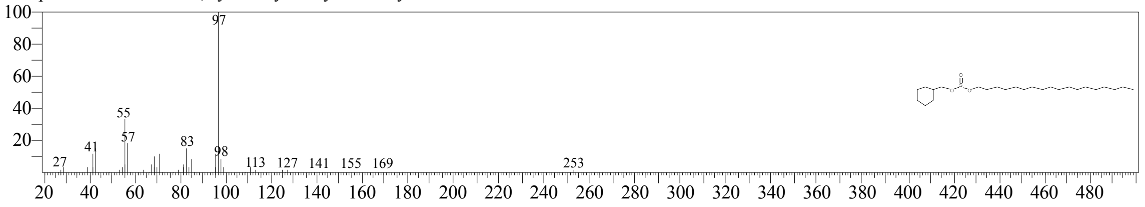

<< Target >>
Line#:9 R.Time:41.120(Scan#:7625) Retention Index:2309 MassPeaks:318
RawMode:Averaged 41.115-41.125(7624-7626) BasePeak:57.10(3339)
BG Mode:Calc. from Peak Group 1 - Event 1 Scan

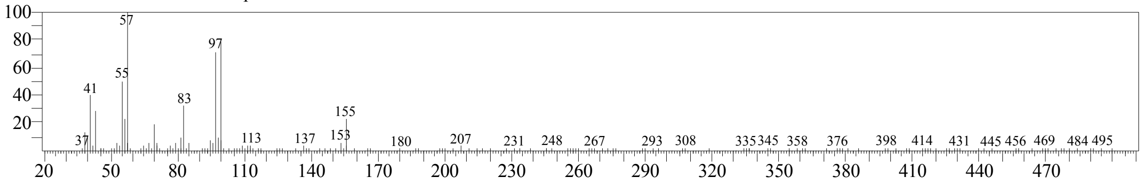

Hit#:1 Entry:17360 Library:NIST05s.LIB
SI:81 Formula:C9H20FO2P CAS:333416-06-1 MolWeight:210 RetIndex:0
CompName:2,4,4-Trimethyl-1-pentyl methylphosphonofluoridate $$ Methylphosphonic acid, fluoroanhydride, 2,3,3-trimethylpentyl ester $$ 2,4,4-Trimethy

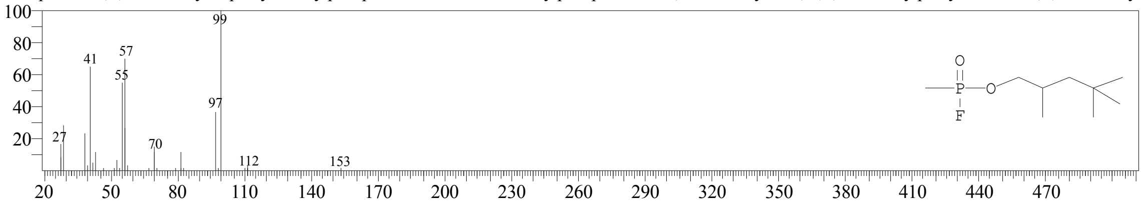

Hit#:2 Entry:23870 Library:NIST05.LIB
SI:81 Formula:C12H24 CAS:0-00-0 MolWeight:168 RetIndex:1097
CompName:1-Hexene, 2,4,4-triethyl-

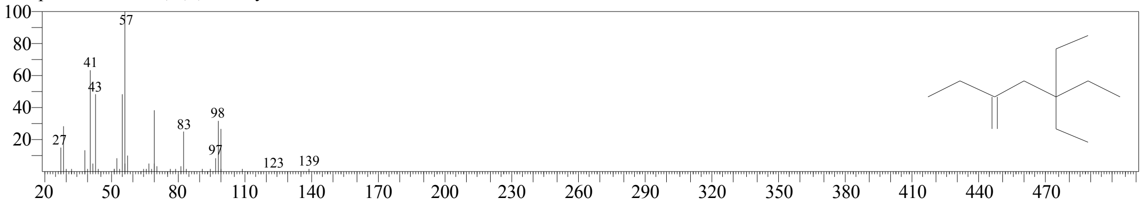

Hit#:3 Entry:61283 Library:NIST05.LIB
SI:80 Formula:C11H23Br CAS:55162-38-4 MolWeight:234 RetIndex:1283
CompName:Nonane, 2-bromo-5-ethyl- $$ 2-Bromo-5-ethylnonane # $$

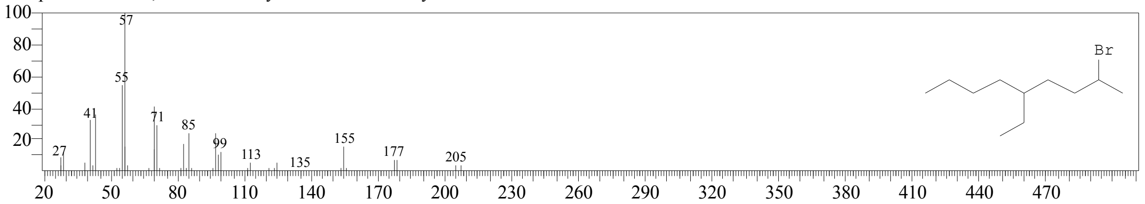

Hit#:4 Entry:8753 Library:NIST05.LIB
SI:79 Formula:C7H15Cl CAS:35951-33-8 MolWeight:134 RetIndex:726
CompName:2-Chloro-2,4-dimethylpentane

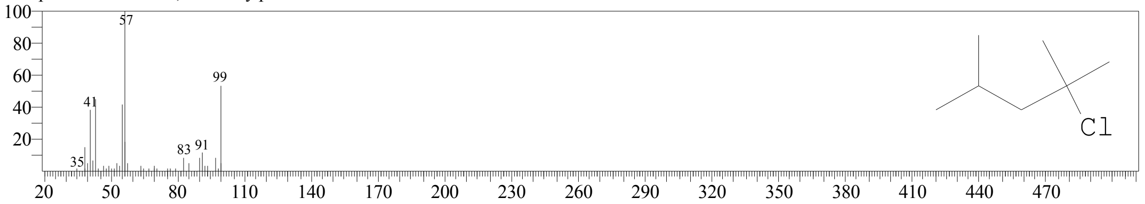

Hit#:5 Entry:17133 Library:NIST05.LIB
SI:79 Formula:C11H22 CAS:75736-66-2 MolWeight:154 RetIndex:1055
CompName:Cyclohexane, 1-(1,1-dimethylethyl)-4-methyl- $$ 1-tert-Butyl-4-methylcyclohexane # $$

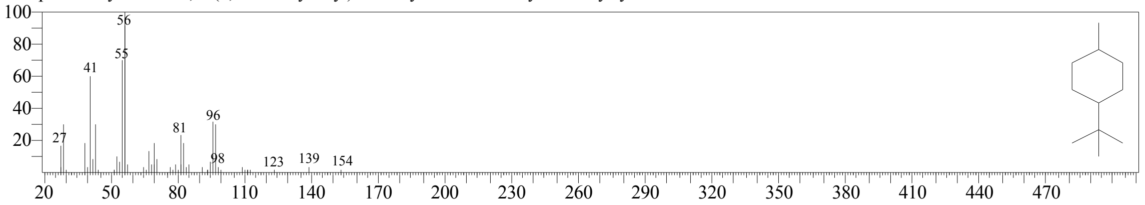

<< Target >>
Line#:10 R.Time:42.030(Scan#:7807) Retention Index:2359 MassPeaks:273
RawMode:Averaged 42.025-42.035(7806-7808) BasePeak:97.10(31164)
BG Mode:Calc. from Peak Group 1 - Event 1 Scan

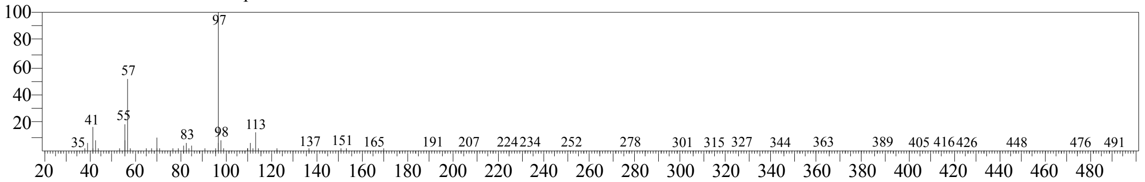

Hit#:1 Entry:99564 Library:NIST11.lib
SI:87 Formula:C14H28O3S CAS:0-00-0 MolWeight:276 RetIndex:2100
CompName:Sulfurous acid, cyclohexylmethyl heptyl ester

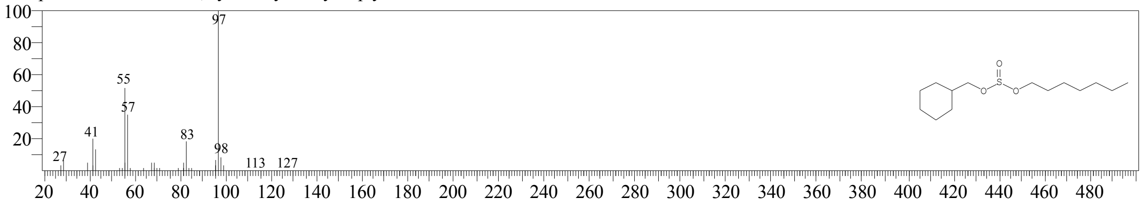

Hit#:2 Entry:88563 Library:NIST11.lib
SI:87 Formula:C13H26O3S CAS:0-00-0 MolWeight:262 RetIndex:2000
CompName:Sulfurous acid, cyclohexylmethyl hexyl ester

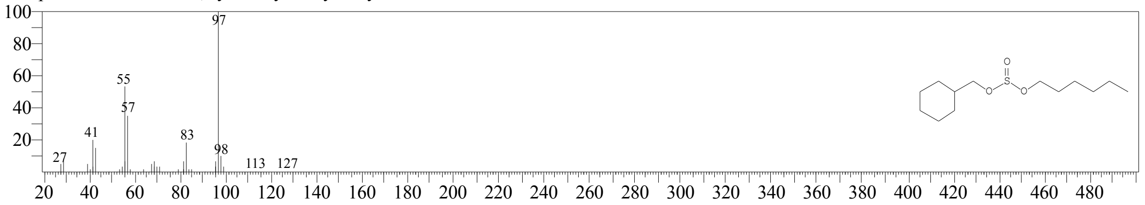

Hit#:3 Entry:86823 Library:NIST05.LIB
SI:87 Formula:C14H28O3S CAS:0-00-0 MolWeight:276 RetIndex:2100
CompName:Sulfurous acid, cyclohexylmethyl heptyl ester

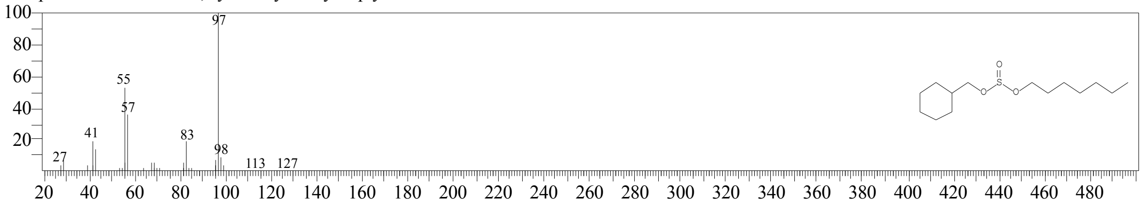

Hit#:4 Entry:78203 Library:NIST05.LIB
SI:87 Formula:C13H26O3S CAS:0-00-0 MolWeight:262 RetIndex:2000
CompName:Sulfurous acid, cyclohexylmethyl hexyl ester

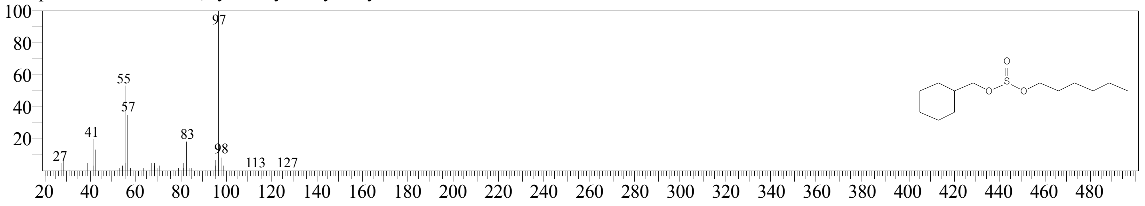

Hit#:5 Entry:61278 Library:NIST05.LIB
SI:86 Formula:C11H22O3S CAS:0-00-0 MolWeight:234 RetIndex:1737
CompName:Sulfurous acid, cyclohexylmethyl isobutyl ester

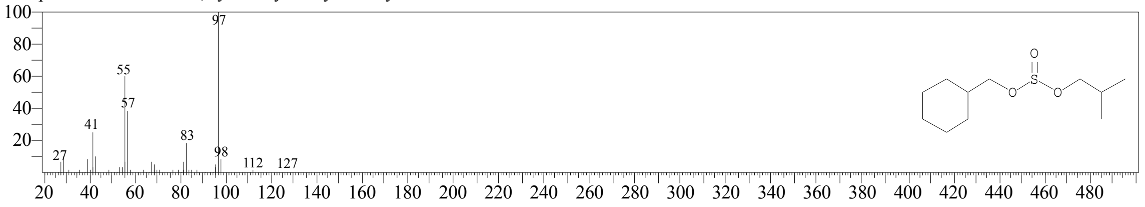

<< Target >>
Line#:11 R.Time:42.445(Scan#:7890) Retention Index:2381 MassPeaks:291
RawMode:Averaged 42.440-42.450(7889-7891) BasePeak:159.15(936)
BG Mode:Calc. from Peak Group 1 - Event 1 Scan

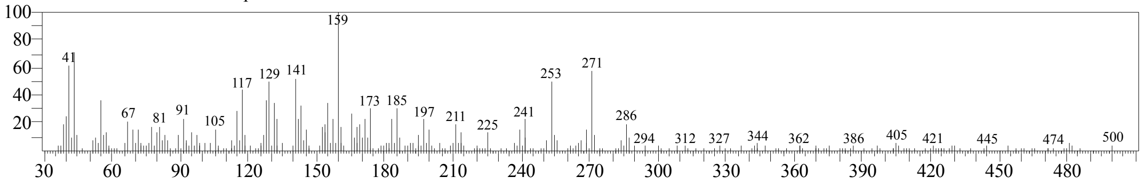

No hit compound

<< Target >>
Line#:12 R.Time:42.695(Scan#:7940) Retention Index:2395 MassPeaks:247
RawMode:Averaged 42.690-42.700(7939-7941) BasePeak:57.10(15162)
BG Mode:Calc. from Peak Group 1 - Event 1 Scan

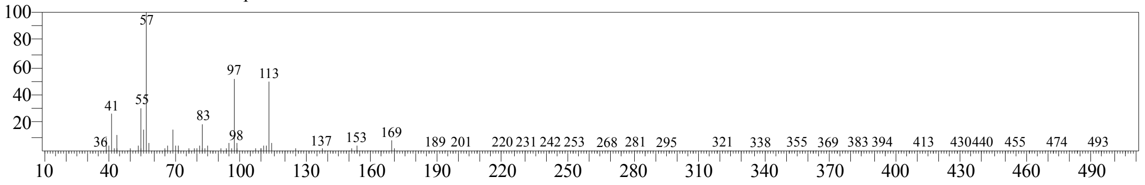

Hit#:1 Entry:72582 Library:NIST05.LIB
SI:83 Formula:C17H32O CAS:55976-05-1 MolWeight:252 RetIndex:1706
CompName:6-Undecen-3-one, 5-butyl-2,2-dimethyl-, (E)- $$ (6E)-5-Butyl-2,2-dimethyl-6-undecen-3-one # $$

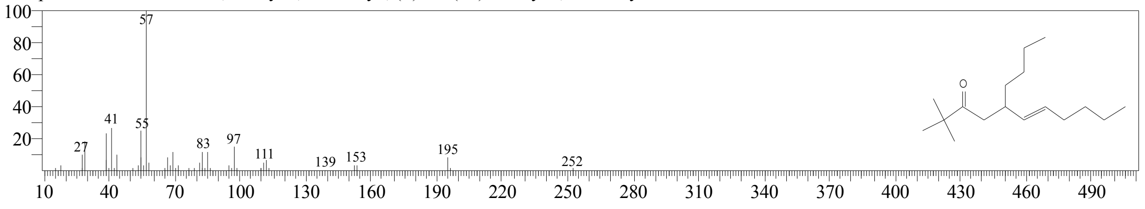

Hit#:2 Entry:23879 Library:NIST05.LIB
SI:83 Formula:C12H24 CAS:123-48-8 MolWeight:168 RetIndex:1030
CompName:3-Heptene, 2,2,4,6,6-pentamethyl- $$ 2,2,4,6,6-Pentamethylheptene-3 $$ 2,2,4,6,6-Pentamethyl-3-heptene $$ 2,2,4,6,6-Pentamethyl-3-heptene,

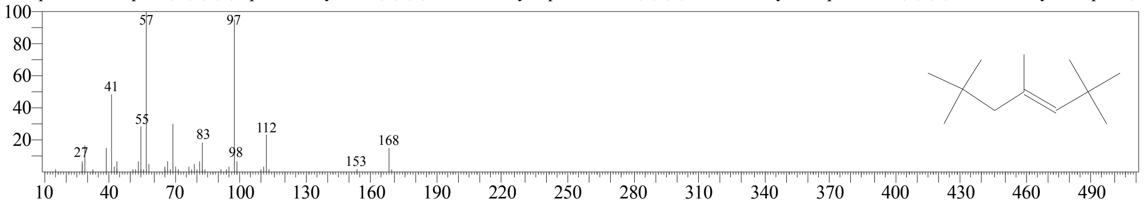

Hit#:3 Entry:65704 Library:NIST05.LIB
SI:82 Formula:C13H23NO3 CAS:0-00-0 MolWeight:241 RetIndex:1743
CompName:4-t-Butyl-2-(1-methyl-2-nitroethyl)cyclohexanone

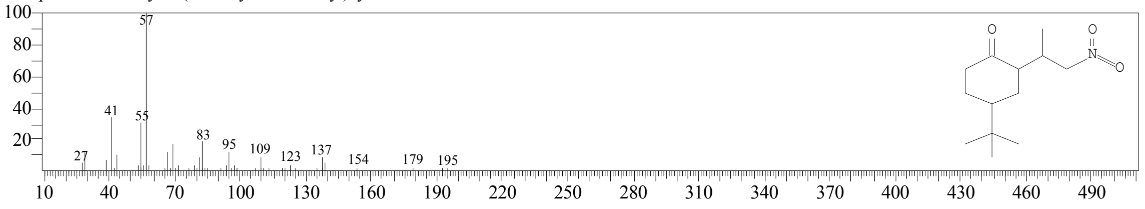

Hit#:4 Entry:142734 Library:NIST05.LIB
SI:82 Formula:C28H56 CAS:55255-73-7 MolWeight:392 RetIndex:2344
CompName:6-Tridecene, 2,2,4,10,12,12-hexamethyl-7-(3,5,5-trimethylhexyl)- $$ 2,2,4,10,12,12-Hexamethyl-7-(3,5,5-trimethylhexyl)-6-tridecene $$

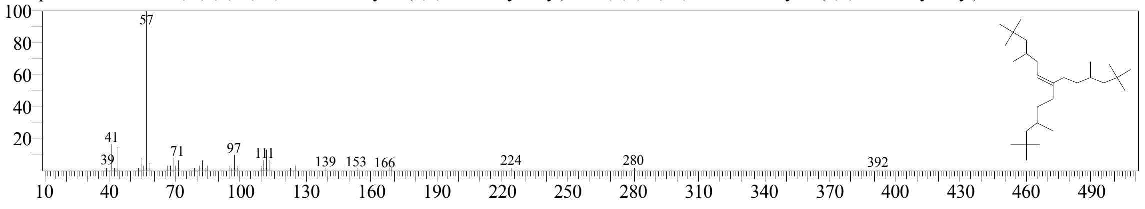

Hit#:5 Entry:23869 Library:NIST05.LIB
SI:82 Formula:C12H24 CAS:74630-52-7 MolWeight:168 RetIndex:1158
CompName:3-Undecene, 6-methyl-, (E)- $$ (3E)-6-Methyl-3-undecene # $$

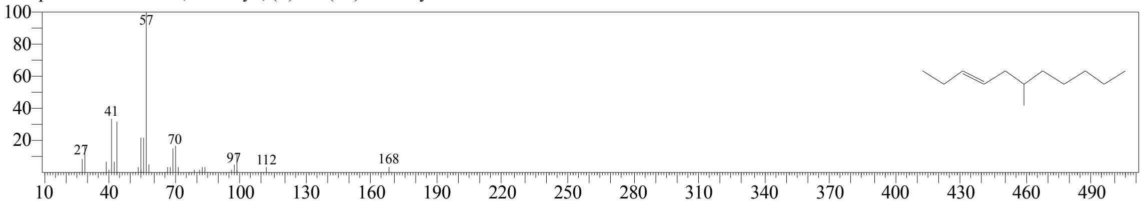

<< Target >>
Line#:13 R.Time:46.095(Scan#:8620) Retention Index:2613 MassPeaks:259
RawMode:Averaged 46.090-46.100(8619-8621) BasePeak:97.10(10795)
BG Mode:Calc. from Peak Group 1 - Event 1 Scan

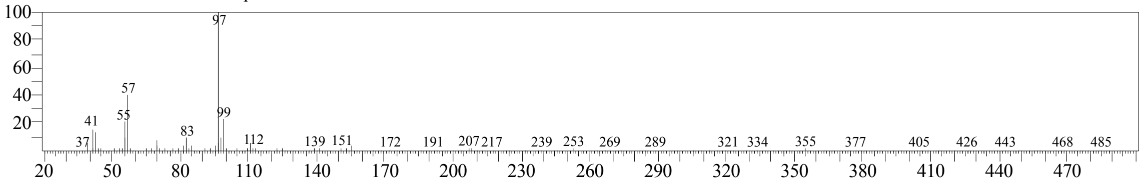

Hit#:1 Entry:99564 Library:NIST11.lib
SI:87 Formula:C14H28O3S CAS:0-00-0 MolWeight:276 RetIndex:2100
CompName:Sulfurous acid, cyclohexylmethyl heptyl ester

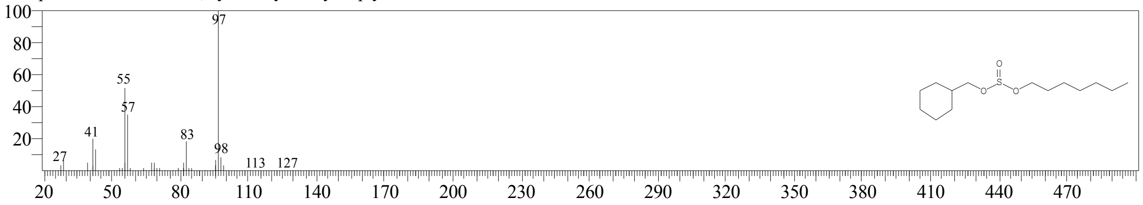

Hit#:2 Entry:88563 Library:NIST11.lib
SI:87 Formula:C13H26O3S CAS:0-00-0 MolWeight:262 RetIndex:2000
CompName:Sulfurous acid, cyclohexylmethyl hexyl ester

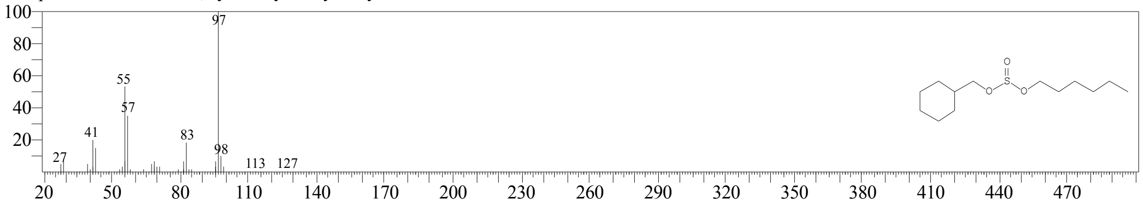

Hit#:3 Entry:86823 Library:NIST05.LIB
SI:87 Formula:C14H28O3S CAS:0-00-0 MolWeight:276 RetIndex:2100
CompName:Sulfurous acid, cyclohexylmethyl heptyl ester

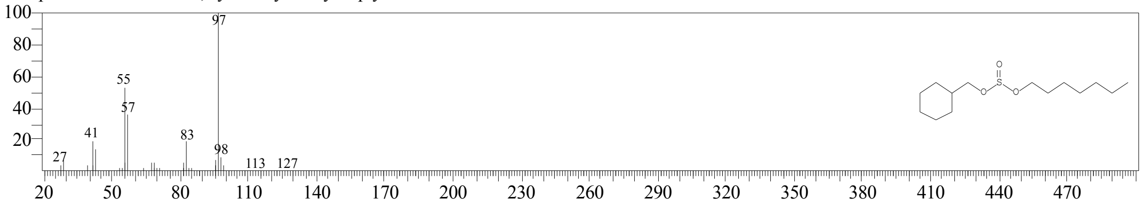

Hit#:4 Entry:78203 Library:NIST05.LIB
SI:87 Formula:C13H26O3S CAS:0-00-0 MolWeight:262 RetIndex:2000
CompName:Sulfurous acid, cyclohexylmethyl hexyl ester

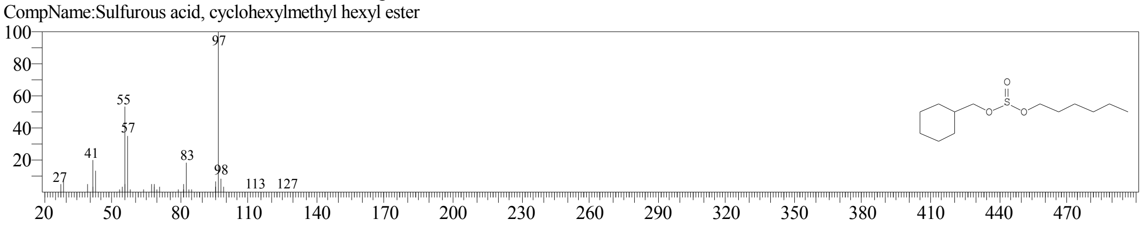

Hit#:5 Entry:150324 Library:NIST05.LIB
SI:86 Formula:C25H50O3S CAS:0-00-0 MolWeight:430 RetIndex:3193
CompName:Sulfurous acid, cyclohexylmethyl octadecyl ester

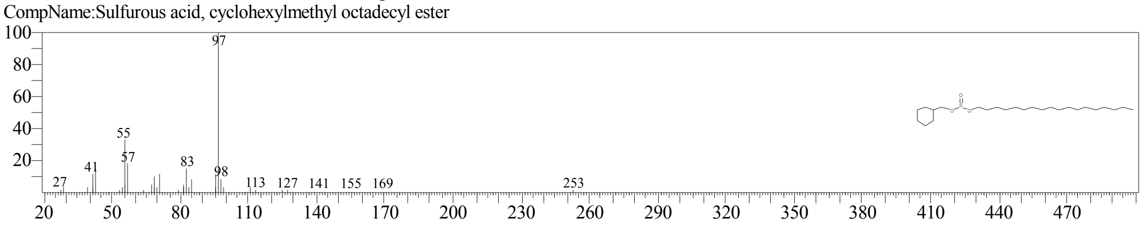

<< Target >>
Line#:14 R.Time:46.705(Scan#:8742) Retention Index:2652 MassPeaks:272
RawMode:Averaged 46.700-46.710(8741-8743) BasePeak:57.10(3928)
BG Mode:Calc. from Peak Group 1 - Event 1 Scan

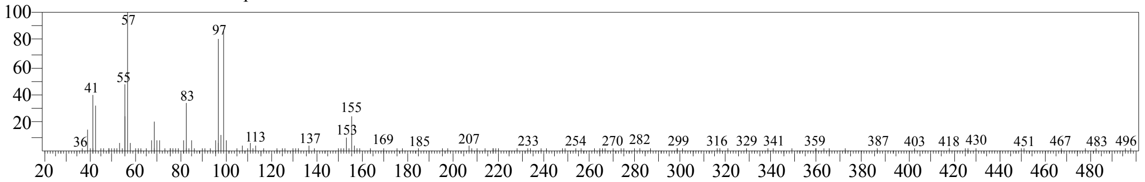

Hit#:1 Entry:61283 Library:NIST05.LIB
SI:81 Formula:C11H23Br CAS:55162-38-4 MolWeight:234 RetIndex:1283
CompName:Nonane, 2-bromo-5-ethyl- $$ 2-Bromo-5-ethylnonane # $$

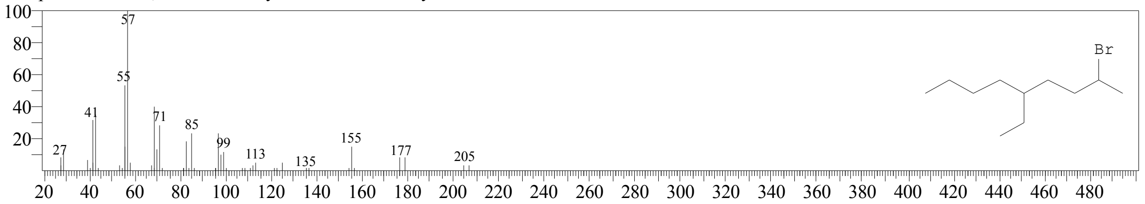

Hit#:2 Entry:18795 Library:NIST11s.lib
SI:80 Formula:C9H20FO2P CAS:333416-06-1 MolWeight:210 RetIndex:0
CompName:2,4,4-Trimethyl-1-pentyl methylphosphonofluoridate $$ Methylphosphonic acid, fluoroanhydride, 2,3,3-trimethylpentyl ester $$ 2,4,4-Trimethy

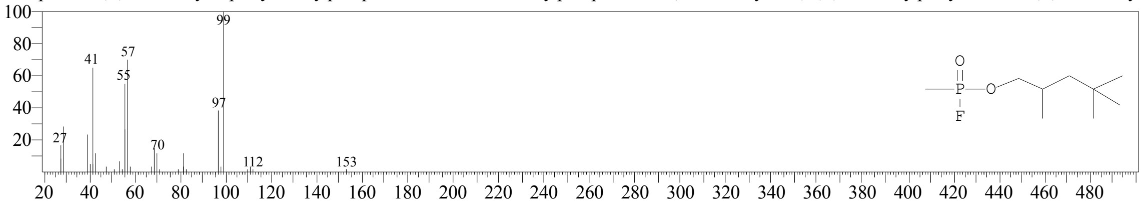

Hit#:3 Entry:23870 Library:NIST05.LIB
SI:80 Formula:C12H24 CAS:0-00-0 MolWeight:168 RetIndex:1097
CompName:1-Hexene, 2,4,4-triethyl-

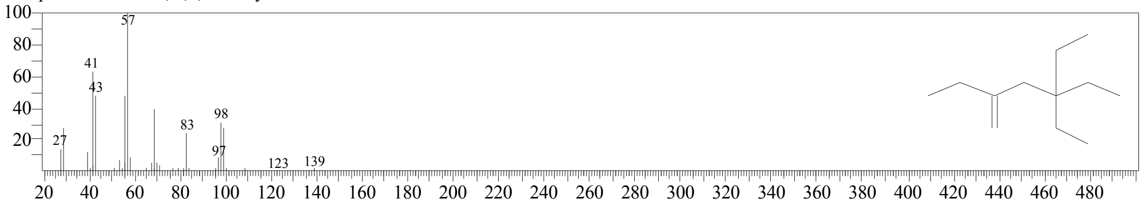

Hit#:4 Entry:137712 Library:NIST05.LIB
SI:79 Formula:C21H45O3P CAS:0-00-0 MolWeight:376 RetIndex:1886
CompName:Phosphite, tris(2,4-dimethylpent-3-yl-

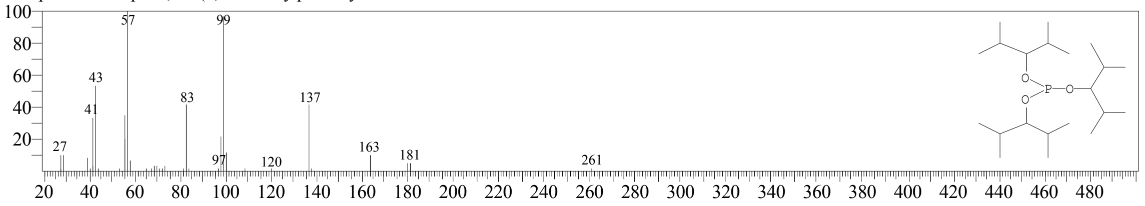

Hit#:5 Entry:171269 Library:NIST11.lib
SI:79 Formula:C21H45O3P CAS:0-00-0 MolWeight:376 RetIndex:1886
CompName:Phosphite, tris(2,4-dimethylpent-3-yl- $$ Tris(1-isopropyl-2-methylpropyl) phosphite # $$

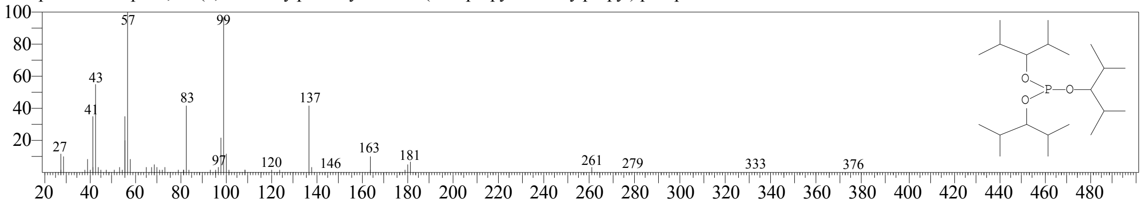

<< Target >>
Line#:15   R.Time:47.535(Scan#:8908) Retention Index:2705 MassPeaks:281
RawMode:Averaged 47.530-47.540(8907-8909) BasePeak:97.10(32062)
BG Mode:Calc. from Peak Group 1 - Event 1 Scan

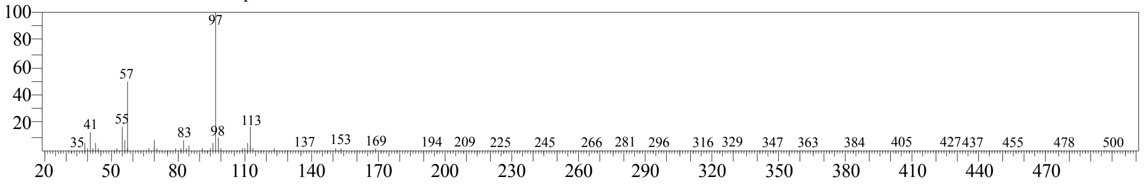

Hit#:1 Entry:78203 Library:NIST05.LIB
SI:86 Formula:C13H26O3S CAS:0-00-0 MolWeight:262 RetIndex:2000
CompName:Sulfurous acid, cyclohexylmethyl hexyl ester

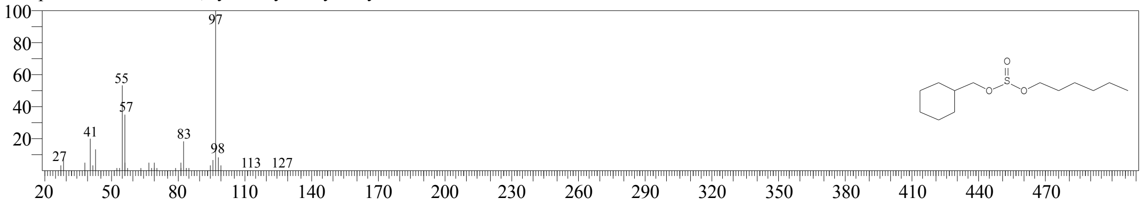

Hit#:2 Entry:88563 Library:NIST11.lib
SI:86 Formula:C13H26O3S CAS:0-00-0 MolWeight:262 RetIndex:2000
CompName:Sulfurous acid, cyclohexylmethyl hexyl ester

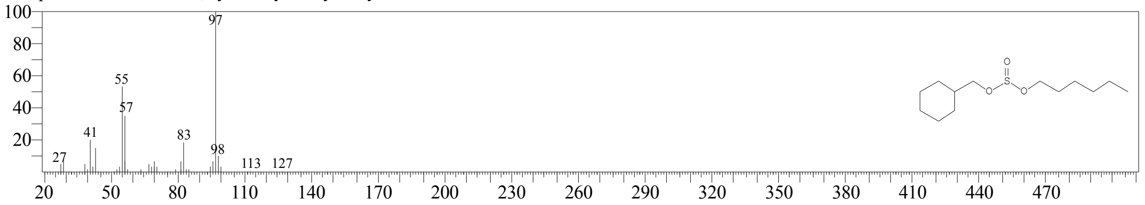

Hit#:3 Entry:99564 Library:NIST11.lib
SI:86 Formula:C14H28O3S CAS:0-00-0 MolWeight:276 RetIndex:2100
CompName:Sulfurous acid, cyclohexylmethyl heptyl ester

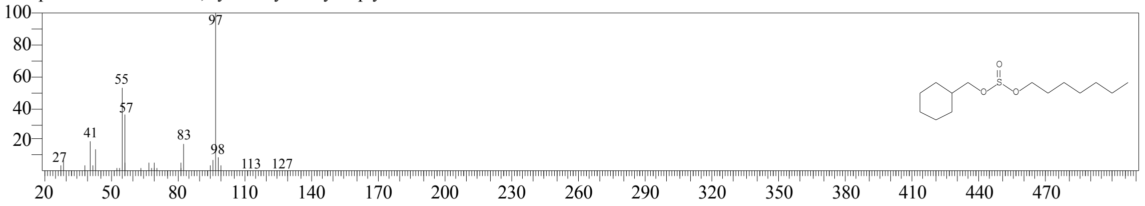

Hit#:4 Entry:86823 Library:NIST05.LIB
SI:86 Formula:C14H28O3S CAS:0-00-0 MolWeight:276 RetIndex:2100
CompName:Sulfurous acid, cyclohexylmethyl heptyl ester

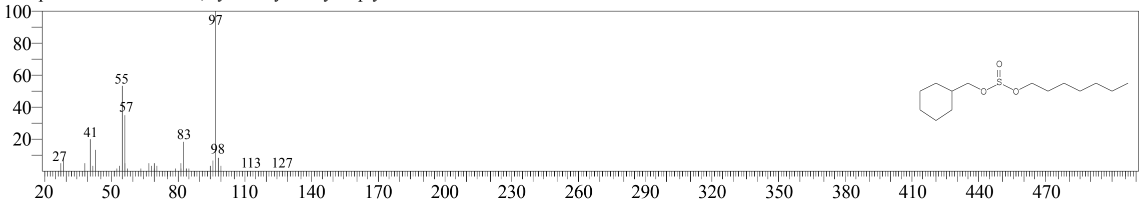

Hit#:5 Entry:125328 Library:NIST05.LIB
SI:86 Formula:C19H38O3S CAS:0-00-0 MolWeight:346 RetIndex:2597
CompName:Sulfurous acid, cyclohexylmethyl dodecyl ester

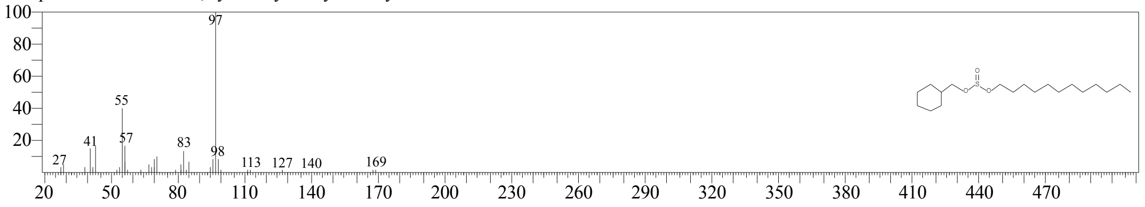

<< Target >>
Line#:16 R.Time:48.135(Scan#:9028) Retention Index:2744 MassPeaks:273
RawMode:Averaged 48.130-48.140(9027-9029) BasePeak:57.10(13926)
BG Mode:Calc. from Peak Group 1 - Event 1 Scan

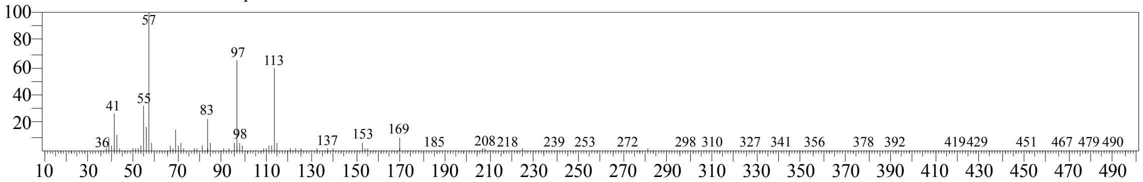

Hit#:1 Entry:23879 Library:NIST05.LIB
SI:81 Formula:C12H24 CAS:123-48-8 MolWeight:168 RetIndex:1030
CompName:3-Heptene, 2,2,4,6,6-pentamethyl- $$ 2,2,4,6,6-Pentamethylheptene-3 $$ 2,2,4,6,6-Pentamethyl-3-heptene $$ 2,2,4,6,6-Pentamethyl-3-heptene,

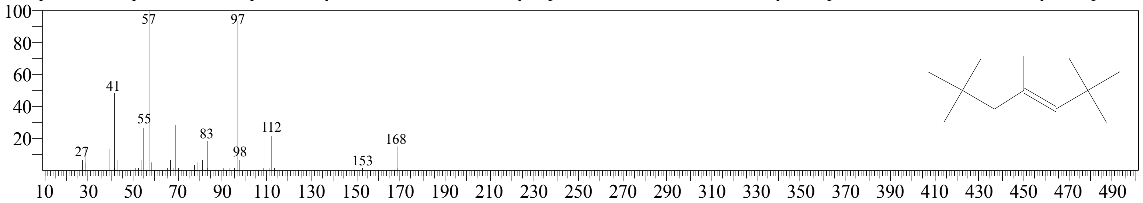

Hit#:2 Entry:142734 Library:NIST05.LIB
SI:80 Formula:C28H56 CAS:55255-73-7 MolWeight:392 RetIndex:2344
CompName:6-Tridecene, 2,2,4,10,12,12-hexamethyl-7-(3,5,5-trimethylhexyl)- $$ 2,2,4,10,12,12-Hexamethyl-7-(3,5,5-trimethylhexyl)-6-tridecene $$

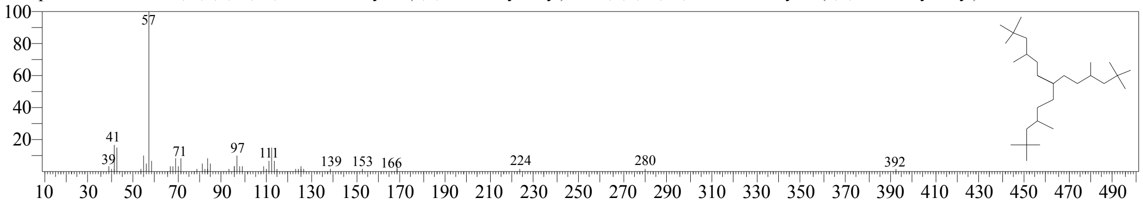

Hit#:3 Entry:72582 Library:NIST05.LIB
SI:79 Formula:C17H32O CAS:55976-05-1 MolWeight:252 RetIndex:1706
CompName:6-Undecen-3-one, 5-butyl-2,2-dimethyl-, (E)- $$ (6E)-5-Butyl-2,2-dimethyl-6-undecen-3-one # $$

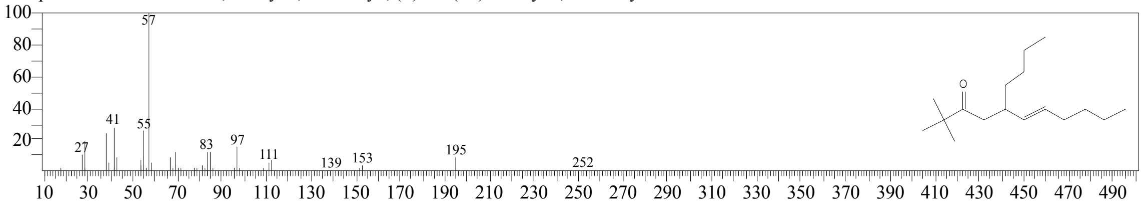

Hit#:4 Entry:23869 Library:NIST05.LIB
SI:79 Formula:C12H24 CAS:74630-52-7 MolWeight:168 RetIndex:1158
CompName:3-Undecene, 6-methyl-, (E)- $$ (3E)-6-Methyl-3-undecene # $$

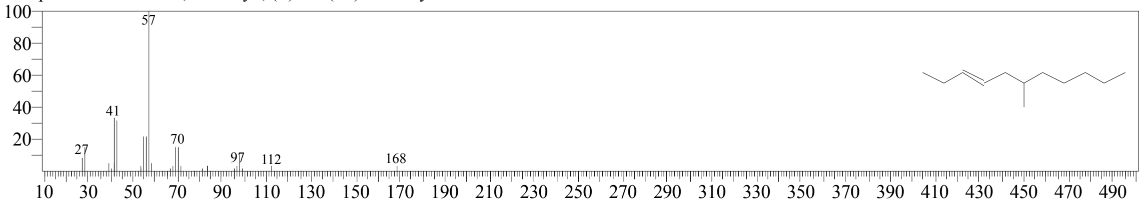

Hit#:5 Entry:135451 Library:NIST05.LIB
SI:79 Formula:C22H42O4 CAS:0-00-0 MolWeight:370 RetIndex:2479
CompName:Oxalic acid, isobutyl hexadecyl ester

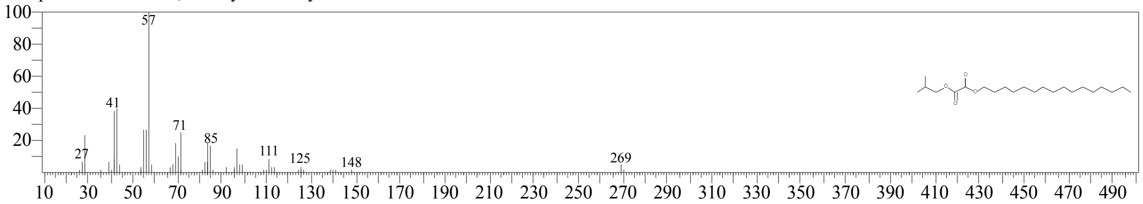

<< Target >>
Line#:17   R.Time:48.550(Scan#:9111) Retention Index:2770 MassPeaks:260
RawMode:Averaged 48.545-48.555(9110-9112) BasePeak:57.10(3918)
BG Mode:Calc. from Peak Group 1 - Event 1 Scan

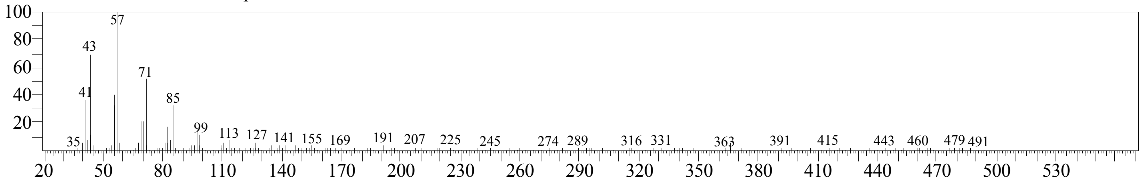

Hit#:1 Entry:27512 Library:NIST05s.LIB
SI:90 Formula:C44H90 CAS:7098-22-8 MolWeight:618 RetIndex:4395
CompName:Tetratetracontane $$ n-Tetratetracontane $$

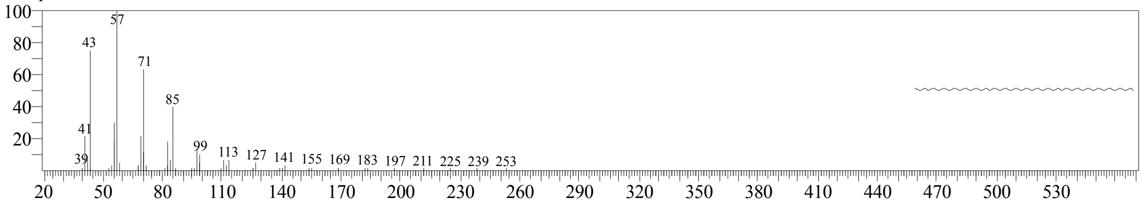

Hit#:2 Entry:27587 Library:NIST05s.LIB
SI:90 Formula:C50H102 CAS:55256-09-2 MolWeight:702 RetIndex:4863
CompName:Triacontane, 11,20-didecyl- $$ 11,20-Di-n-decyltriacontane $$ 11,20-Didecyltriacontane # $$

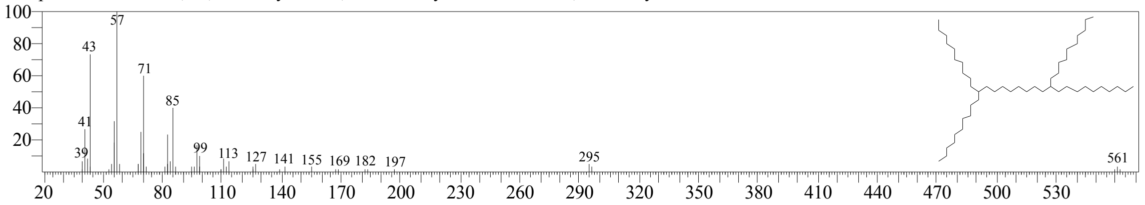

Hit#:3 Entry:207485 Library:NIST11.lib
SI:90 Formula:C38H78 CAS:55517-73-2 MolWeight:534 RetIndex:3734
CompName:Hexacosane, 13-dodecyl- $$ 13-n-Dodecylhexacosane $$ 13-Dodecylhexacosane # $$

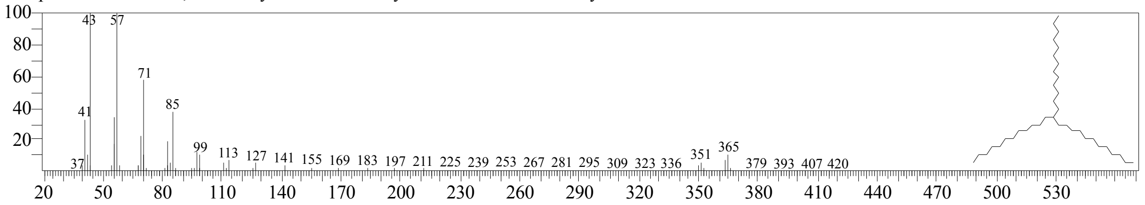

Hit#:4 Entry:161061 Library:NIST05.LIB
SI:89 Formula:C43H88 CAS:55162-61-3 MolWeight:604 RetIndex:4103
CompName:Tetracontane, 3,5,24-trimethyl- $$ 3,5,24-Trimethyltetracontane # $$

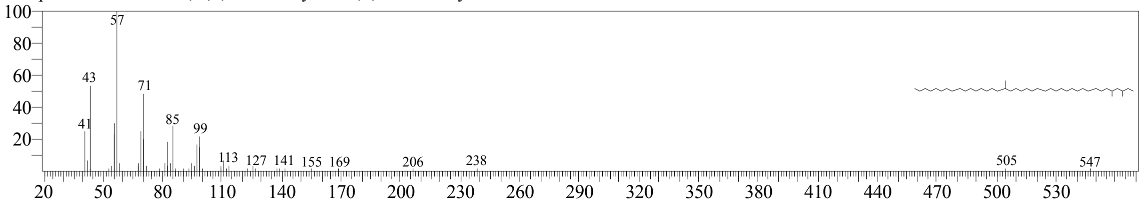

Hit#:5 Entry:22092 Library:NIST05s.LIB
SI:89 Formula:C19H40 CAS:1560-88-9 MolWeight:268 RetIndex:1846
CompName:Octadecane, 2-methyl- $$ 2-Methyloctadecane $$

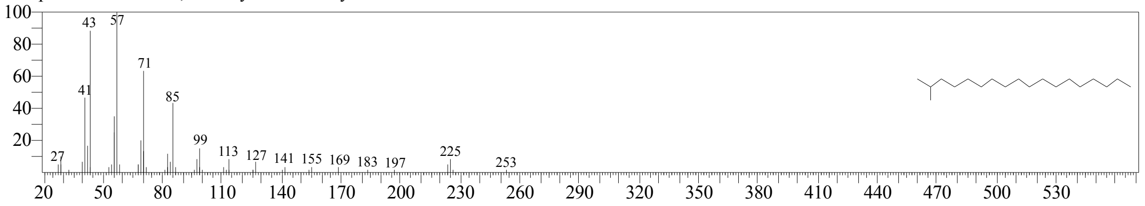

<< Target >>
Line#:18   R.Time:49.470(Scan#:9295) Retention Index:2829! MassPeaks:270
RawMode:Averaged 49.465-49.475(9294-9296) BasePeak:69.05(4083)
BG Mode:Calc. from Peak Group 1 - Event 1 Scan

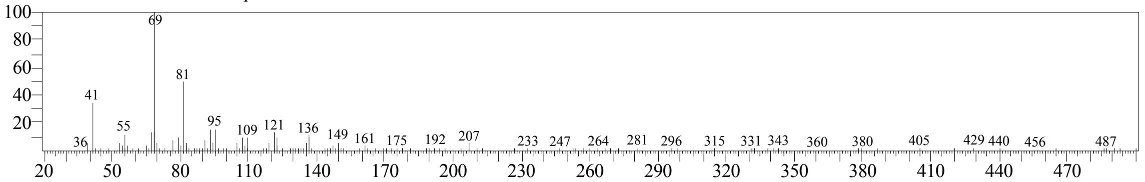

Hit#:1 Entry:146890 Library:NIST05.LIB
SI:90 Formula:C30H50 CAS:111-02-4 MolWeight:410 RetIndex:2914
CompName:2,6,10,14,18,22-Tetracosahexaene, 2,6,10,15,19,23-hexamethyl-, (all-E)- $$ All-trans-Squalene $$ trans-Squalene $$ Spinacen $$ Spinacene $$

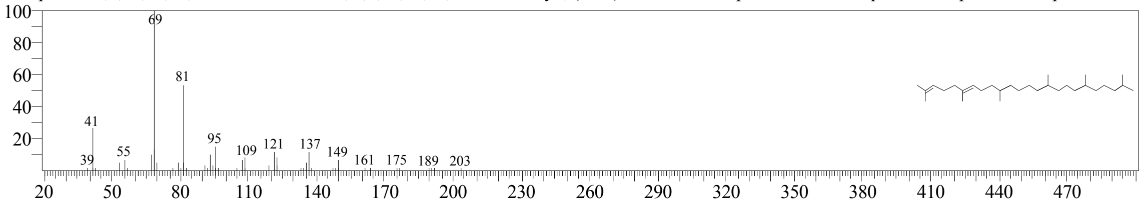

Hit#:2 Entry:26670 Library:NIST05s.LIB
SI:90 Formula:C30H50 CAS:7683-64-9 MolWeight:410 RetIndex:2914
CompName:Squalene $$ 2,6,10,14,18,22-Tetracosahexaene, 2,6,10,15,19,23-hexamethyl- $$ Skvalen $$ Spinacene $$ Supraene $$ (6E,10E,14E,18E)-2,6,

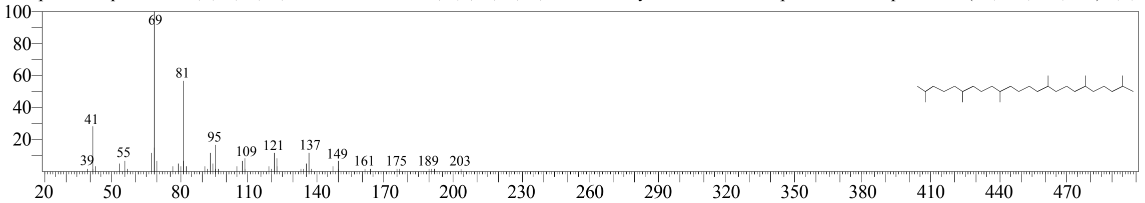

Hit#:3 Entry:23344 Library:NIST05s.LIB
SI:89 Formula:C20H34O CAS:7614-21-3 MolWeight:290 RetIndex:2192
CompName:Hexadeca-2,6,10,14-tetraen-1-ol, 3,7,11,16-tetramethyl-, (E,E,E)- $$ (2Z,6E,10E)-3,7,11,15-Tetramethyl-2,6,10,14-hexadecatetraen-1-ol # $$

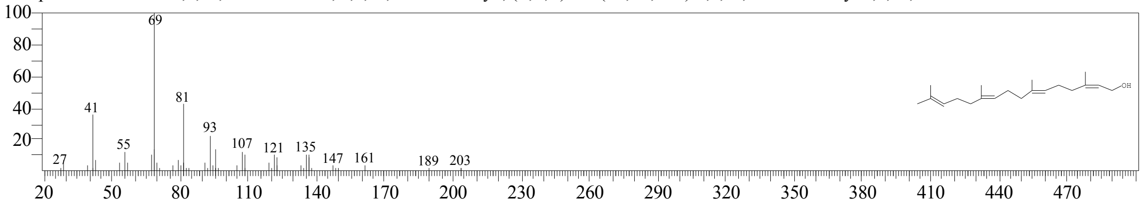

Hit#:4 Entry:123688 Library:NIST05.LIB
SI:88 Formula:C25H42 CAS:75581-03-2 MolWeight:342 RetIndex:2432
CompName:2,6,10,14,18-Pentamethyl-2,6,10,14,18-eicosapentaene $$ (6E,10E,14E,18E)-2,6,10,14,18-Pentamethyl-2,6,10,14,18-icosapentaene # $$

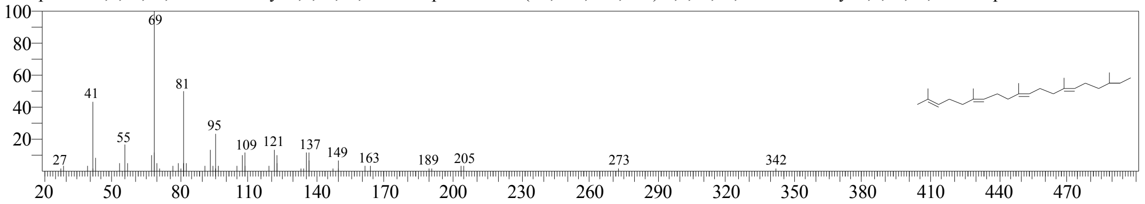

Hit#:5 Entry:140338 Library:NIST05.LIB
SI:86 Formula:C27H44O CAS:0-00-0 MolWeight:384 RetIndex:2819
CompName:Docosa-2,6,10,14,18-pentaen-22-al, 2,6,10,15,18-pentamethyl-, all-trans

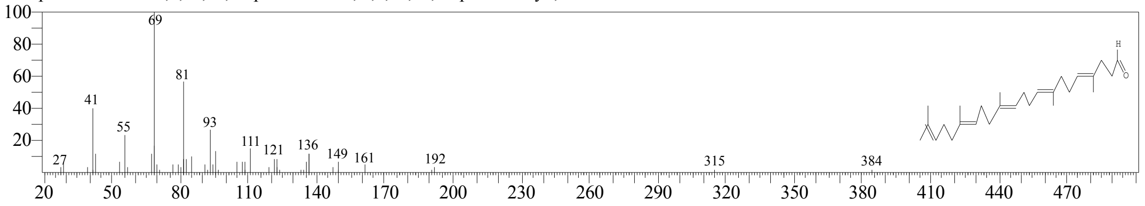

<< Target >>
Line#:19 R.Time:50.350(Scan#:9471) Retention Index:2886! MassPeaks:300
RawMode:Averaged 50.345-50.355(9470-9472) BasePeak:57.10(18869)
BG Mode:Calc. from Peak Group 1 - Event 1 Scan

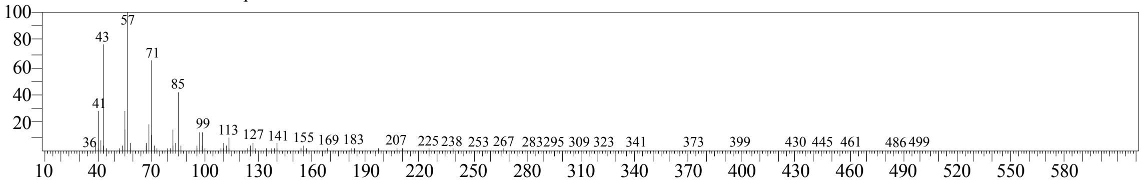

Hit#:1 Entry:30812 Library:NIST11s.lib
SI:97 Formula:C44H90 CAS:7098-22-8 MolWeight:618 RetIndex:4395
CompName:Tetratetracontane $$ n-Tetratetracontane $$

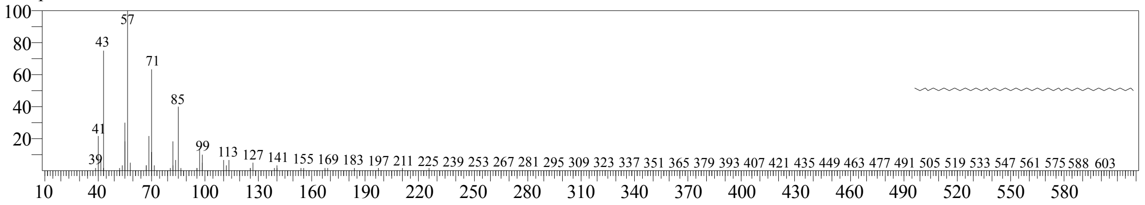

Hit#:2 Entry:26171 Library:NIST05s.LIB
SI:97 Formula:C27H56 CAS:593-49-7 MolWeight:380 RetIndex:2705
CompName:Heptacosane $$ n-Heptacosane $$

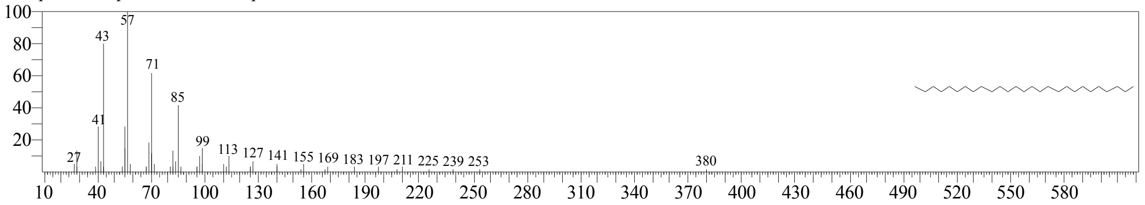

Hit#:3 Entry:201892 Library:NIST11.lib
SI:96 Formula:C34H70 CAS:55429-84-0 MolWeight:478 RetIndex:3337
CompName:Tetracosane, 11-decyl- $$ 11-n-Decyltetracosane $$ 11-Decyltetracosane # $$

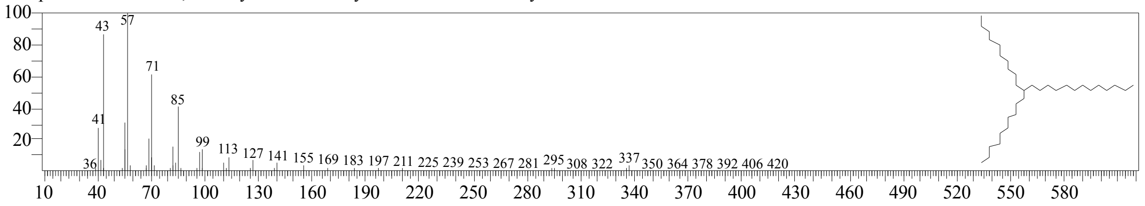

Hit#:4 Entry:27217 Library:NIST05s.LIB
SI:96 Formula:C34H70 CAS:14167-59-0 MolWeight:478 RetIndex:3401
CompName:Tetratriacontane $$ n-Tetratriacontane $$

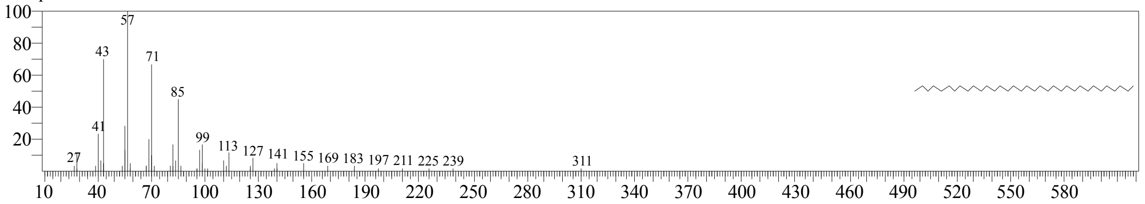

Hit#:5 Entry:30613 Library:NIST11s.lib
SI:96 Formula:C36H74 CAS:630-06-8 MolWeight:506 RetIndex:3600
CompName:Hexatriacontane $$ n-Hexatriacontane $$

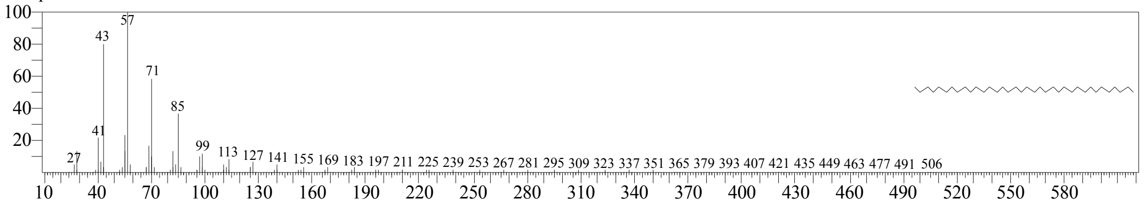

<< Target >>
Line#:20 R.Time:51.130(Scan#:9627) Retention Index:2936! MassPeaks:302
RawMode:Averaged 51.125-51.135(9626-9628) BasePeak:97.10(18559)
BG Mode:Calc. from Peak Group 1 - Event 1 Scan

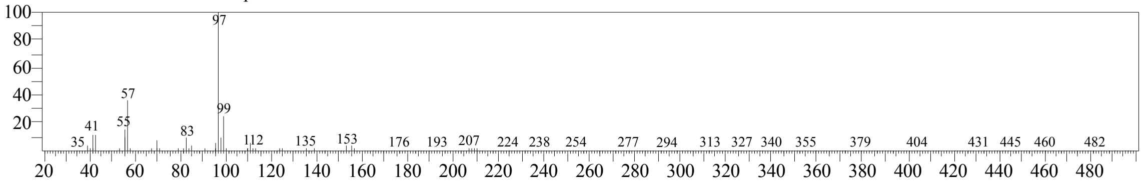

Hit#:1 Entry:177164 Library:NIST11.lib
SI:85 Formula:C22H44O3S CAS:0-00-0 MolWeight:388 RetIndex:2895
CompName:Sulfurous acid, cyclohexylmethyl pentadecyl ester

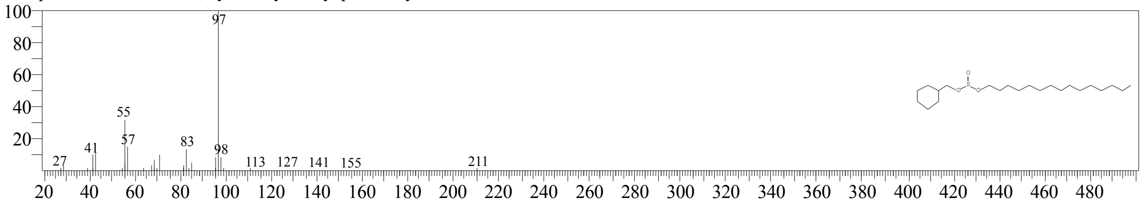

Hit#:2 Entry:141493 Library:NIST05.LIB
SI:85 Formula:C22H44O3S CAS:0-00-0 MolWeight:388 RetIndex:2895
CompName:Sulfurous acid, cyclohexylmethyl pentadecyl ester

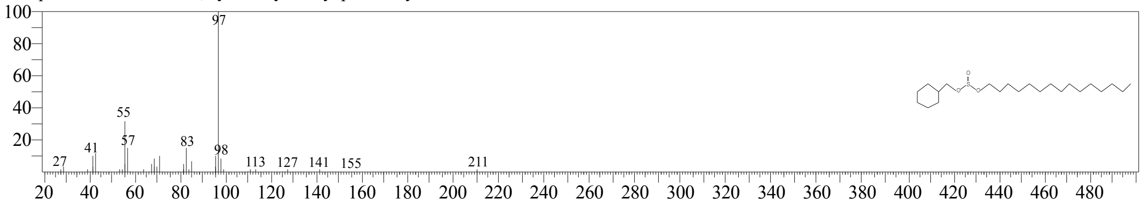

Hit#:3 Entry:150324 Library:NIST05.LIB
SI:85 Formula:C25H50O3S CAS:0-00-0 MolWeight:430 RetIndex:3193
CompName:Sulfurous acid, cyclohexylmethyl octadecyl ester

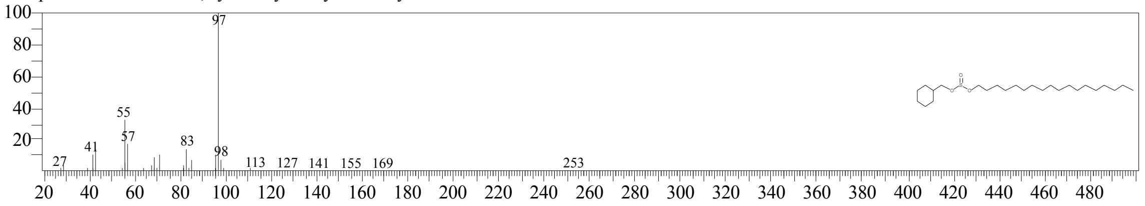

Hit#:4 Entry:192100 Library:NIST11.lib
SI:85 Formula:C25H50O3S CAS:0-00-0 MolWeight:430 RetIndex:3193
CompName:Sulfurous acid, cyclohexylmethyl octadecyl ester

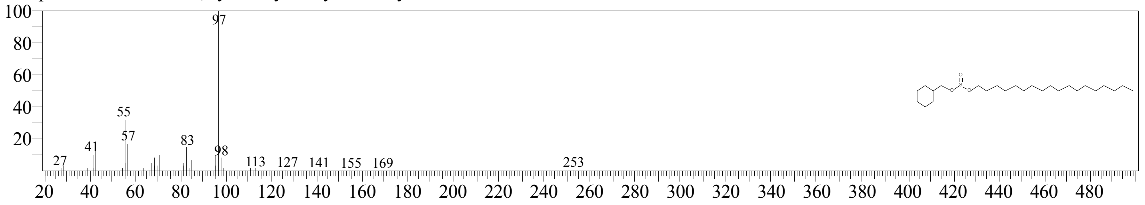

Hit#:5 Entry:88563 Library:NIST11.lib
SI:85 Formula:C13H26O3S CAS:0-00-0 MolWeight:262 RetIndex:2000
CompName:Sulfurous acid, cyclohexylmethyl hexyl ester

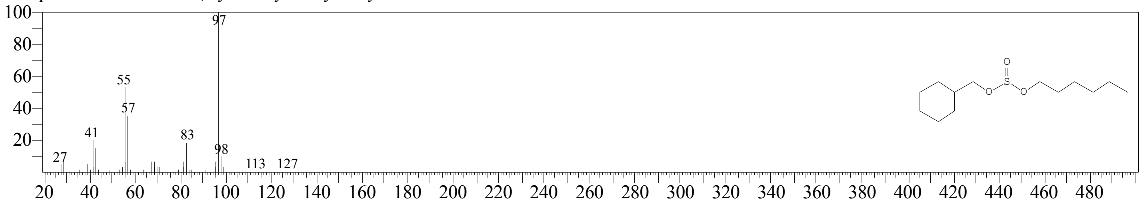

<< Target >>
Line#:21 R.Time:51.375(Scan#:9676) Retention Index:2952! MassPeaks:290
RawMode:Averaged 51.370-51.380(9675-9677) BasePeak:57.10(10245)
BG Mode:Calc. from Peak Group 1 - Event 1 Scan

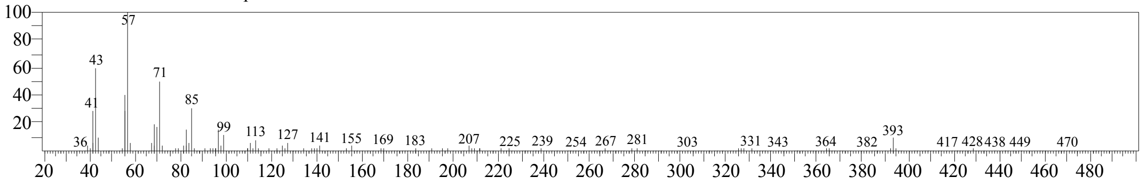

Hit#:1 Entry:26171 Library:NIST05s.LIB
SI:91 Formula:C27H56 CAS:593-49-7 MolWeight:380 RetIndex:2705
CompName:Heptacosane $$ n-Heptacosane $$

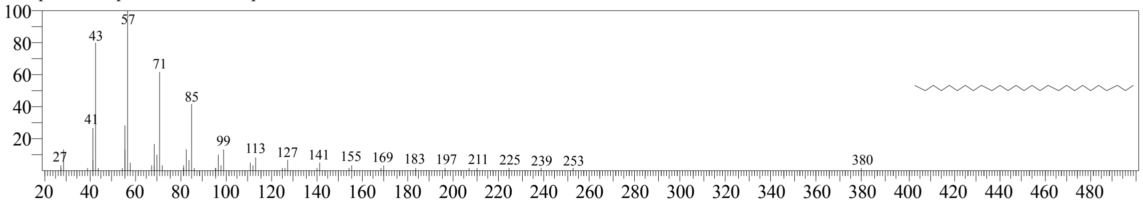

Hit#:2 Entry:161325 Library:NIST05.LIB
SI:91 Formula:C44H90 CAS:7098-22-8 MolWeight:618 RetIndex:4395
CompName:Tetratetracontane $$ n-Tetratetracontane $$

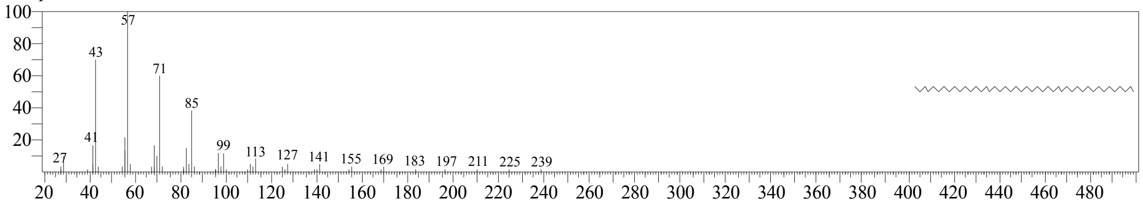

Hit#:3 Entry:27321 Library:NIST05s.LIB
SI:91 Formula:C36H74 CAS:630-06-8 MolWeight:506 RetIndex:3600
CompName:Hexatriacontane $$ n-Hexatriacontane $$

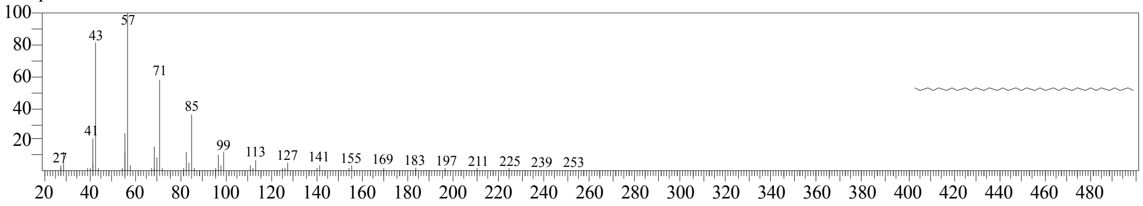

Hit#:4 Entry:27217 Library:NIST05s.LIB
SI:91 Formula:C34H70 CAS:14167-59-0 MolWeight:478 RetIndex:3401
CompName:Tetratriacontane $$ n-Tetratriacontane $$

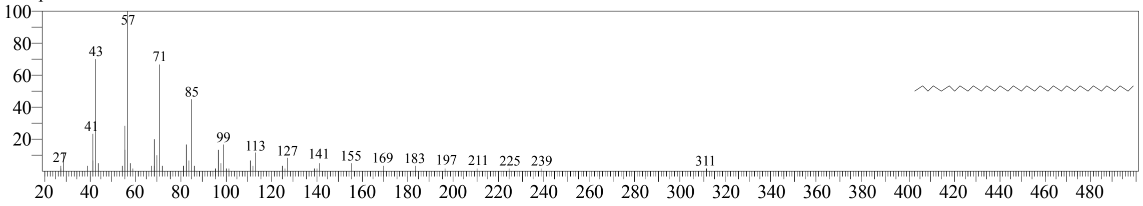

Hit#:5 Entry:27047 Library:NIST05s.LIB
SI:91 Formula:C32H66 CAS:544-85-4 MolWeight:450 RetIndex:3202
CompName:Dotriacontane $$ n-Dotriacontane $$ Bicetyl $$

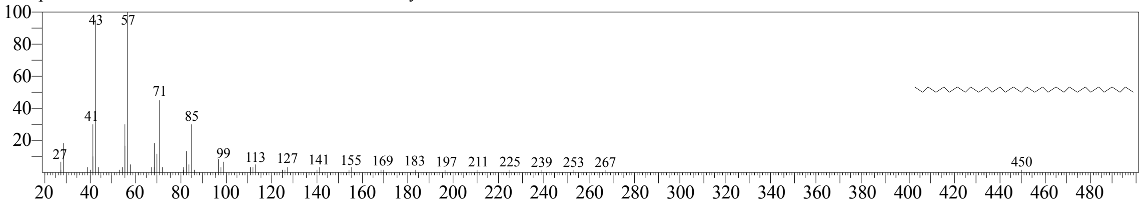

<< Target >>
Line#:22 R.Time:51.700(Scan#:9741) Retention Index:2973! MassPeaks:233
RawMode:Averaged 51.695-51.705(9740-9742) BasePeak:57.10(7074)
BG Mode:Calc. from Peak Group 1 - Event 1 Scan

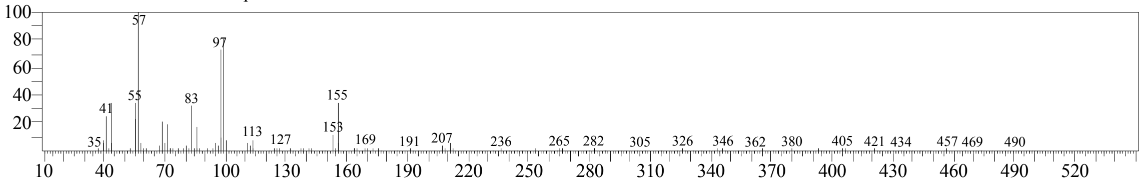

Hit#:1 Entry:61283 Library:NIST05.LIB
SI:82 Formula:C11H23Br CAS:55162-38-4 MolWeight:234 RetIndex:1283
CompName:Nonane, 2-bromo-5-ethyl- $$ 2-Bromo-5-ethylnonane # $$

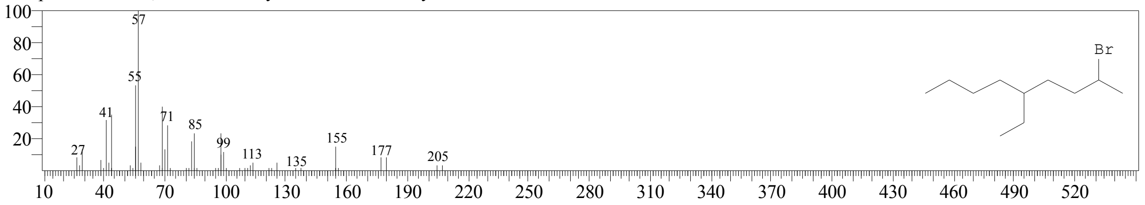

Hit#:2 Entry:161061 Library:NIST05.LIB
SI:81 Formula:C43H88 CAS:55162-61-3 MolWeight:604 RetIndex:4103
CompName:Tetracontane, 3,5,24-trimethyl- $$ 3,5,24-Trimethyltetracontane # $$

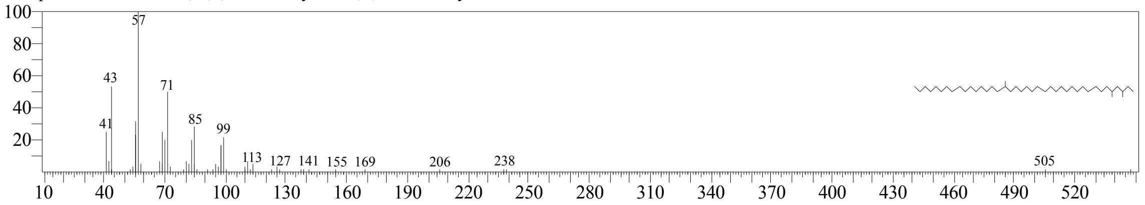

Hit#:3 Entry:135513 Library:NIST05.LIB
SI:80 Formula:C23H46O3 CAS:0-00-0 MolWeight:370 RetIndex:2487
CompName:Carbonic acid, isobutyl octadecyl ester

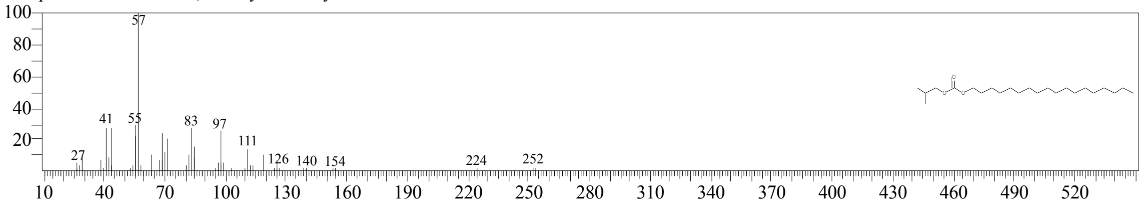

Hit#:4 Entry:142072 Library:NIST05.LIB
SI:80 Formula:C22H46O3S CAS:0-00-0 MolWeight:390 RetIndex:2831
CompName:Sulfurous acid, butyl octadecyl ester

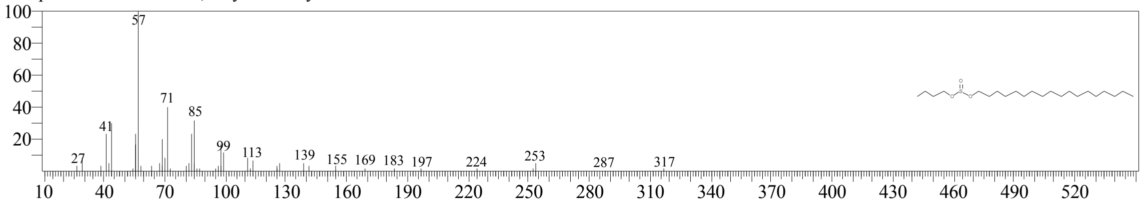

Hit#:5 Entry:33460 Library:NIST05.LIB
SI:80 Formula:C12H26O CAS:2370-15-2 MolWeight:186 RetIndex:1372
CompName:1-Decanol, 2,2-dimethyl- $$ 1-Hydroxy-2,2-dimethyldecane $$ 2,2-Dimethyl-1-decanol # $$

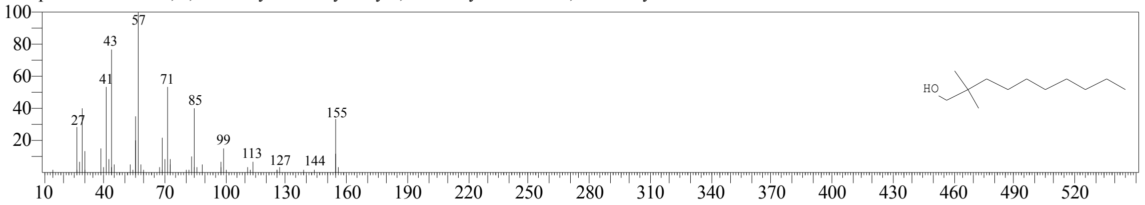

<< Target >>
Line#:23 R.Time:52.345(Scan#:9870) Retention Index:3014! MassPeaks:245
RawMode:Averaged 52.340-52.350(9869-9871) BasePeak:57.10(3189)
BG Mode:Calc. from Peak Group 1 - Event 1 Scan

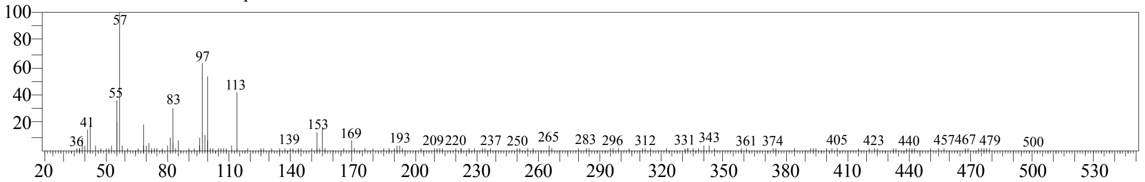

Hit#:1 Entry:135513 Library:NIST05.LIB
SI:75 Formula:C23H46O3 CAS:0-00-0 MolWeight:370 RetIndex:2487
CompName:Carbonic acid, isobutyl octadecyl ester

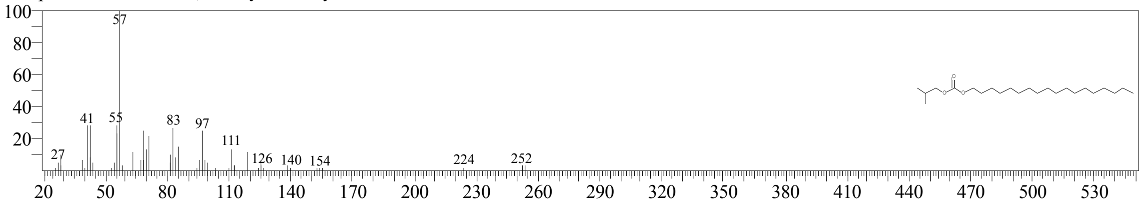

Hit#:2 Entry:61283 Library:NIST05.LIB
SI:75 Formula:C11H23Br CAS:55162-38-4 MolWeight:234 RetIndex:1283
CompName:Nonane, 2-bromo-5-ethyl- $$ 2-Bromo-5-ethylnonane # $$

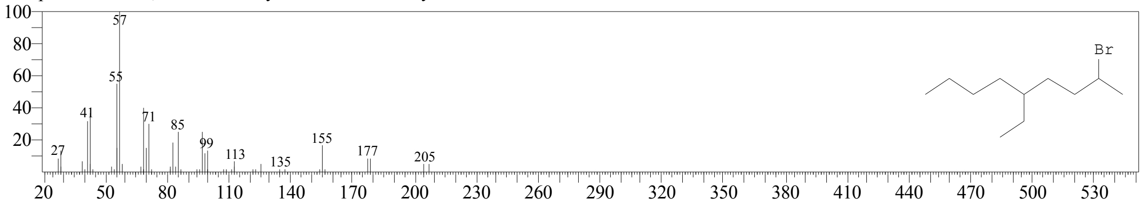

Hit#:3 Entry:129852 Library:NIST05.LIB
SI:75 Formula:C22H44O3 CAS:0-00-0 MolWeight:356 RetIndex:2387
CompName:Carbonic acid, heptadecyl isobutyl ester

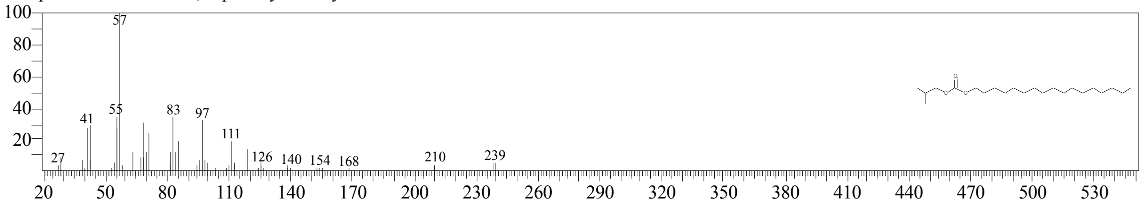

Hit#:4 Entry:161061 Library:NIST05.LIB
SI:75 Formula:C43H88 CAS:55162-61-3 MolWeight:604 RetIndex:4103
CompName:Tetracontane, 3,5,24-trimethyl- $$ 3,5,24-Trimethyltetracontane # $$

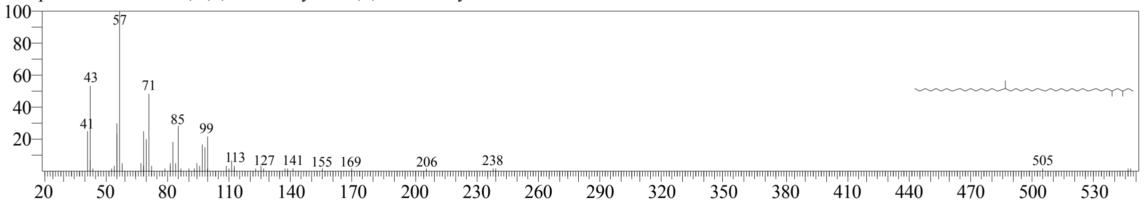

Hit#:5 Entry:116536 Library:NIST05.LIB
SI:75 Formula:C20H40O3 CAS:0-00-0 MolWeight:328 RetIndex:2188
CompName:Carbonic acid, isobutyl pentadecyl ester

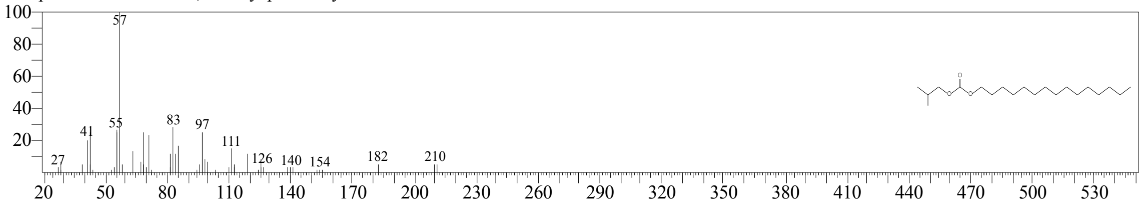

<< Target >>
Line#:24 R.Time:52.455(Scan#:9892) Retention Index:3021! MassPeaks:286
RawMode:Averaged 52.450-52.460(9891-9893) BasePeak:97.10(52510)
BG Mode:Calc. from Peak Group 1 - Event 1 Scan

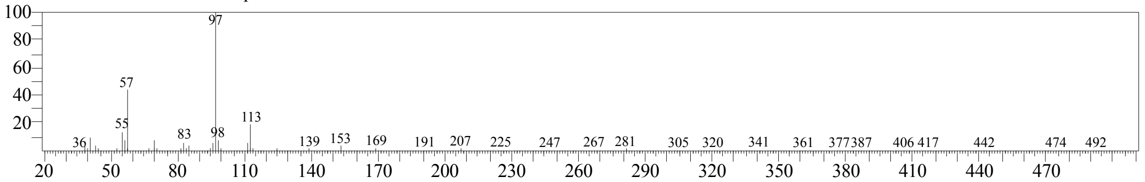

Hit#:1 Entry:150324 Library:NIST05.LIB
SI:85 Formula:C25H50O3S CAS:0-00-0 MolWeight:430 RetIndex:3193
CompName:Sulfurous acid, cyclohexylmethyl octadecyl ester

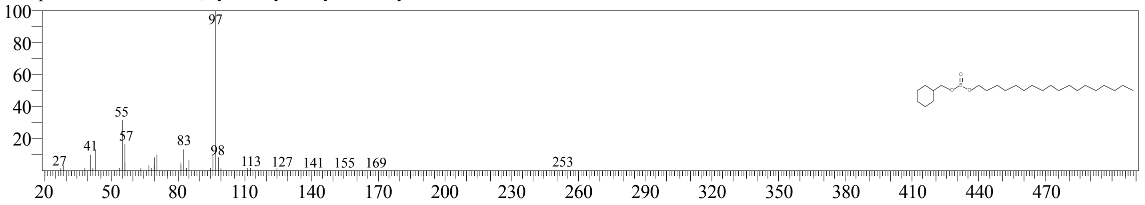

Hit#:2 Entry:192100 Library:NIST11.lib
SI:85 Formula:C25H50O3S CAS:0-00-0 MolWeight:430 RetIndex:3193
CompName:Sulfurous acid, cyclohexylmethyl octadecyl ester

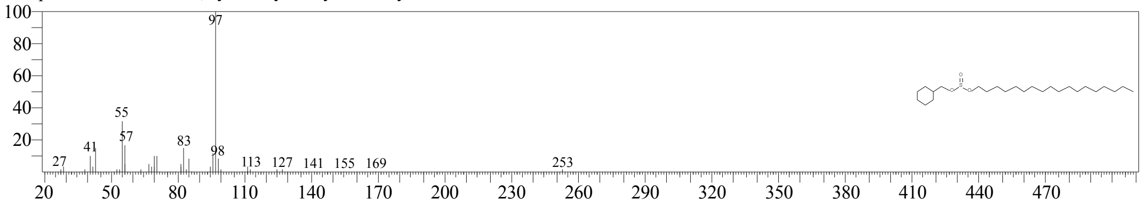

Hit#:3 Entry:141493 Library:NIST05.LIB
SI:84 Formula:C22H44O3S CAS:0-00-0 MolWeight:388 RetIndex:2895
CompName:Sulfurous acid, cyclohexylmethyl pentadecyl ester

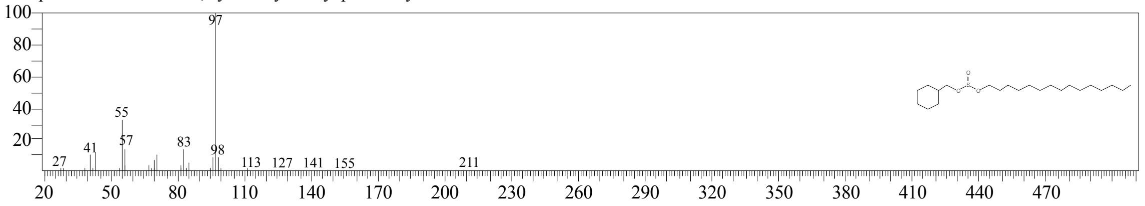

Hit#:4 Entry:177164 Library:NIST11.lib
SI:84 Formula:C22H44O3S CAS:0-00-0 MolWeight:388 RetIndex:2895
CompName:Sulfurous acid, cyclohexylmethyl pentadecyl ester

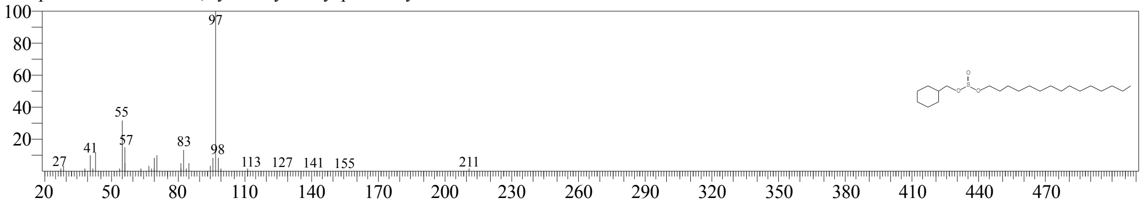

Hit#:5 Entry:125328 Library:NIST05.LIB
SI:84 Formula:C19H38O3S CAS:0-00-0 MolWeight:346 RetIndex:2597
CompName:Sulfurous acid, cyclohexylmethyl dodecyl ester

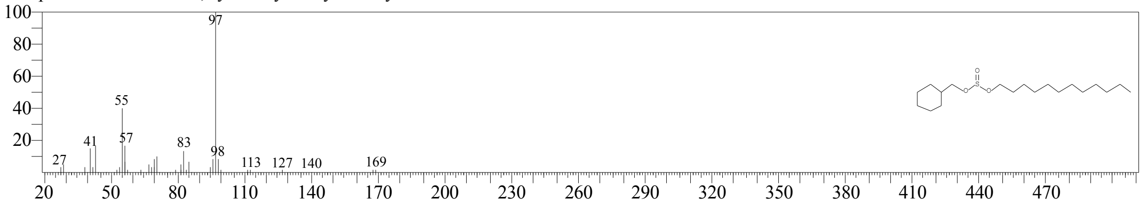

<< Target >>
Line#:25   R.Time:52.560(Scan#:9913) Retention Index:3028! MassPeaks:256
RawMode:Averaged 52.555-52.565(9912-9914) BasePeak:57.10(4745)
BG Mode:Calc. from Peak Group 1 - Event 1 Scan

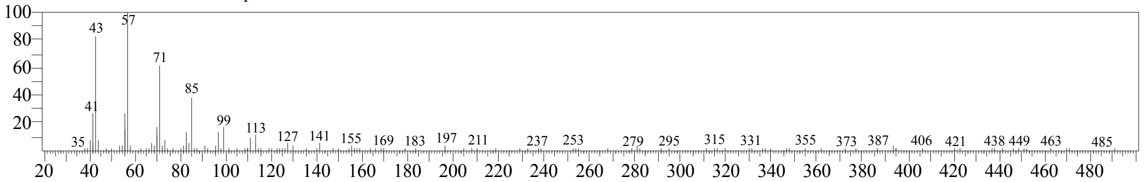

Hit#:1 Entry:27321 Library:NIST05s.LIB
SI:92 Formula:C36H74 CAS:630-06-8 MolWeight:506 RetIndex:3600
CompName:Hexatriacontane $$ n-Hexatriacontane $$

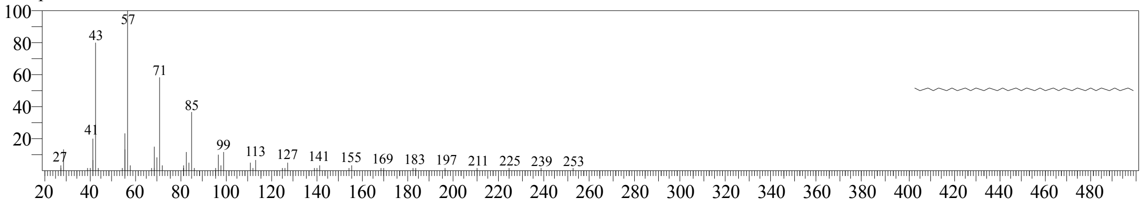

Hit#:2 Entry:26171 Library:NIST05s.LIB
SI:91 Formula:C27H56 CAS:593-49-7 MolWeight:380 RetIndex:2705
CompName:Heptacosane $$ n-Heptacosane $$

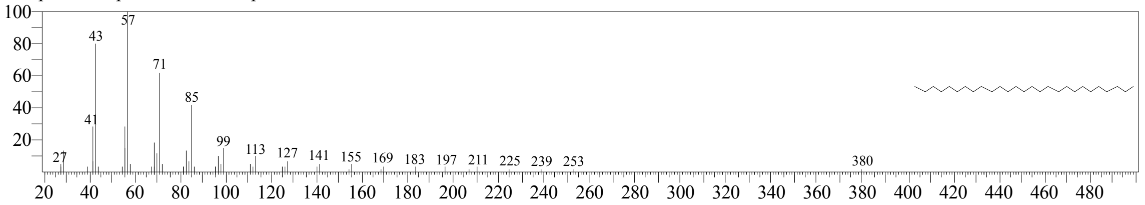

Hit#:3 Entry:98898 Library:NIST05.LIB
SI:91 Formula:C21H44 CAS:1560-84-5 MolWeight:296 RetIndex:2045
CompName:Eicosane, 2-methyl- $$ 2-Methyleicosane $$ 2-Methylicosane # $$

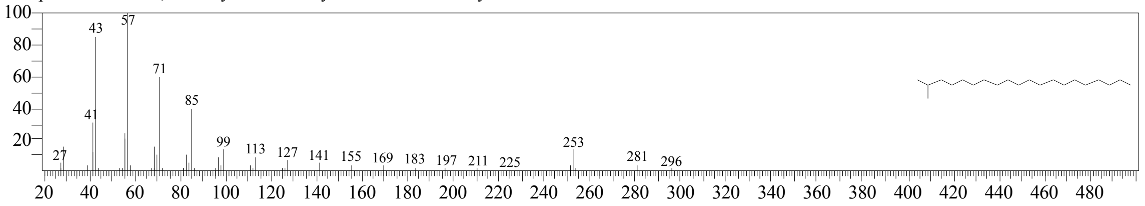

Hit#:4 Entry:201892 Library:NIST11.lib
SI:91 Formula:C34H70 CAS:55429-84-0 MolWeight:478 RetIndex:3337
CompName:Tetracosane, 11-decyl- $$ 11-n-Decyltetracosane $$ 11-Decyltetracosane # $$

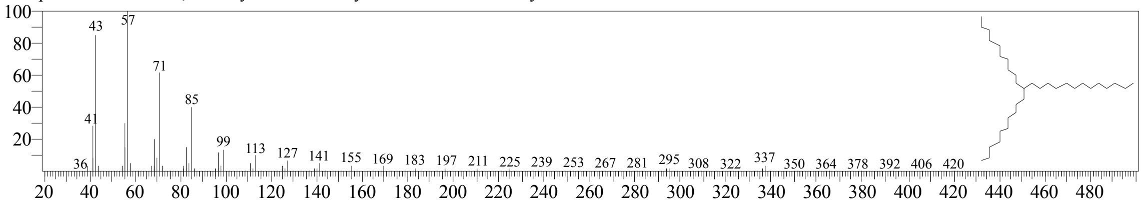

Hit#:5 Entry:165575 Library:NIST11.lib
SI:91 Formula:C26H54 CAS:55333-99-8 MolWeight:366 RetIndex:2542
CompName:Eicosane, 7-hexyl- $$ 7-n-Hexyleicosane $$ 7-Hexylicosane # $$

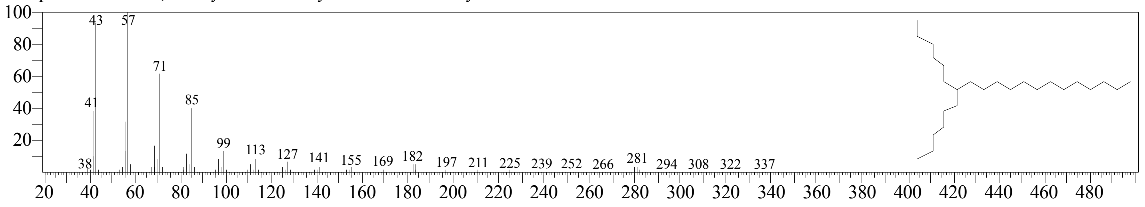

<< Target >>
Line#:26   R.Time:53.050(Scan#:10011) Retention Index:3059! MassPeaks:332
RawMode:Averaged 53.045-53.055(10010-10012) BasePeak:57.10(67109)
BG Mode:Calc. from Peak Group 1 - Event 1 Scan

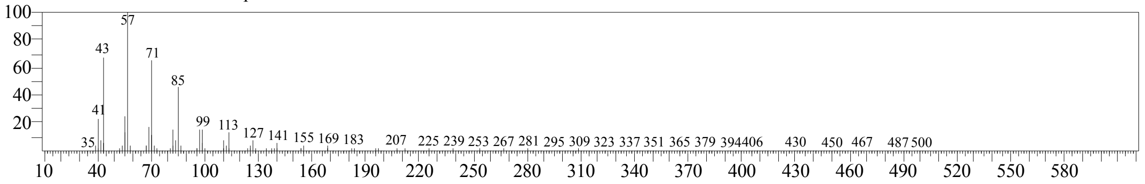

Hit#:1 Entry:27217 Library:NIST05s.LIB
SI:97 Formula:C34H70 CAS:14167-59-0 MolWeight:478 RetIndex:3401
CompName:Tetratriacontane $$ n-Tetratriacontane $$

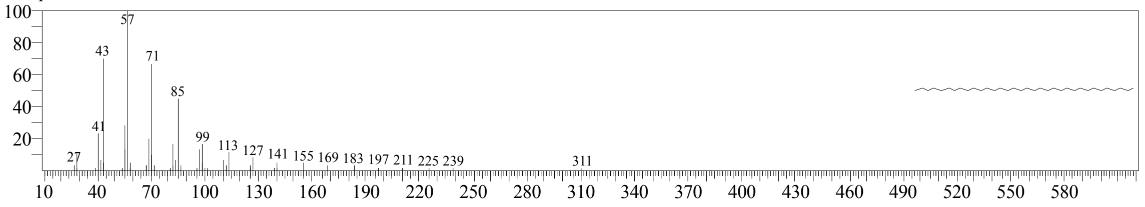

Hit#:2 Entry:185354 Library:NIST11.lib
SI:96 Formula:C29H60 CAS:0-00-0 MolWeight:408 RetIndex:2840
CompName:2-methyloctacosane

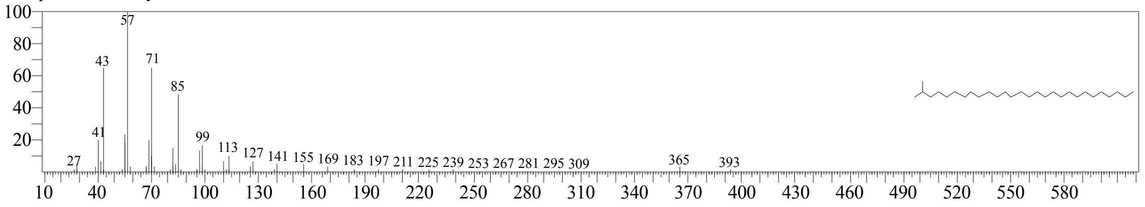

Hit#:3 Entry:30813 Library:NIST11s.lib
SI:96 Formula:C44H90 CAS:7098-22-8 MolWeight:618 RetIndex:4395
CompName:Tetratetracontane $$ n-Tetratetracontane $$

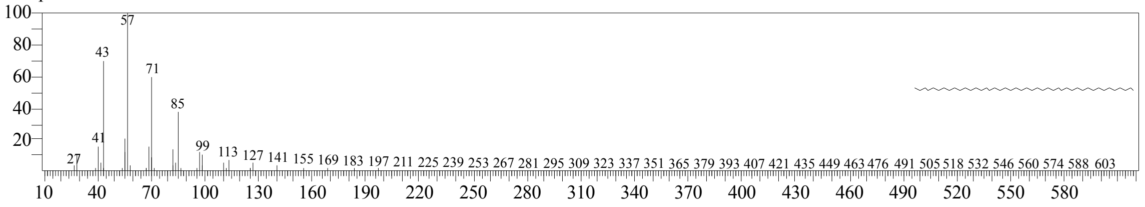

Hit#:4 Entry:26171 Library:NIST05s.LIB
SI:96 Formula:C27H56 CAS:593-49-7 MolWeight:380 RetIndex:2705
CompName:Heptacosane $$ n-Heptacosane $$

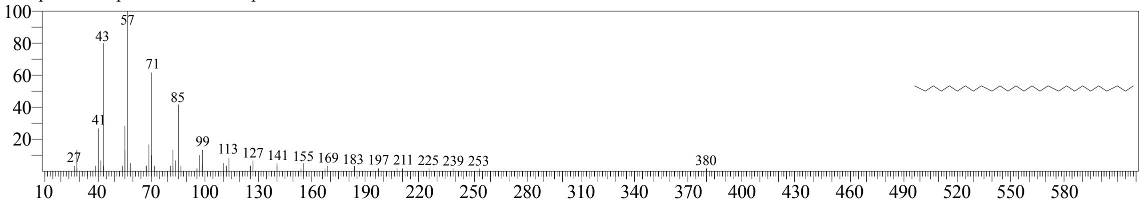

Hit#:5 Entry:26455 Library:NIST05s.LIB
SI:96 Formula:C28H58 CAS:630-02-4 MolWeight:394 RetIndex:2804
CompName:Octacosane $$ n-Octacosane $$

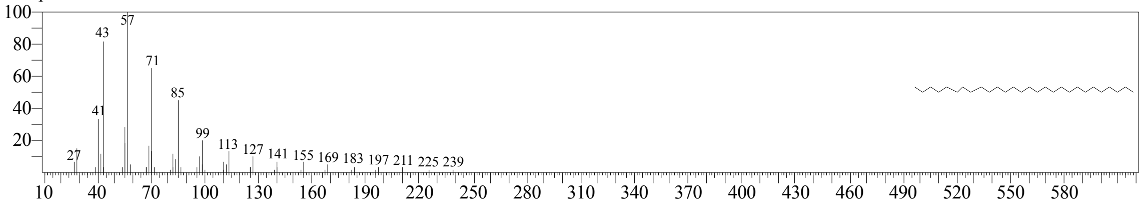

<< Target >>
Line#:27 R.Time:54.090(Scan#:10219) Retention Index:3126! MassPeaks:340
RawMode:Averaged 54.085-54.095(10218-10220) BasePeak:57.10(30287)
BG Mode:Calc. from Peak Group 1 - Event 1 Scan

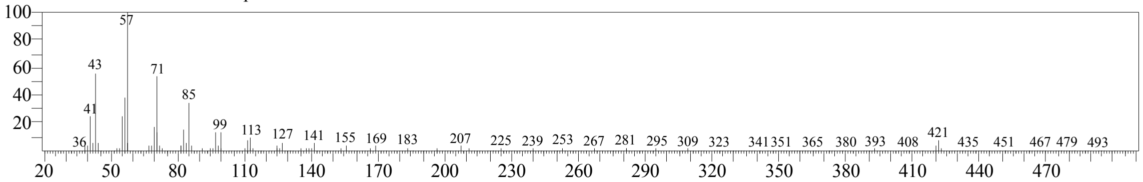

Hit#:1 Entry:161325 Library:NIST05.LIB
SI:93 Formula:C44H90 CAS:7098-22-8 MolWeight:618 RetIndex:4395
CompName:Tetratetracontane $$ n-Tetratetracontane $$

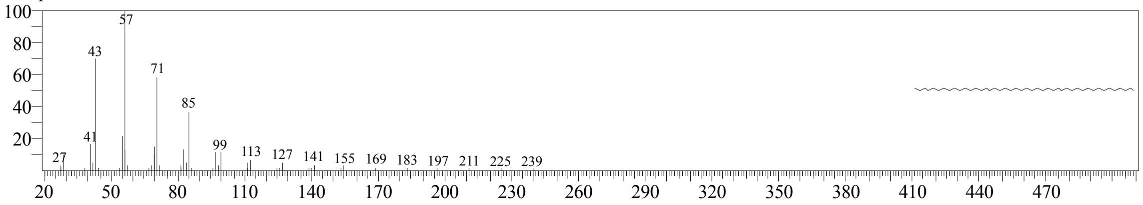

Hit#:2 Entry:27321 Library:NIST05s.LIB
SI:93 Formula:C36H74 CAS:630-06-8 MolWeight:506 RetIndex:3600
CompName:Hexatriacontane $$ n-Hexatriacontane $$

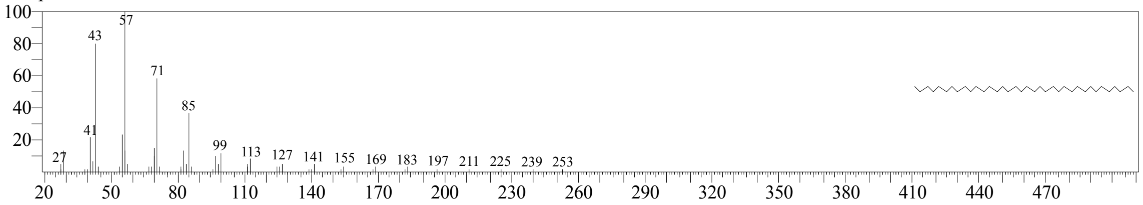

Hit#:3 Entry:26171 Library:NIST05s.LIB
SI:92 Formula:C27H56 CAS:593-49-7 MolWeight:380 RetIndex:2705
CompName:Heptacosane $$ n-Heptacosane $$

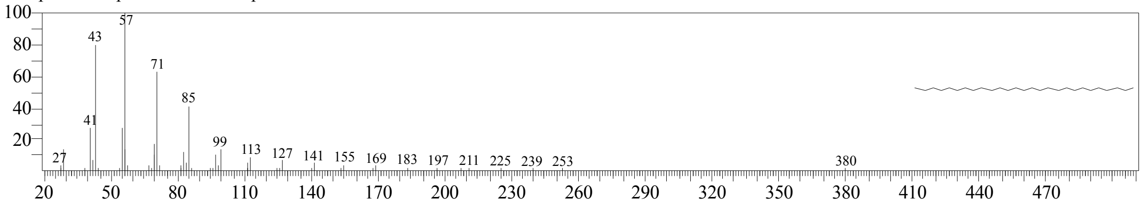

Hit#:4 Entry:27217 Library:NIST05s.LIB
SI:92 Formula:C34H70 CAS:14167-59-0 MolWeight:478 RetIndex:3401
CompName:Tetratriacontane $$ n-Tetratriacontane $$

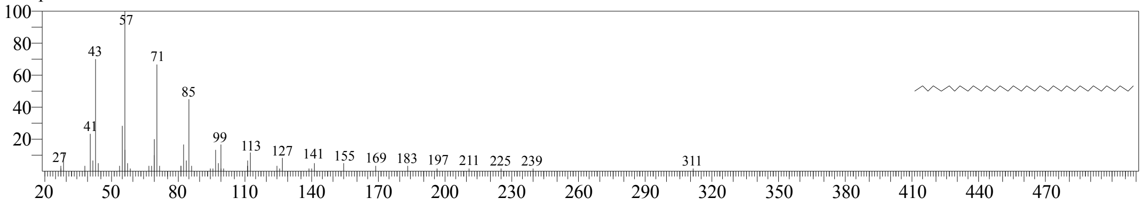

Hit#:5 Entry:26953 Library:NIST05s.LIB
SI:92 Formula:C31H64 CAS:630-04-6 MolWeight:436 RetIndex:3103
CompName:Hentriacontane $$ n-Hentriacontane $$ Untriacontane $$

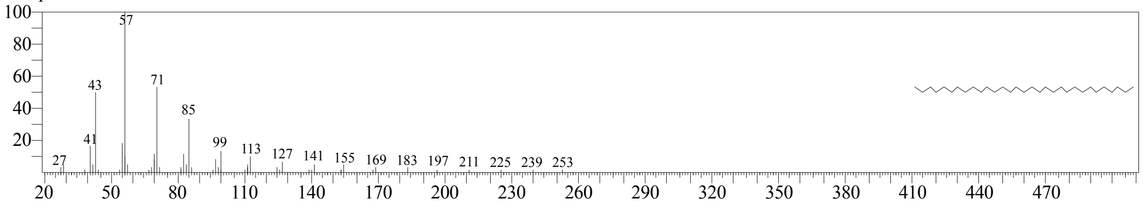

<< Target >>
Line#:28 R.Time:54.455(Scan#:10292) Retention Index:3150! MassPeaks:264
RawMode:Averaged 54.450-54.460(10291-10293) BasePeak:57.10(10325)
BG Mode:Calc. from Peak Group 1 - Event 1 Scan

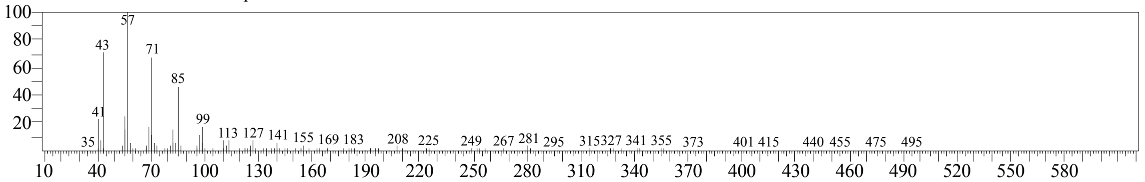

Hit#:1 Entry:27217 Library:NIST05s.LIB
SI:95 Formula:C34H70 CAS:14167-59-0 MolWeight:478 RetIndex:3401
CompName:Tetratriacontane $$ n-Tetratriacontane $$

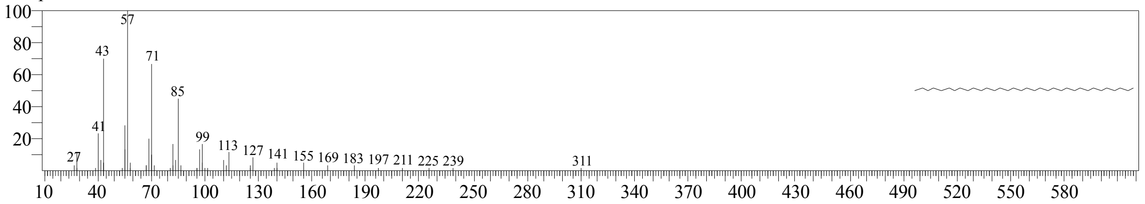

Hit#:2 Entry:185354 Library:NIST11.lib
SI:95 Formula:C29H60 CAS:0-00-0 MolWeight:408 RetIndex:2840
CompName:2-methyloctacosane

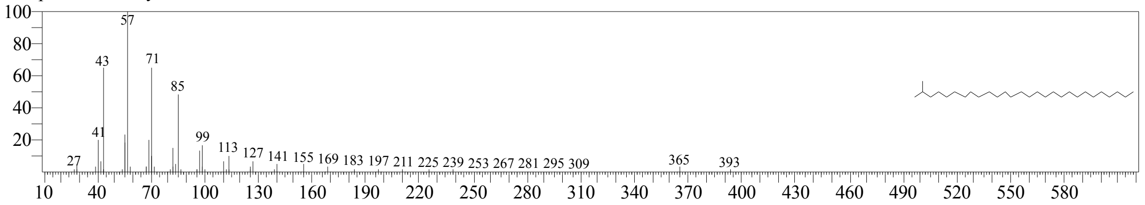

Hit#:3 Entry:30813 Library:NIST11s.lib
SI:95 Formula:C44H90 CAS:7098-22-8 MolWeight:618 RetIndex:4395
CompName:Tetratetracontane $$ n-Tetratetracontane $$

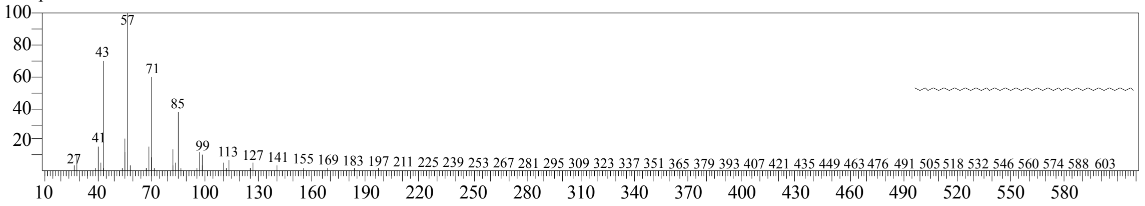

Hit#:4 Entry:26171 Library:NIST05s.LIB
SI:94 Formula:C27H56 CAS:593-49-7 MolWeight:380 RetIndex:2705
CompName:Heptacosane $$ n-Heptacosane $$

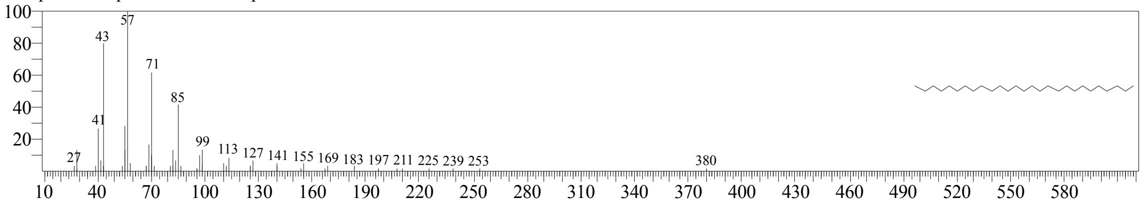

Hit#:5 Entry:27321 Library:NIST05s.LIB
SI:94 Formula:C36H74 CAS:630-06-8 MolWeight:506 RetIndex:3600
CompName:Hexatriacontane $$ n-Hexatriacontane $$

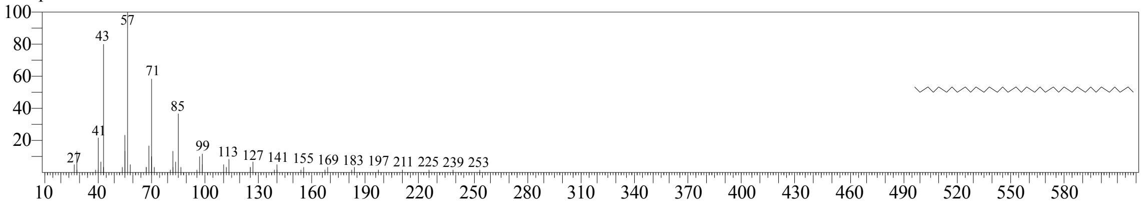

<< Target >>
Line#:29 R.Time:55.420(Scan#:10485) Retention Index:3212! MassPeaks:340
RawMode:Averaged 55.415-55.425(10484-10486) BasePeak:57.10(6351)
BG Mode:Calc. from Peak Group 1 - Event 1 Scan

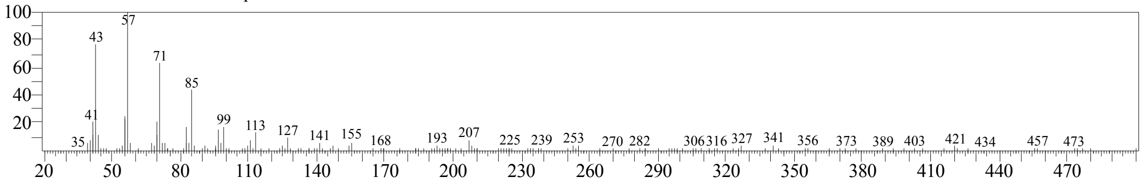

Hit#:1 Entry:27512 Library:NIST05s.LIB
SI:91 Formula:C44H90 CAS:7098-22-8 MolWeight:618 RetIndex:4395
CompName:Tetratetracontane $$ n-Tetratetracontane $$

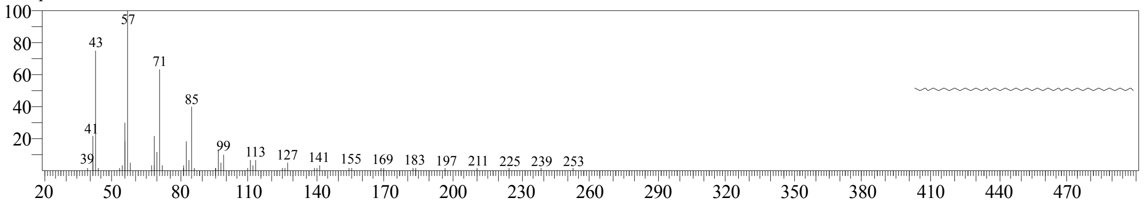

Hit#:2 Entry:26171 Library:NIST05s.LIB
SI:90 Formula:C27H56 CAS:593-49-7 MolWeight:380 RetIndex:2705
CompName:Heptacosane $$ n-Heptacosane $$

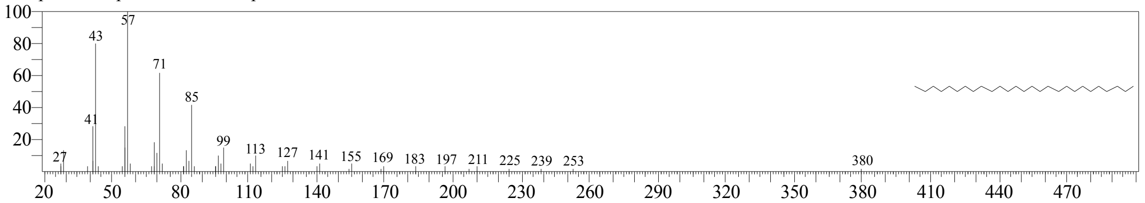

Hit#:3 Entry:185354 Library:NIST11.lib
SI:90 Formula:C29H60 CAS:0-00-0 MolWeight:408 RetIndex:2840
CompName:2-methyloctacosane

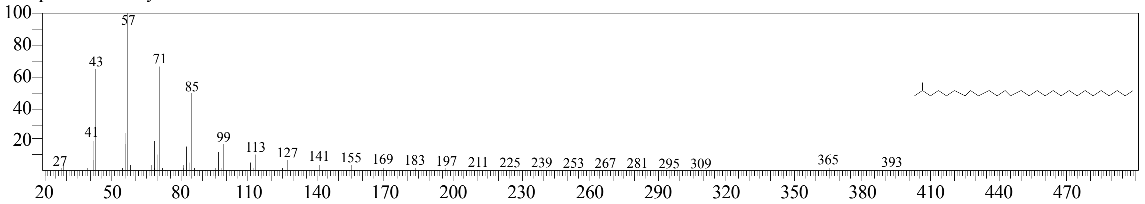

Hit#:4 Entry:27217 Library:NIST05s.LIB
SI:90 Formula:C34H70 CAS:14167-59-0 MolWeight:478 RetIndex:3401
CompName:Tetratriacontane $$ n-Tetratriacontane $$

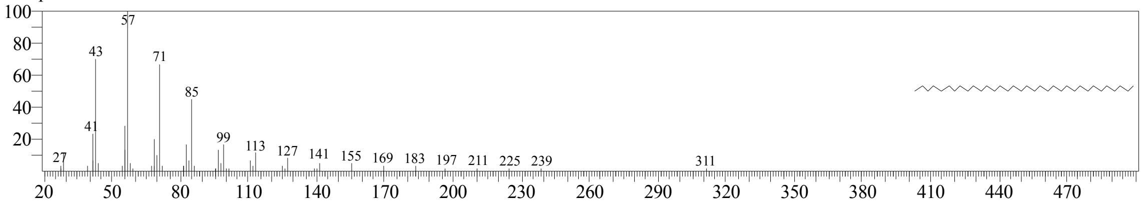

Hit#:5 Entry:26455 Library:NIST05s.LIB
SI:90 Formula:C28H58 CAS:630-02-4 MolWeight:394 RetIndex:2804
CompName:Octacosane $$ n-Octacosane $$

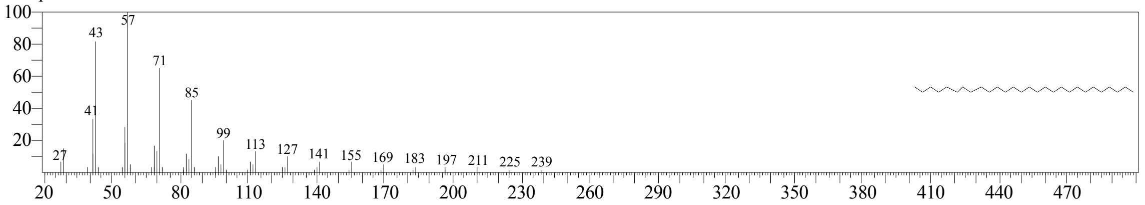

<< Target >>
Line#:30 R.Time:55.620(Scan#:10525) Retention Index:3225! MassPeaks:254
RawMode:Averaged 55.615-55.625(10524-10526) BasePeak:57.10(5036)
BG Mode:Calc. from Peak Group 1 - Event 1 Scan

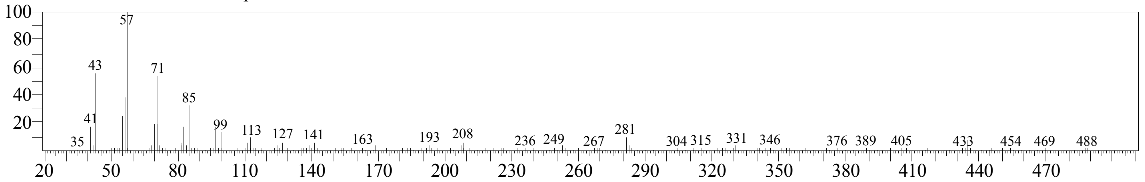

Hit#:1 Entry:161325 Library:NIST05.LIB
SI:88 Formula:C44H90 CAS:7098-22-8 MolWeight:618 RetIndex:4395
CompName:Tetratetracontane $$ n-Tetratetracontane $$

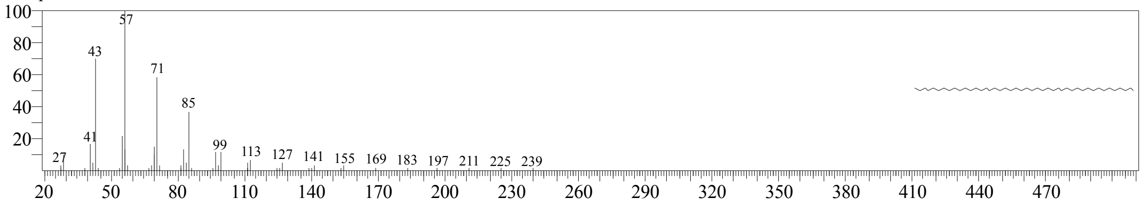

Hit#:2 Entry:161062 Library:NIST05.LIB
SI:88 Formula:C43H88 CAS:7098-21-7 MolWeight:604 RetIndex:4295
CompName:Tritetracontane

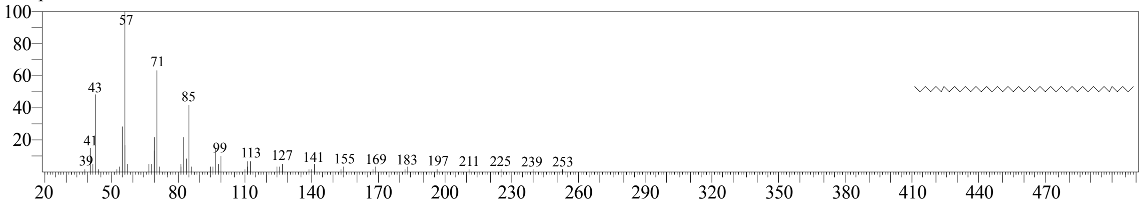

Hit#:3 Entry:137707 Library:NIST05.LIB
SI:87 Formula:C21H44O3S CAS:0-00-0 MolWeight:376 RetIndex:2732
CompName:Sulfurous acid, butyl heptadecyl ester

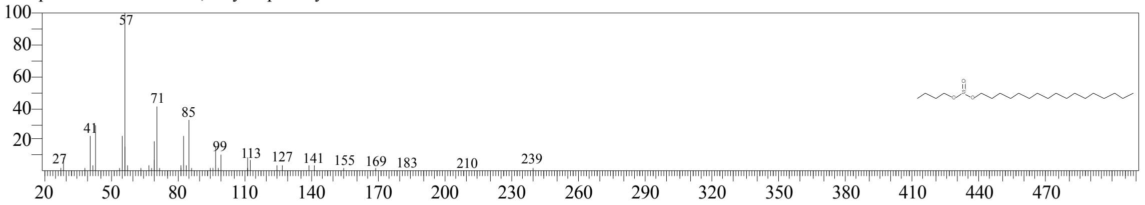

Hit#:4 Entry:27321 Library:NIST05s.LIB
SI:87 Formula:C36H74 CAS:630-06-8 MolWeight:506 RetIndex:3600
CompName:Hexatriacontane $$ n-Hexatriacontane $$

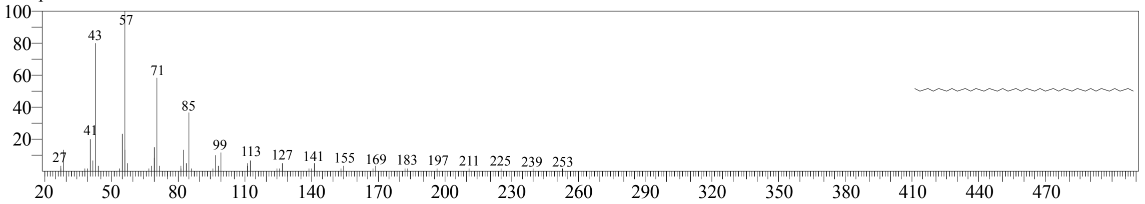

Hit#:5 Entry:26953 Library:NIST05s.LIB
SI:87 Formula:C31H64 CAS:630-04-6 MolWeight:436 RetIndex:3103
CompName:Hentriacontane $$ n-Hentriacontane $$ Untriacontane $$

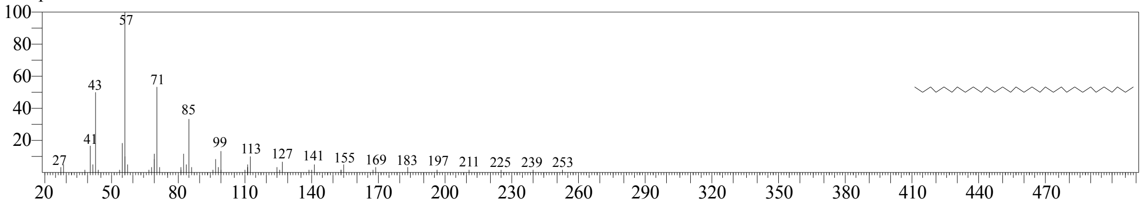

<< Target >>
Line#:31 R.Time:56.055(Scan#:10612) Retention Index:3252! MassPeaks:372
RawMode:Averaged 56.050-56.060(10611-10613) BasePeak:57.10(131798)
BG Mode:Calc. from Peak Group 1 - Event 1 Scan

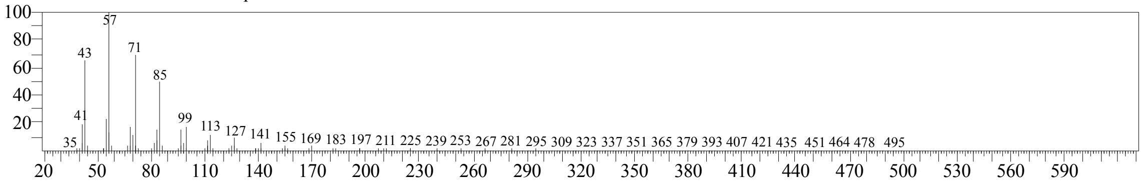

Hit#:1 Entry:210715 Library:NIST11.lib
SI:97 Formula:C44H90 CAS:7098-22-8 MolWeight:618 RetIndex:4395
CompName:Tetratetracontane $$ n-Tetratetracontane $$

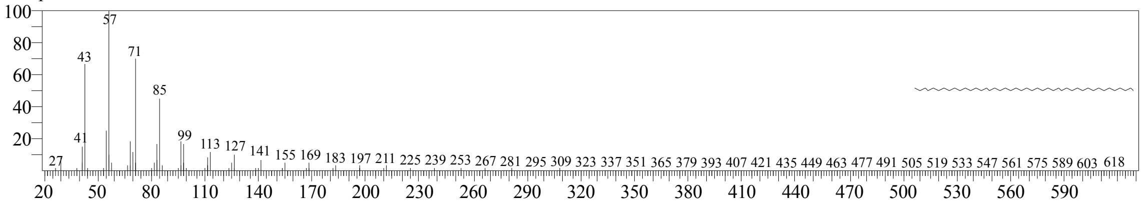

Hit#:2 Entry:185354 Library:NIST11.lib
SI:97 Formula:C29H60 CAS:0-00-0 MolWeight:408 RetIndex:2840
CompName:2-methyloctacosane

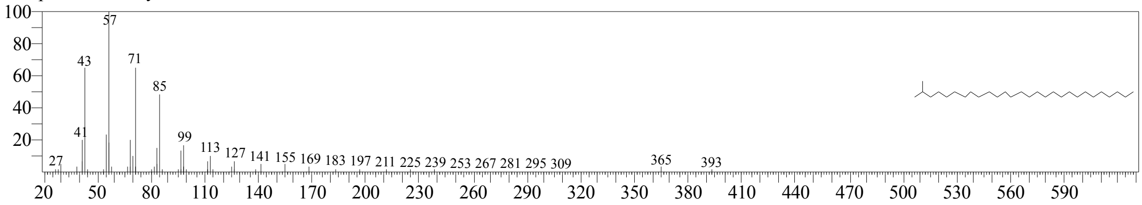

Hit#:3 Entry:27217 Library:NIST05s.LIB
SI:97 Formula:C34H70 CAS:14167-59-0 MolWeight:478 RetIndex:3401
CompName:Tetratriacontane $$ n-Tetratriacontane $$

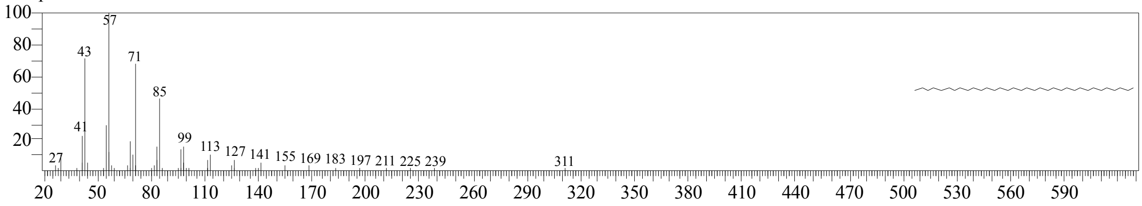

Hit#:4 Entry:26650 Library:NIST05s.LIB
SI:96 Formula:C29H60 CAS:630-03-5 MolWeight:408 RetIndex:2904
CompName:Nonacosane $$ n-Nonacosane $$

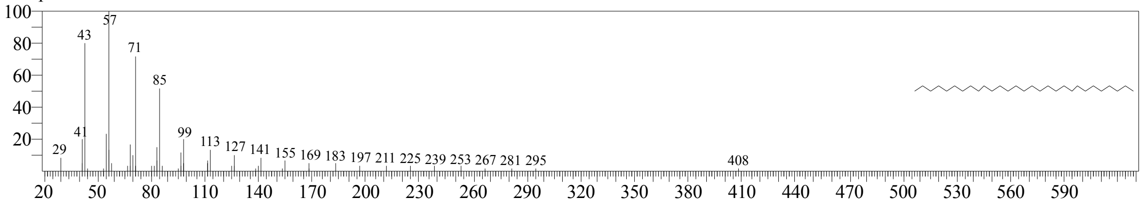

Hit#:5 Entry:26171 Library:NIST05s.LIB
SI:96 Formula:C27H56 CAS:593-49-7 MolWeight:380 RetIndex:2705
CompName:Heptacosane $$ n-Heptacosane $$

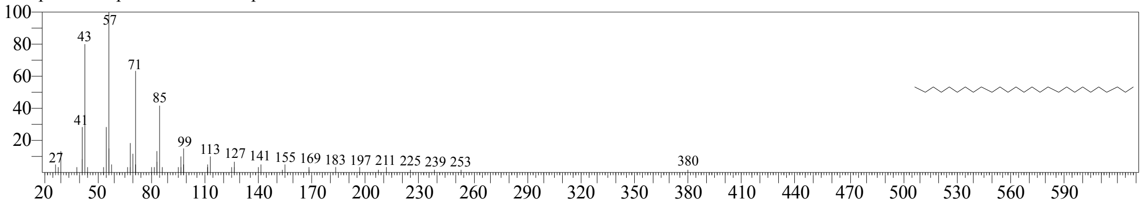

<< Target >>
Line#:32   R.Time:56.850(Scan#:10771) Retention Index:3304! MassPeaks:269
RawMode:Averaged 56.845-56.855(10770-10772) BasePeak:97.10(5932)
BG Mode:Calc. from Peak Group 1 - Event 1 Scan

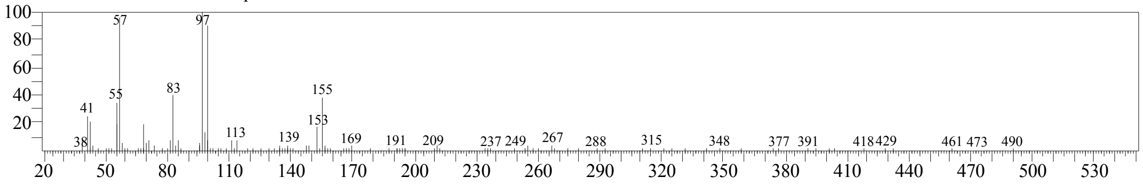

Hit#:1 Entry:61283 Library:NIST05.LIB
SI:76 Formula:C11H23Br CAS:55162-38-4 MolWeight:234 RetIndex:1283
CompName:Nonane, 2-bromo-5-ethyl- $$ 2-Bromo-5-ethylnonane # $$

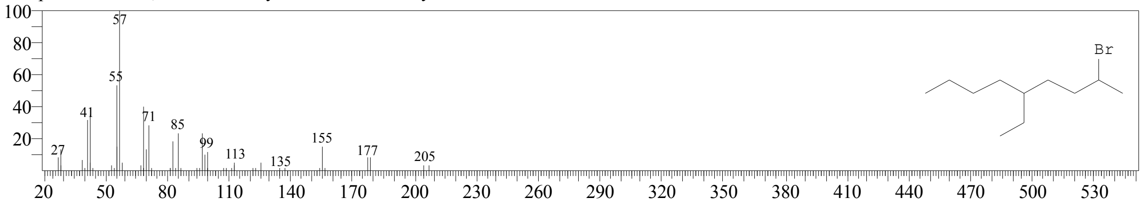

Hit#:2 Entry:161061 Library:NIST05.LIB
SI:75 Formula:C43H88 CAS:55162-61-3 MolWeight:604 RetIndex:4103
CompName:Tetracontane, 3,5,24-trimethyl- $$ 3,5,24-Trimethyltetracontane # $$

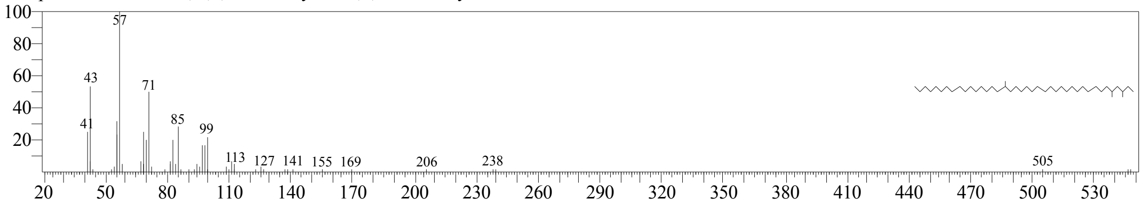

Hit#:3 Entry:116536 Library:NIST05.LIB
SI:75 Formula:C20H40O3 CAS:0-00-0 MolWeight:328 RetIndex:2188
CompName:Carbonic acid, isobutyl pentadecyl ester

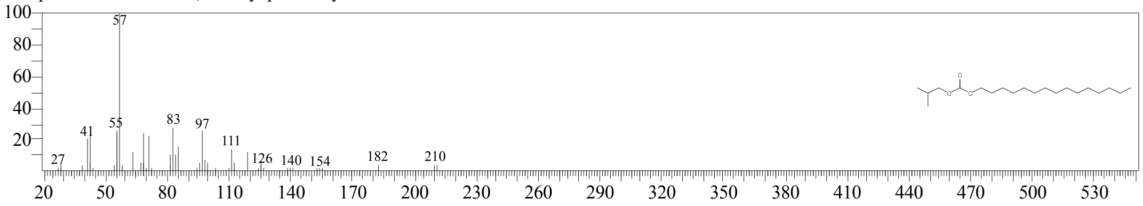

Hit#:4 Entry:171269 Library:NIST11.lib
SI:75 Formula:C21H45O3P CAS:0-00-0 MolWeight:376 RetIndex:1886
CompName:Phosphite, tris(2,4-dimethylpent-3-yl- $$ Tris(1-isopropyl-2-methylpropyl) phosphite # $$

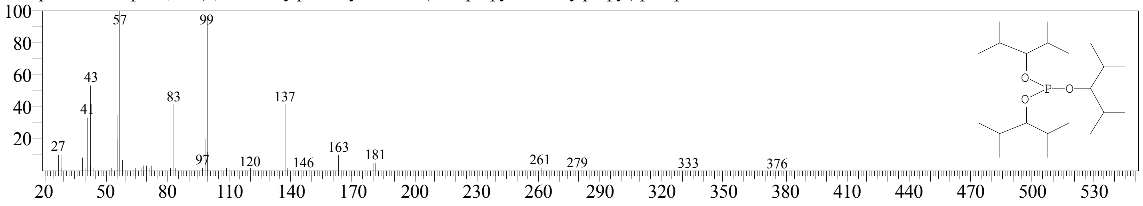

Hit#:5 Entry:137712 Library:NIST05.LIB
SI:75 Formula:C21H45O3P CAS:0-00-0 MolWeight:376 RetIndex:1886
CompName:Phosphite, tris(2,4-dimethylpent-3-yl-

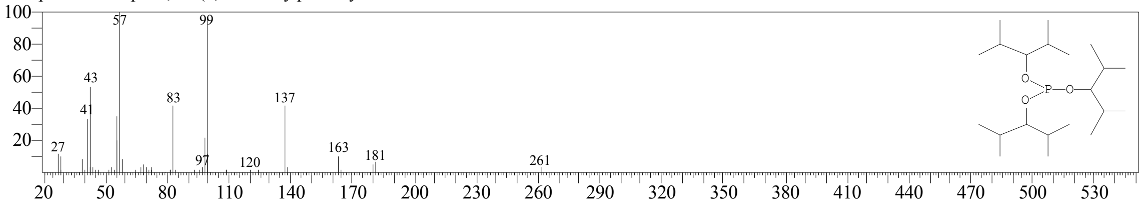

<< Target >>
Line#:33   R.Time:57.170(Scan#:10835) Retention Index:3324! MassPeaks:372
RawMode:Averaged 57.165-57.175(10834-10836) BasePeak:43.05(4237)
BG Mode:Calc. from Peak Group 1 - Event 1 Scan

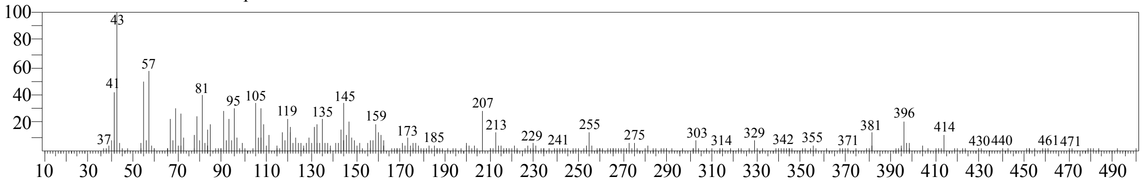

Hit#:1 Entry:29932 Library:NIST11s.lib
SI:87 Formula:C29H50O CAS:83-46-5 MolWeight:414 RetIndex:2731
CompName:.beta.-Sitosterol $$ Stigmast-5-en-3-ol, (3.beta.)- $$ Stigmast-5-en-3.beta.-ol $$ .alpha.-Dihydrofucosterol $$ .beta.-Sitosterin $$ Angelicin $$ A

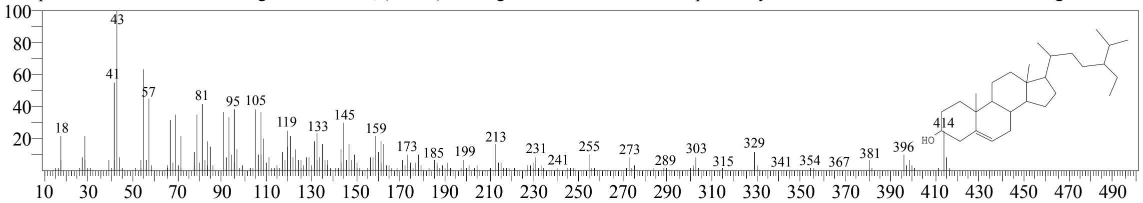

Hit#:2 Entry:187508 Library:NIST11.lib
SI:84 Formula:C29H50O CAS:83-47-6 MolWeight:414 RetIndex:2731
CompName:.gamma.-Sitosterol $$ Stigmast-5-en-3-ol, (3.beta.,24S)- $$ Stigmast-5-en-3.beta.-ol, (24S)- $$ Clionasterol $$ Fucosterol, .beta.-dihydro- $$ 2

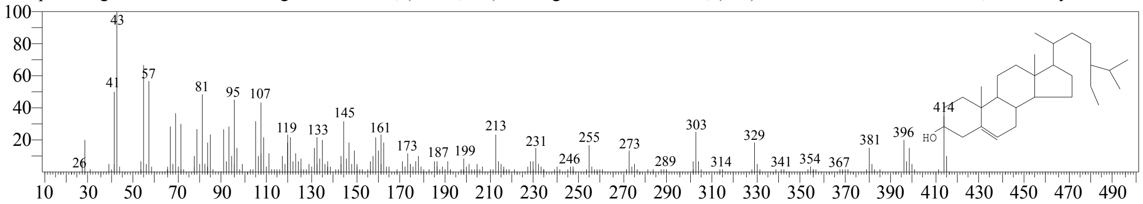

Hit#:3 Entry:160902 Library:NIST05.LIB
SI:83 Formula:C41H72O2 CAS:1989-52-2 MolWeight:596 RetIndex:3929
CompName:Cholest-5-en-3-ol (3.beta.)-, tetradecanoate $$ Cholesterol, myristate $$ Cholesteryl myristate $$ Cholesteryl tetradecanoate $$ Cholestryl myri

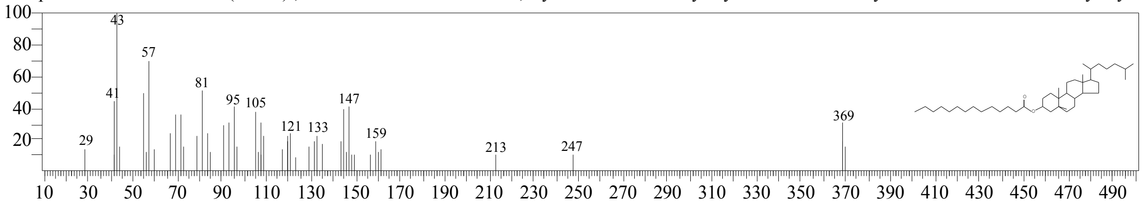

Hit#:4 Entry:27074 Library:NIST05s.LIB
SI:83 Formula:C31H50O2 CAS:4651-48-3 MolWeight:454 RetIndex:2879
CompName:Stigmasta-5,22-dien-3-ol, acetate, (3.beta.)- $$ Stigmasta-5,22-dien-3.beta.-ol, acetate $$ Stigmasterol acetate $$ Stigmasteryl acetate $$ 3.beta.

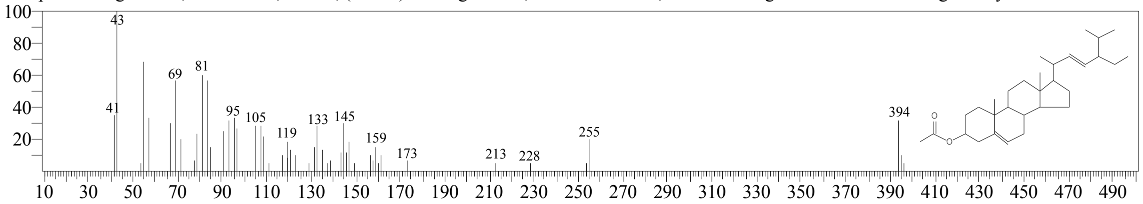

Hit#:5 Entry:144899 Library:NIST05.LIB
SI:83 Formula:C28H48O CAS:474-62-4 MolWeight:400 RetIndex:2632
CompName:Campesterol $$ Ergost-5-en-3-ol, (3.beta.,24R)- $$ Ergost-5-en-3.beta.-ol, (24R)- $$ (24R)-5-Ergosten-3.beta.-ol $$ Campesterin $$ Campestr

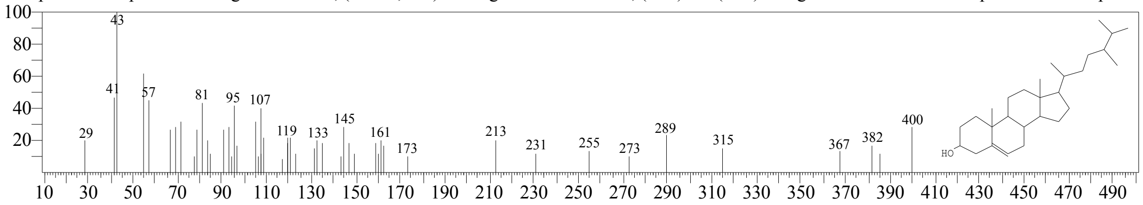

<< Target >>
Line#:34 R.Time:57.405(Scan#:10882) Retention Index:3339! MassPeaks:331
RawMode:Averaged 57.400-57.410(10881-10883) BasePeak:57.10(22014)
BG Mode:Calc. from Peak Group 1 - Event 1 Scan

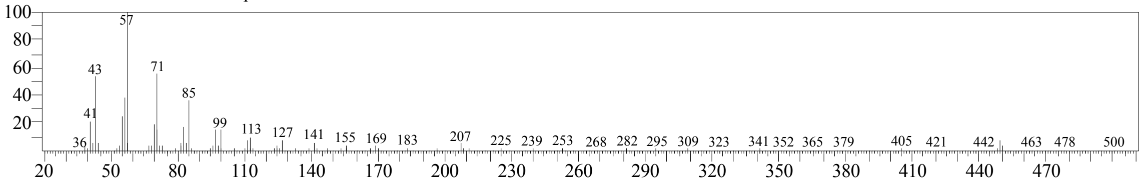

Hit#:1 Entry:161325 Library:NIST05.LIB
SI:93 Formula:C44H90 CAS:7098-22-8 MolWeight:618 RetIndex:4395
CompName:Tetratetracontane $$ n-Tetratetracontane $$

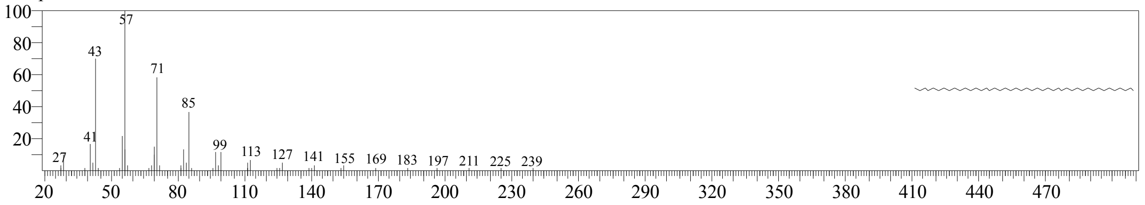

Hit#:2 Entry:27217 Library:NIST05s.LIB
SI:93 Formula:C34H70 CAS:14167-59-0 MolWeight:478 RetIndex:3401
CompName:Tetratriacontane $$ n-Tetratriacontane $$

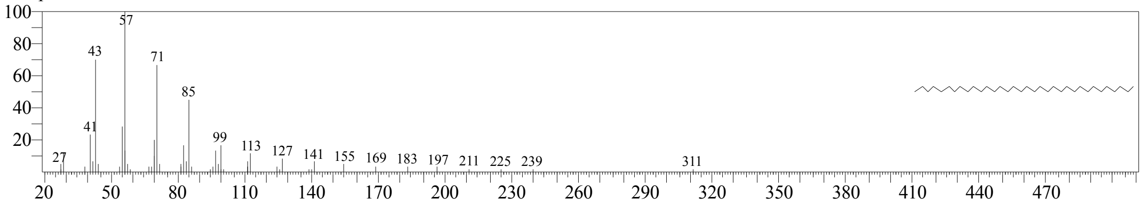

Hit#:3 Entry:161062 Library:NIST05.LIB
SI:92 Formula:C43H88 CAS:7098-21-7 MolWeight:604 RetIndex:4295
CompName:Tritetracontane

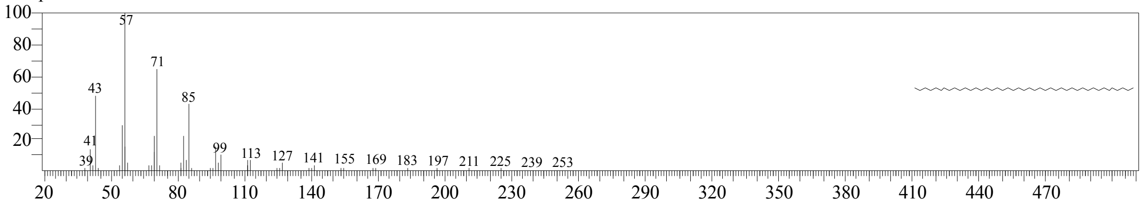

Hit#:4 Entry:26953 Library:NIST05s.LIB
SI:92 Formula:C31H64 CAS:630-04-6 MolWeight:436 RetIndex:3103
CompName:Hentriacontane $$ n-Hentriacontane $$ Untriacontane $$

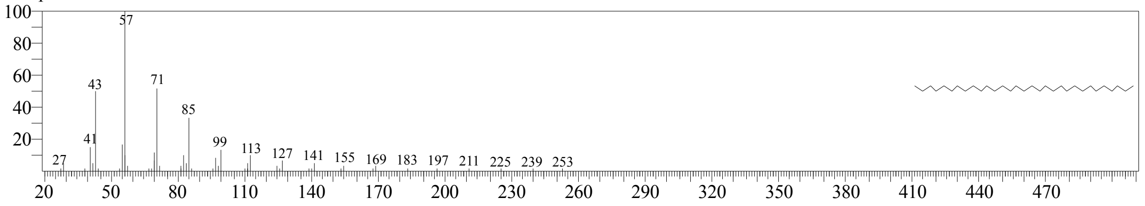

Hit#:5 Entry:27321 Library:NIST05s.LIB
SI:92 Formula:C36H74 CAS:630-06-8 MolWeight:506 RetIndex:3600
CompName:Hexatriacontane $$ n-Hexatriacontane $$

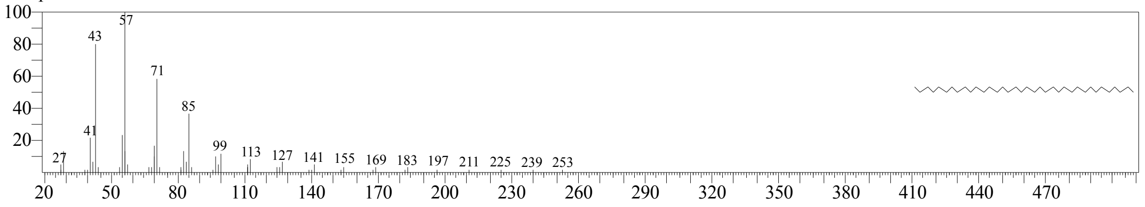

<< Target >>
Line#:35 R.Time:57.940(Scan#:10989) Retention Index:3374! MassPeaks:303
RawMode:Averaged 57.935-57.945(10988-10990) BasePeak:97.10(36176)
BG Mode:Calc. from Peak Group 1 - Event 1 Scan

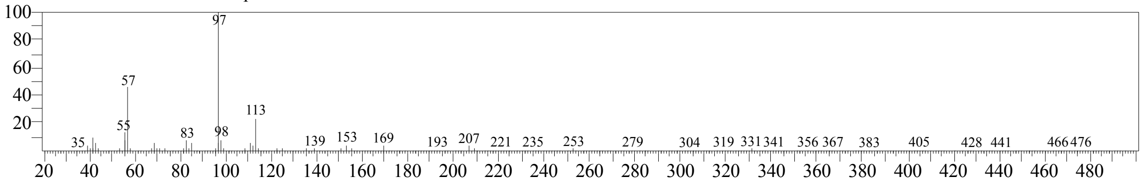

Hit#:1 Entry:150324 Library:NIST05.LIB
SI:83 Formula:C25H50O3S CAS:0-00-0 MolWeight:430 RetIndex:3193
CompName:Sulfurous acid, cyclohexylmethyl octadecyl ester

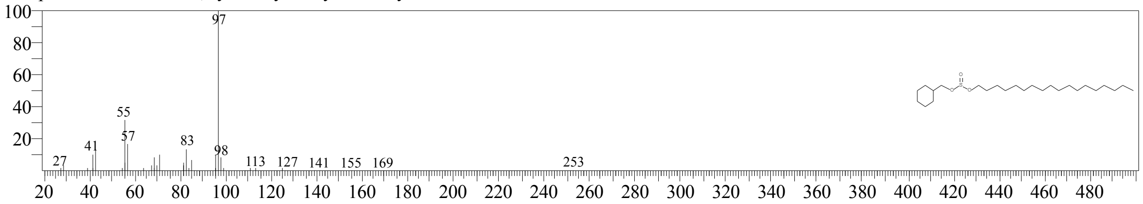

Hit#:2 Entry:192100 Library:NIST11.lib
SI:83 Formula:C25H50O3S CAS:0-00-0 MolWeight:430 RetIndex:3193
CompName:Sulfurous acid, cyclohexylmethyl octadecyl ester

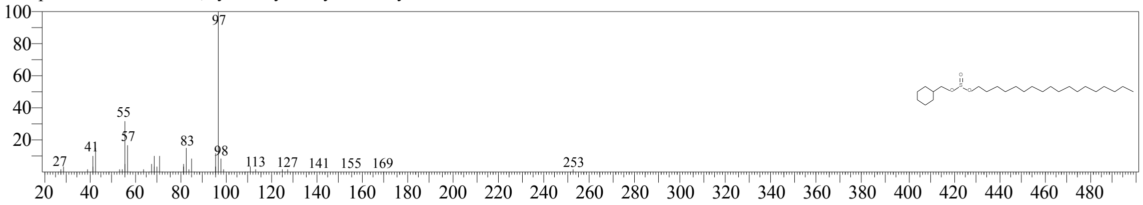

Hit#:3 Entry:141493 Library:NIST05.LIB
SI:83 Formula:C22H44O3S CAS:0-00-0 MolWeight:388 RetIndex:2895
CompName:Sulfurous acid, cyclohexylmethyl pentadecyl ester

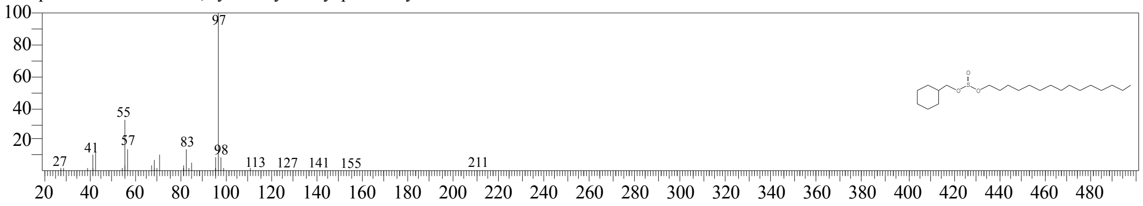

Hit#:4 Entry:177164 Library:NIST11.lib
SI:83 Formula:C22H44O3S CAS:0-00-0 MolWeight:388 RetIndex:2895
CompName:Sulfurous acid, cyclohexylmethyl pentadecyl ester

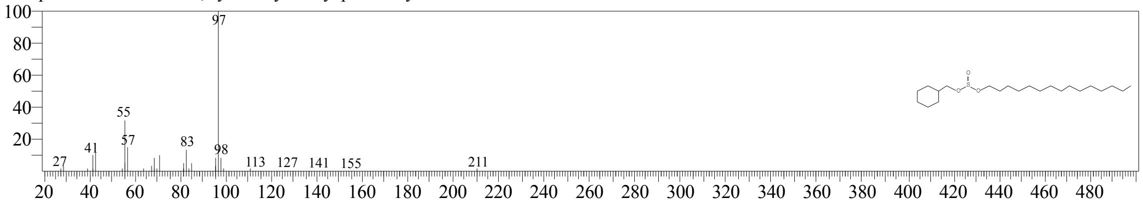

Hit#:5 Entry:125328 Library:NIST05.LIB
SI:83 Formula:C19H38O3S CAS:0-00-0 MolWeight:346 RetIndex:2597
CompName:Sulfurous acid, cyclohexylmethyl dodecyl ester

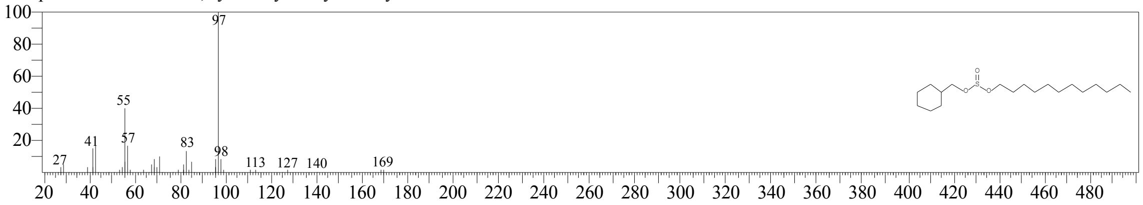

<< Target >>
Line#:36   R.Time:58.805(Scan#:11162) Retention Index:3429! MassPeaks:306
RawMode:Averaged 58.800-58.810(11161-11163) BasePeak:57.10(12951)
BG Mode:Calc. from Peak Group 1 - Event 1 Scan

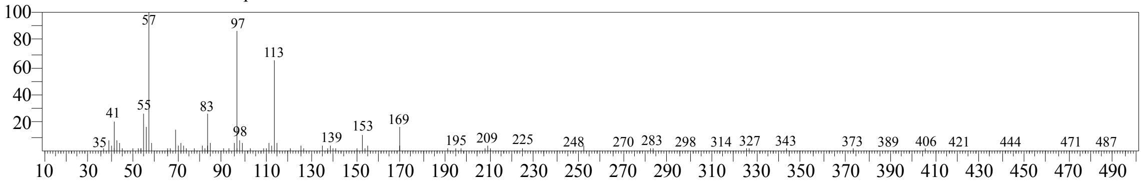

Hit#:1 Entry:24910 Library:NIST11.lib
SI:79 Formula:C12H24 CAS:123-48-8 MolWeight:168 RetIndex:1030
CompName:3-Heptene, 2,2,4,6,6-pentamethyl- $$ 2,2,4,6,6-Pentamethylheptene-3 $$ 2,2,4,6,6-Pentamethyl-3-heptene $$ 2,2,4,6,6-Pentamethyl-3-heptene,

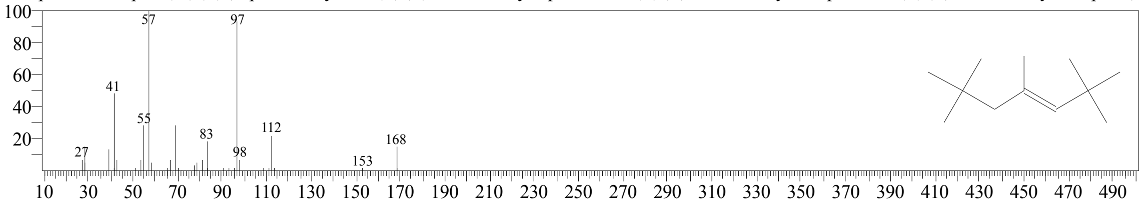

Hit#:2 Entry:142072 Library:NIST05.LIB
SI:76 Formula:C22H46O3S CAS:0-00-0 MolWeight:390 RetIndex:2831
CompName:Sulfurous acid, butyl octadecyl ester

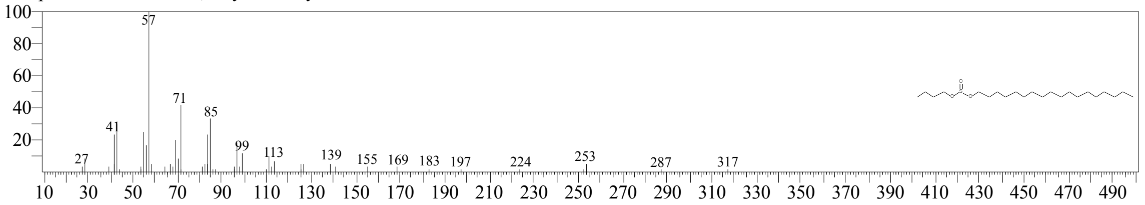

Hit#:3 Entry:137707 Library:NIST05.LIB
SI:76 Formula:C21H44O3S CAS:0-00-0 MolWeight:376 RetIndex:2732
CompName:Sulfurous acid, butyl heptadecyl ester

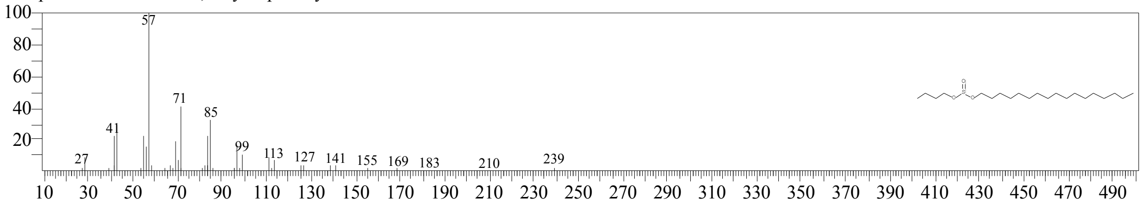

Hit#:4 Entry:135513 Library:NIST05.LIB
SI:76 Formula:C23H46O3 CAS:0-00-0 MolWeight:370 RetIndex:2487
CompName:Carbonic acid, isobutyl octadecyl ester

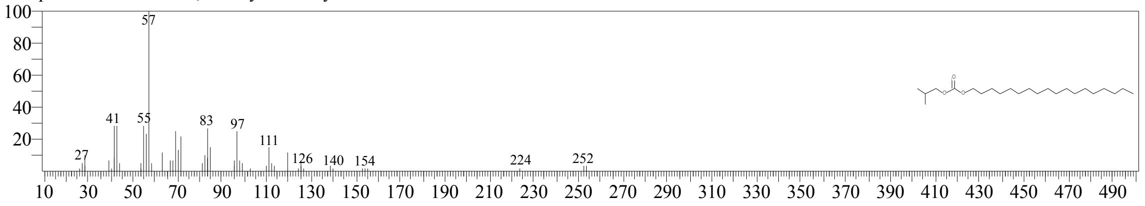

Hit#:5 Entry:132214 Library:NIST05.LIB
SI:76 Formula:C20H42O3S CAS:0-00-0 MolWeight:362 RetIndex:2632
CompName:Sulfurous acid, butyl hexadecyl ester

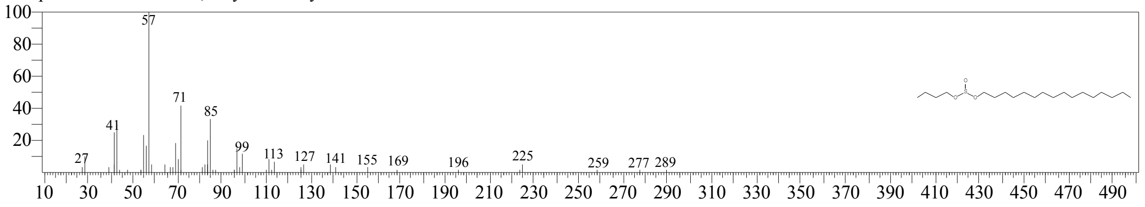

<< Target >>
Line#:37 R.Time:60.050(Scan#:11411) Retention Index:3509! MassPeaks:310
RawMode:Averaged 60.045-60.055(11410-11412) BasePeak:57.10(6449)
BG Mode:Calc. from Peak Group 1 - Event 1 Scan

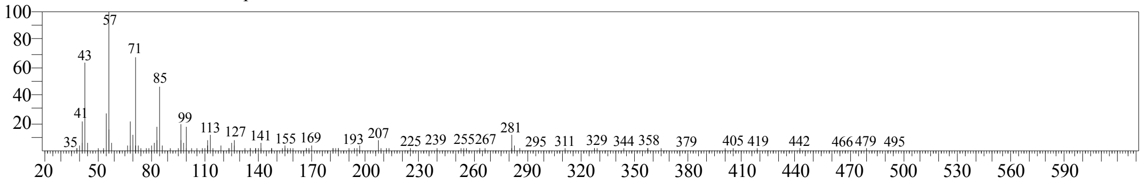

Hit#:1 Entry:27217 Library:NIST05s.LIB
SI:92 Formula:C34H70 CAS:14167-59-0 MolWeight:478 RetIndex:3401
CompName:Tetratriacontane $$ n-Tetratriacontane $$

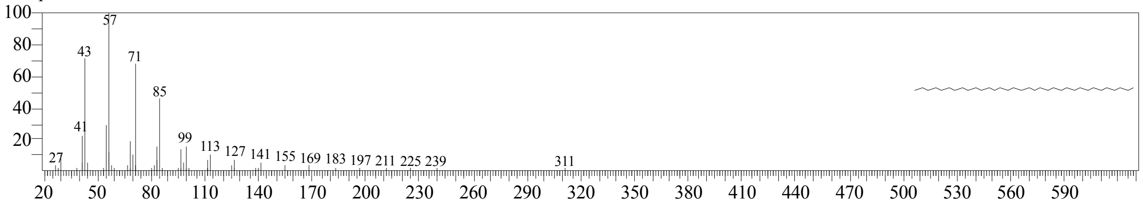

Hit#:2 Entry:210715 Library:NIST11.lib
SI:92 Formula:C44H90 CAS:7098-22-8 MolWeight:618 RetIndex:4395
CompName:Tetratetracontane $$ n-Tetratetracontane $$

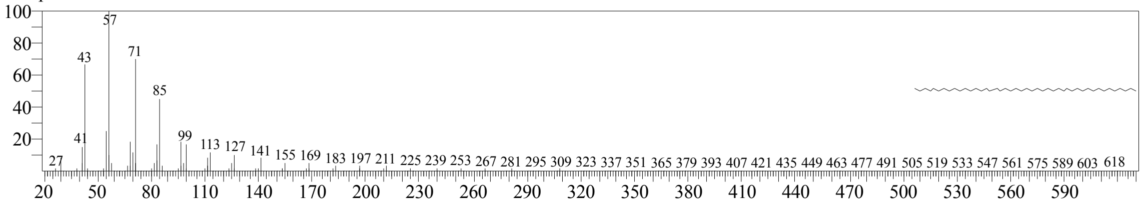

Hit#:3 Entry:185354 Library:NIST11.lib
SI:91 Formula:C29H60 CAS:0-00-0 MolWeight:408 RetIndex:2840
CompName:2-methyloctacosane

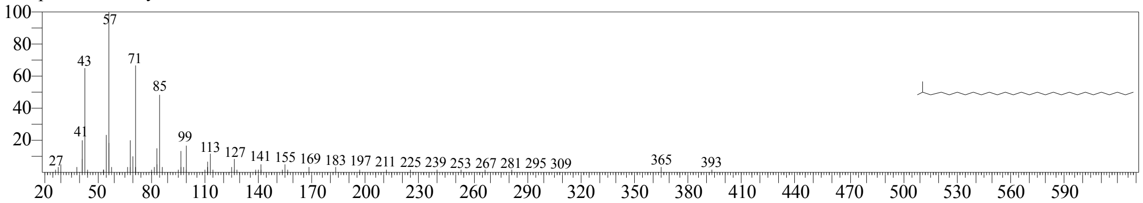

Hit#:4 Entry:30555 Library:NIST11s.lib
SI:90 Formula:C35H72 CAS:630-07-9 MolWeight:492 RetIndex:3500
CompName:Pentatriacontane $$ n-Pentatriacontane $$

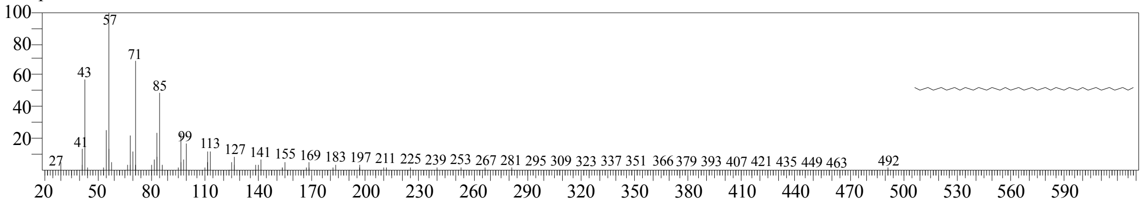

Hit#:5 Entry:26171 Library:NIST05s.LIB
SI:90 Formula:C27H56 CAS:593-49-7 MolWeight:380 RetIndex:2705
CompName:Heptacosane $$ n-Heptacosane $$

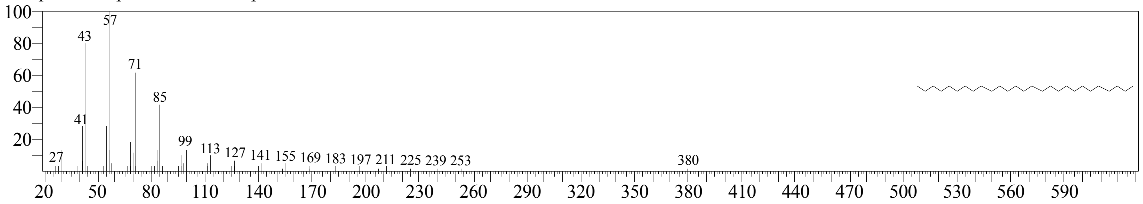

Supplement: Supplementary file 1 — Supplementary Information. [file 41598_2022_20412_MOESM1_ESM.pdf]
